# Design and Understanding of Adaptive Hydrogenation Catalysts Triggered by the H_2_/CO_2_–Formic Acid Equilibrium

**DOI:** 10.1021/jacs.4c06765

**Published:** 2024-09-25

**Authors:** Yuyan Zhang, Natalia Levin, Liqun Kang, Felix Müller, Mirijam Zobel, Serena DeBeer, Walter Leitner, Alexis Bordet

**Affiliations:** † Max Planck Institute for Chemical Energy Conversion, Stiftstraße 34-36, 45470 Mülheim an der Ruhr, Germany; ‡ Institute for Technical and Macromolecular Chemistry, RWTH Aachen University, 52074 Aachen, Germany; § Institute of Crystallography, RWTH Aachen University, 52074 Aachen, Germany

## Abstract

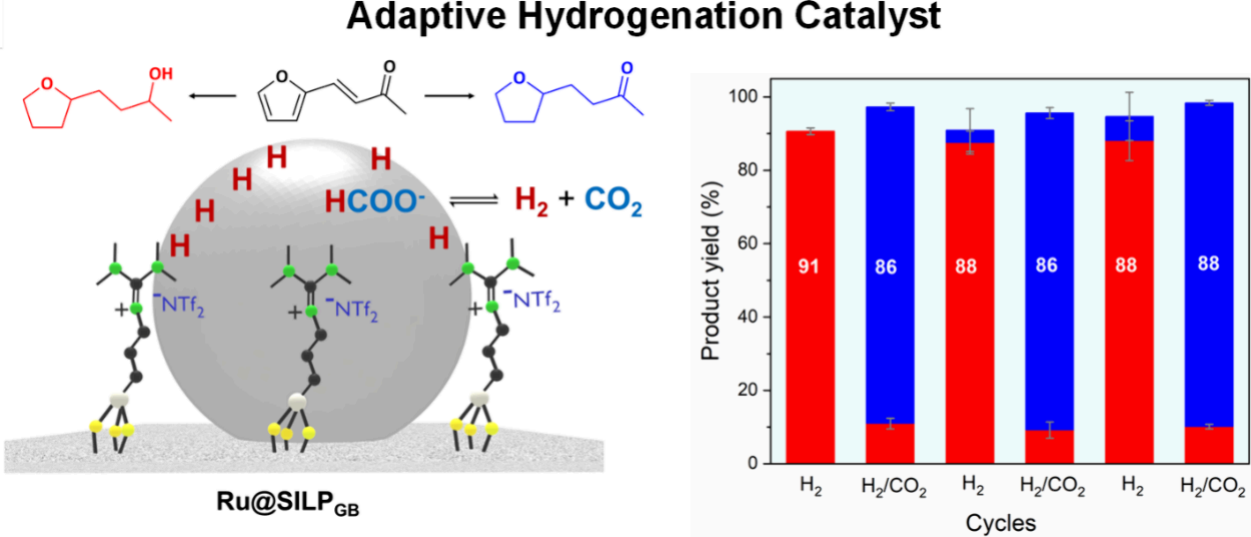

An adaptive catalytic system for selective hydrogenation was developed exploiting the H_2_ + CO_2_  HCOOH equilibrium for reversible, rapid, and robust on/off switch of the ketone hydrogenation activity of ruthenium nanoparticles (Ru NPs). The catalyst design was based on mechanistic studies and DFT calculations demonstrating that adsorption of formic acid to Ru NPs on silica results in surface formate species that prevent C=O hydrogenation. Ru NPs were immobilized on readily accessible silica supports modified with guanidinium-based ionic liquid phases (Ru@SILP_GB_) to generate in situ sufficient amounts of HCOOH when CO_2_ was introduced into the H_2_ feed gas for switching off ketone hydrogenation while maintaining the activity for hydrogenation of olefinic and aromatic C=C bonds. Upon shutting down the CO_2_ supply, the C=O hydrogenation activity was restored in real time due to the rapid decarboxylation of the surface formate species without the need for any changes in the reaction conditions. Thus, the newly developed Ru@SILP_GB_ catalysts allow controlled and alternating production of either saturated alcohols or ketones from unsaturated substrates depending on the use of H_2_ or H_2_/CO_2_ as feed gas. The major prerequisite for design of adaptive catalytic systems based on CO_2_ as trigger is the ability to shift the H_2_ + CO_2_  HCOOH equilibrium sufficiently to exploit competing adsorption of surface formate and targeted functional groups. Thus, the concept can be expected to be more generally applicable beyond ruthenium as the active metal, paving the way for next-generation adaptive catalytic systems in hydrogenation reactions more broadly.

## 1 Introduction

As the production of fuels and chemicals is shifting from the use of fossil to renewable resources, challenges associated with the diversity and variability in energy and feedstock supplies are emerging.^1−3^ Consequently, catalysis research and development require innovative solutions to cope with the dynamics associated with the use of alternative energy resources to establish postfossil value chains.^4,5^ This is especially important when considering the use of “green” molecular hydrogen (H_2_) to convert renewable carbon feedstocks to essential value-added products (e.g., fuels, commodities, fine chemicals, agrochemicals, pharmaceuticals, etc.).^5−7^ A high degree of process flexibility or even adaptivity can be expected to be beneficial for the use of renewable resources, in particular to cope with quality variation in chemical feedstocks, and intermittent energy supply.^8,9^

In this context, the concept of *adaptive catalysis* takes inspiration from nature, where the reactivity of catalytic systems is modulated reversibly by various stimuli to promote countless series and parallel chemical transformations without interferences.^10,11^

The development of adaptive catalytic systems with the ability to control their performance through the application of external stimuli (e.g., temperature, chemical reactions, photochemical irradiation, redox switches, etc.) has attracted increasing attention in the past years.^12−14^ For example, following the pioneering work from Feringa et al. on light driven molecular rotors,^15^ the light-induced *cis–trans* isomerization of double bonds has been extensively studied as photoswitch to control the reactivity of organocatalysts, metal complexes, and metal nanoparticles (NPs).^16−18^ Most switchable catalysts developed so far attempt to control catalytic activity in response to fluctuating energy supply.^19−23^ Controlling product selectivity in a reversible manner can offer additional potential to enable customized production^8,9^ while coping with rapidly changing feedstock, market demand for products, or energy supply.^17,18,24−26^

Chemical modification of active sites on metal surfaces is a frequently applied tool to open or close individual pathways in complex catalytic networks.^12^ In order to be *adaptive*, the modification needs to be reversible, rapid and robust (“*R*^3^ rule”) allowing to switch between different modifications of a given catalyst material.^14^ A possible molecular process to fulfill this criteria is offered by the reversible reaction of H_2_ and CO_2_ to produce formic acid or formate.^27−30^ We have recently exploited this equilibrium to develop an adaptive catalytic system composed of ruthenium NPs immobilized on a tertiary amine-functionalized polymer grafted silica support (Ru@PGS).^24^ The selectivity switch in the hydrogenation of furan derivatives^24^ and bicyclic heteroaromatics^26^ was associated with the generation of ammonium formate species under H_2_/CO_2_, which decomposed upon heating under pure H_2_. The actual mechanism of action remained so far elusive, however. In addition, the elaborate synthesis of the polymeric structure of the surface molecular modifier and the heat treatment required to decompose the ammonium formate salt for regeneration of the original activity of Ru NPs limited the practical use of such systems.

**Figure 1:**
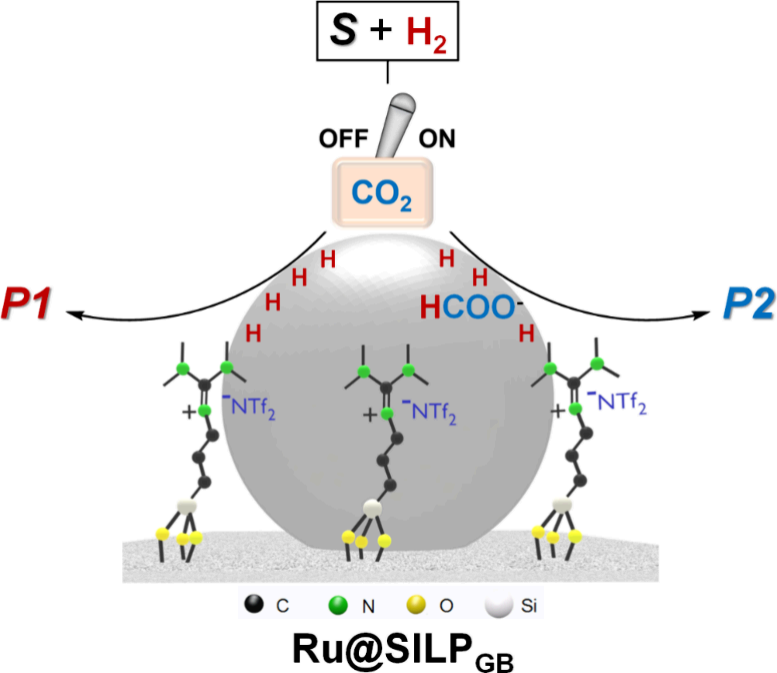
Adaptive catalytic system using in situ generated formic acid/formate as molecular trigger, illustrated for the Ru@SILP_GB_ catalyst. *S* = substrate, *P* = product.

In the present study, the influence of formic acid and formate species on the C=O hydrogenation activity of Ru NPs is investigated through mechanistic studies and DFT calculations. The acquired fundamental understanding serves as basis for the development of a new generation of simpler adaptive catalytic systems exploiting directly the reversible hydrogenation of CO_2_ to formic acid to control the selectivity of Ru NPs in aromatic ketone hydrogenation in real time (Figure 1).

## 2 Results and Discussion

### 2.1 Mechanistic Studies and DFT Calculations

**Figure 2:**
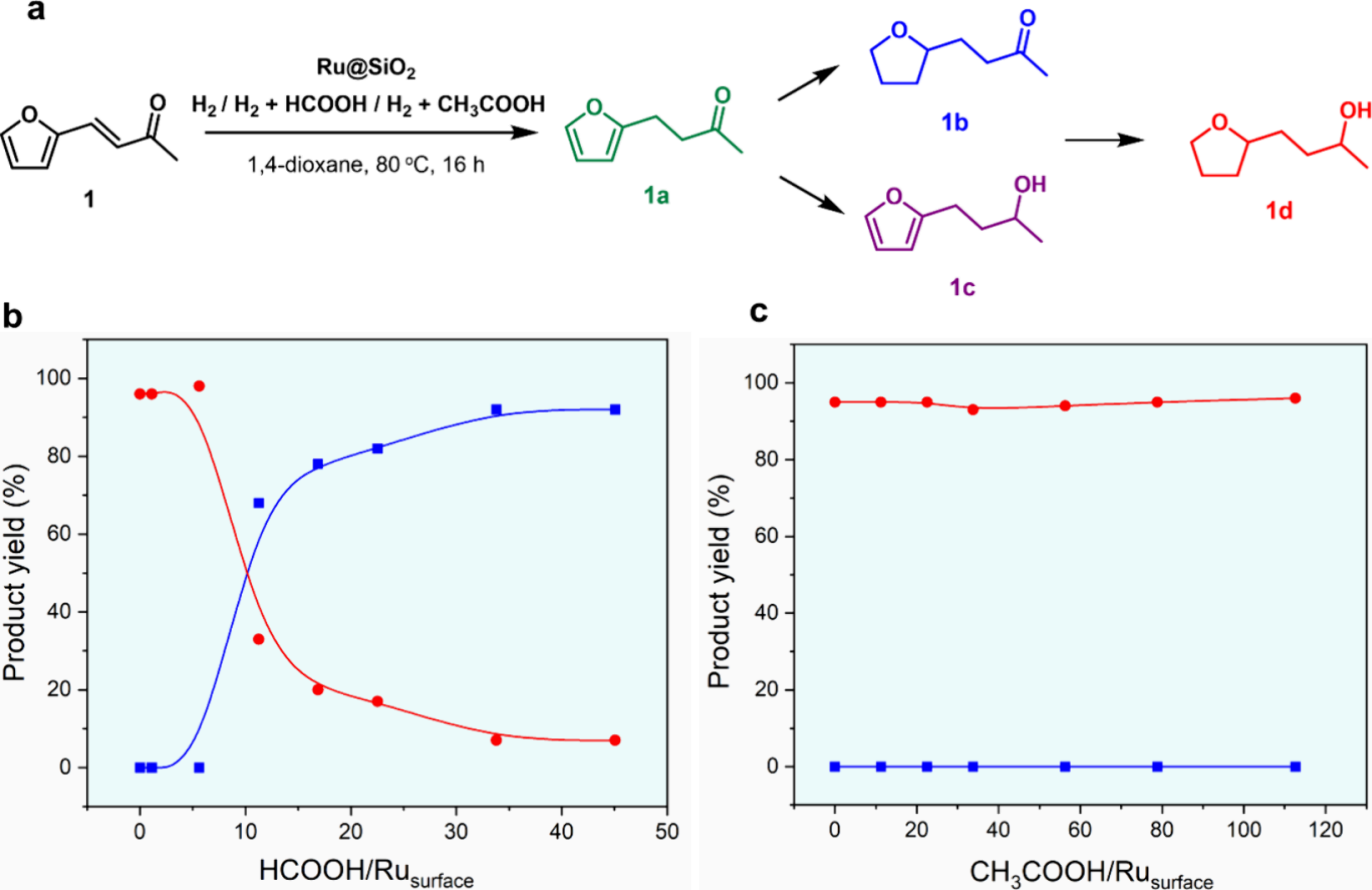
Hydrogenation of **1** using Ru@SiO_2_. (a) Reaction scheme; (b) hydrogenation under H_2_ with various amounts of formic acid as additive; (c) hydrogenation under H_2_ with various amounts of acetic acid as additive. Reaction conditions: Ru@SiO_2_ (19 mg, 0.007 mmol Ru), furfuralacetone (**1**, 0.25 mmol, 35 equiv), 1,4-dioxane (1 mL), 80 °C, 16 h, 500 rpm, H_2_ (15 bar), the red curve and blue curve are for products **1d** and product **1b**, respectively. HCOOH/Ru_surface_ = molar ratio between HCOOH and Ru centers exposed at the surface of Ru NPs, see SI for details.

The hydrogenation of biomass-derived furfuralacetone (**1**) by 2.3 nm Ru NPs immobilized on SiO_2_ (Ru@SiO_2_, characterization described in the Figure S1 and Table S1) was used as a model reaction to investigate the potential impact of formic acid on the reactivity of Ru NPs. The hydrogenation of **1** can proceed via two reaction pathways possibly yielding four products (**1a**, **1b**, **1c**, **1d**, Figure 2a). Catalytic reactions were conducted using 10 mL stainless steel high-pressure reactors equipped with a pressure gauge and heated in temperature-controlled aluminum blocks. Reaction conditions were set to 80 °C, 15 bar of H_2_, 16 h, and 1,4-dioxane as solvent.

Under pure H_2_, full hydrogenation of **1** to the saturated alcohol **1d** was observed with Ru@SiO_2_, which is the expected reactivity of Ru NPs under these conditions (Figure 2b,c).^24^ In sharp contrast, adding liquid formic acid to the solvent prior to the hydrogenation of **1** under pure H_2_ led to high yields of the saturated ketone **1b** (Figure 2b). Initial addition of formic acid at a concentration of 22 mmol·L^–1^ (corresponding to a HCOOH/Ru molar ratio of 3, or HCOOH/Ru_surface_ = 10 when considering only Ru centers exposed at the surface, see SI for details) was necessary to effectively suppress ketone hydrogenation (69% yield of **1b**). It is worth noting that liquid formic acid partially decomposed under pure H_2_ as feed gas as evidenced by a decrease in HCOOH concentration with time under these reaction conditions (80 °C, 15 bar H_2_, Figure S2). Remarkably, introducing acetic acid did not show the same controlling effect as formic acid to suppress ketone hydrogenation. When acetic acid was used as an additive in the hydrogenation of **1** under pure H_2_, quantitative yields of the saturated alcohol **1d** were formed even at high acetic acid/Ru_surface_ ratios (up to 112, Figure 2c). These results indicate a specific action of formic acid on the catalytic performance of the Ru NPs resulting in the suppression of ketone hydrogenation activity. Notably, formic acid alone is capable of initiating the selectivity switch with Ru@SiO_2_, indicating that an amine functionality to generate ammonium formate species is not essential for the catalyst design.

Previous studies on the adsorption of formic acid on SiO_2_-supported metal NPs (e.g., Pd, Cu) evidenced the dissociative adsorption of formic acid at the metal surface to give bidentate formate species, alongside molecular adsorption on the SiO_2_ support.^31−33^ Interestingly, comparable studies performed using acetic acid evidenced a favored molecular adsorption at the SiO_2_ surface through H-bonding and silyl ester formation, and little to no formation of bidentate acetates species at the surface of supported metal NPs (e.g., Pd@SiO_2_).^34−36^

FTIR characterization of Ru@SiO_2_ after formic acid and acetic acid adsorption were found consistent with previous findings (Figure S3).^31−36^ In particular, formic acid adsorption resulted in a band at 1561 cm^–1^ characteristic of COO^–^ in bidentate formate species, along with a C=O band at 1715 cm^–1^ corresponding to adsorbed molecular formic acid (Figure S3a). In contrast, acetic acid was found only in the molecularly adsorbed form (C=O band at 1730 cm^–1^) and none of the bands characteristic of acetate species (e.g., 1420, 1550, 1620 cm^–1^) was observed (Figure S3b), similar to what has been reported for Pd@SiO_2_ catalysts.^35^ This striking difference in the behavior of formic acid and acetic acid on Ru@SiO_2_ is possibly related to the difference in their O–H bond dissociation free energies (377 and 469 kJ/mol, respectively).^37^

These data suggest that the suppression of C=O hydrogenation activity induced by HCOOH is specifically linked to its dissociative adsorption at the Ru NPs surface in the presence of SiO_2_. To further investigate this hypothesis, the potential competitive adsorption of HCOOH and tetrahydrofuran ketones at the surface of Ru NPs was investigated by DFT calculations (details provided in SI).

The reactive surface of the Ru NPs was represented as extended surfaces of Ru, where the Ru(0001) plane was selected as the active surface, since it is thermodynamically stable and often used to represent catalytically active surfaces.^38,39^ The heteroaromatic 2-acetonylfuran (**2**) and saturated 2-acetonyltetrahydrofuran (**2a**) were selected as simple models to investigate their adsorption energies on the Ru surface in comparison to formic acid/formate species (Table 1). Different adsorption sites are available on Ru(0001) (Figure S4), and for each species investigated, several possible adsorption modes were considered. Heteroaromatic compound **2** can adsorb at the Ru(0001) surface via two dominant modes of similar adsorption energies (−2.08 and −2.09 eV, Table 1). The formation of a 2-acetonylfuran bridge is less likely, with a lower adsorption energy (−1.19 eV, Table 1). The saturated model **2a** can adsorb at the Ru(0001) surface via two different modes of similar absorption energies (−1.13 and −1.21 eV, Table 1) involving a direct interaction of the carbonyl with the Ru surface. While the molecular adsorption of HCOOH on metal surfaces has been observed at low temperatures (e.g., 80 K),^40,41^ it is known to adsorb dissociatively through a proton transfer reaction on metal surfaces under conditions relevant to our study.^34−36,42^ The optimization of the dissociative adsorption of HCOOH affords a formate-like species interacting with the surface through the two oxygen atoms in a perpendicular fashion and a H atom in an *hcp* hollow site, exhibiting a typical bidentate formate species,^40,41,43^ with an overall adsorption energy of −1.8 eV (Table 1). Attempts to optimize this species on different starting adsorption modes converged toward the same optimized structure.

**Table 1:**
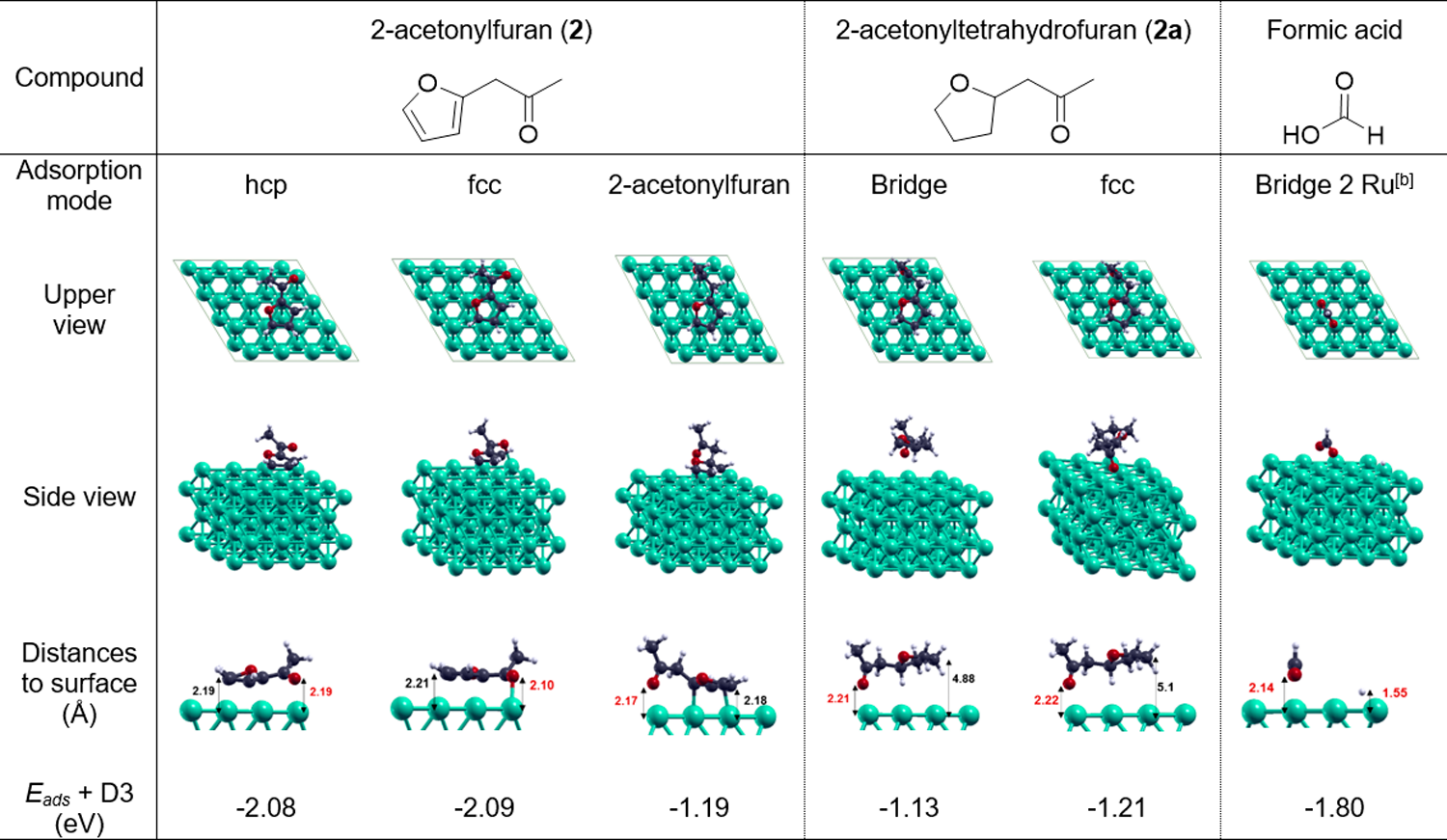
Adsorption Modes of 2-Acetonylfuran (**2**), 2-Acetonyltetrahydrofuran (**2a**), and Formic Acid on a Ru(0001) Surface and Corresponding Adsorption Energies^a^

a Details can be found in the Supporting Information file.

b Dissociative adsorption.

The calculated adsorption energies indicate that the dissociative adsorption of HCOOH at the surface of Ru NPs is weaker than the adsorption of heteroaromatic **2**, but stronger than the adsorption of saturated **2a**. These data are in line with the observation that the C=C bond hydrogenation in furan rings is not inhibited upon addition of CO_2_ (vide infra). In turn, the strongly reduced activity of Ru@SiO_2_ for the hydrogenation of ketone-containing tetrahydrofuran derivatives under H_2_/CO_2_ can thus be rationalized by a competitive adsorption at the surface of Ru NPs between ketone intermediates and formate species resulting from the in situ hydrogenation of CO_2_ to formic acid. These new mechanistic insights indicate that generation of formic acid rather than ammonium formate should be sufficient to control the C=O hydrogenation ability of Ru NPs on silica support.

### 2.2 Catalyst Design, Synthesis, and Characterization

The results of the mechanistic and DFT studies suggest that the full reversibility of the H_2_ + CO_2_  HCOOH equilibrium can be potentially used as a rapid trigger to initiate selectivity switches in hydrogenation, with clear benefits as compared to previously reported systems^24^ relying on complex polymeric surface molecular modifiers and more stable ammonium formates species. However, its effectiveness relies on the capacity of the considered catalytic system to build up and stabilize significant concentrations of formic acid under the reaction conditions considered.^44^ In this context, ionic liquids (ILs) are known for their potential of strong solute–solvent interactions^45^ (e.g., Coulombic, H-bonding, van der Waals) and were found to shift the endergonic CO_2_ hydrogenation equilibrium favorably toward production of formic acid.^46,47^ In particular, guanidinium-based ionic liquids have been reported to combine a high affinity for CO_2_^48,49^ with an exceptional potential for stabilization of formic acid.^50,51^ Consequently, our catalyst design focused on a guanidinium-based supported ionic liquid phase (SILP_GB_) as matrix for immobilization of Ru NPs.^52−54^ The Ru NPs were chosen to act on one hand (under H_2_) as hydrogenation catalysts for the substrate furfuralacetone **1**, and on the other hand (under H_2_/CO_2_) also as CO_2_ hydrogenation catalysts producing the expected molecular trigger formic acid stabilized by the guanidinium-based surface molecular modifier.^54^

**Figure 3:**
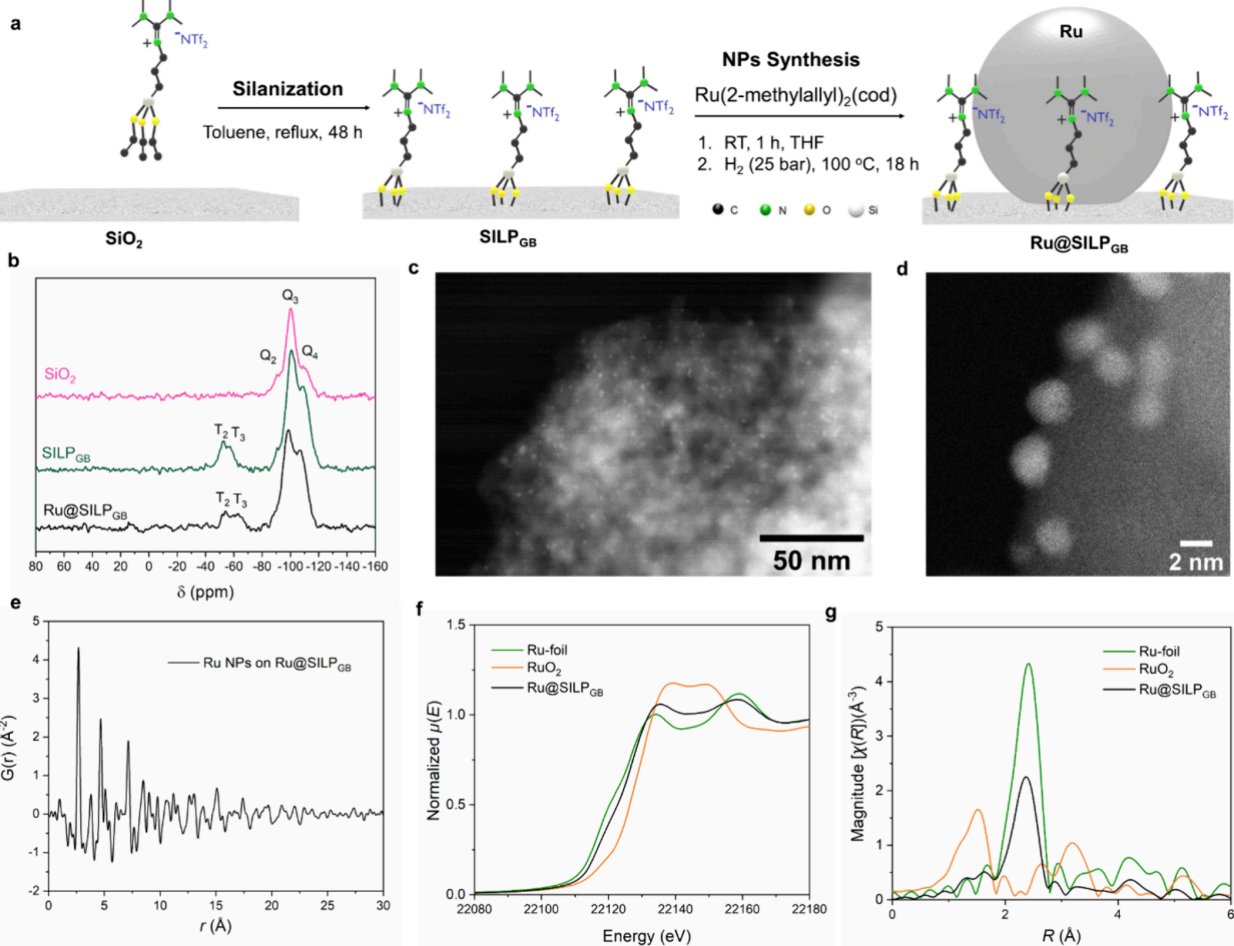
(a) Illustration of the design and preparation approach for Ru@SILP_GB_; (b) solid state ^29^Si CP-MAS of SiO_2_ (pink), SILP_GB_ (green), and Ru@SILP_GB_ (black); (c, d) HAADF-STEM images of Ru@SILP_GB_; (e) PDF G(r) of Ru NPs on Ru@SILP_GB_ (after subtraction of SILP_GB_ signal); (f) *k*^2^-weighted R-space FT-EXAFS spectra and (g) Ru K-edge XANES spectra (normalized) plot in Magnitude without phase correction) for Ru-foil, Ru@SILP_GB_ and RuO_2_ (green, black and orange curves, respectively).

The silane-functionalized guanidinium-based ionic liquid [1,1,3,3-tetramethyl-2-[3-(triethoxysilyl)propyl] guanidium bis(trifluoromethylsulfonyl)imide] (IL_GB_) was prepared by adapting protocols from the literature,^53^ and its structure was confirmed by NMR spectroscopy (Figures S5 and S6). The silanization of IL_GB_ on dehydroxylated SiO_2_ was achieved following a previously reported procedure^52,53^ and afforded the corresponding supported ionic liquid phase (SILP_GB_) with a loading of IL-like molecular modifiers of 0.58 mmol·g^–1^ (Figure 3a). Importantly, the preparation of SILP_GB_ involves less and safer steps (4), better atom economy (AE = 24.5%) and cost efficiency (ca. 2.5 Euro/g) than that of the PGS support material (5 steps, AE = 4%, ca. 19 Euro/g) previously used^24^ for adaptive catalysis (see SI for detailed analysis, Figures S7–S8 and Tables S2–S3). For the immobilization of Ru NPs, SILP_GB_ was subjected to wet impregnation with a solution of [Ru(2-methylallyl)_2_(cod)] (where cod = 1,5-cyclooctadiene) in tetrahydrofuran (THF). After removal of the solvent in vacuo, the dried powder was treated under an atmosphere of H_2_ (25 bar) at 100 °C for 18 h, giving the Ru@SILP_GB_ catalyst as a fine black powder with a Ru loading of 3.5 wt % (or 0.35 mmol·g^–1^) as determined by inductively coupled plasma optical emission spectroscopy (ICP-OES, Table S1).

N_2_ physisorption analysis (Table S1) showed a decrease in the surface area of SILP_GB_ and Ru@SILP_GB_ (308 and 302 m^2^·g^–1^ respectively, calculated using Brunner–Emmett–Teller (BET) theory) in comparison to the starting SiO_2_ (453 m^2^·g^–1^), as expected due to the functionalization with IL_GB_. Solid-state ^29^Si CP-MAS NMR analysis of SILP_GB_ and Ru@SILP_GB_ (Figure 3b) showed the presence of two types of Si species: 1) tetra-functionalized (Q) Si with signals at −109 (Q_4_ = Si(OSi)_4_) and −100 ppm (Q_3_ = Si(OSi)_3_OH); and 2) trifunctionalized (T) Si with signals at −53 (T_2_ = R-Si(OSi)_2_OR’) and −62 ppm (T_3_ = R-Si(OSi)_3_). The T_2_ and T_3_ signals correspond to the Si atoms of IL_GB_ covalently bound to the SiO_2_ surface, confirming the successful chemisorption of IL_GB_. Thermogravimetric analysis (TGA) performed under Ar showed that the SILP_GB_ and Ru@SILP_GB_ materials are thermally stable up to 280 °C (Figure S9). The transmission IR spectrum of Ru@SILP_GB_ (Figure S10) exhibited several bands characteristic of the structure of IL_GB_, including N–H stretching at 3500–3100 cm^–1^, C–H stretching at 2950 cm^–1^, and C=N and C–N stretching at 1620 and 1420 cm^–1^, respectively.^55,56^ This indicates that the structure of IL_GB_ was not affected by chemisorption nor by Ru NPs deposition. Bands at 1980 and 1873 cm^–1^ were attributed to silica overtone bands.^56^

High-angle annular dark field scanning transmission electron microscopy (HAADF-STEM, Figure 3c,d) analysis confirmed the formation of small Ru NPs (diameter = 1.3 ± 0.4 nm, histogram provided in Figure S11) well-dispersed on the SILP_GB_ support material. Lattice spacings (Table S4) of the Ru NPs were determined from the Fast Fourier Transformation (FFT, Figure S12) of the bright field STEM images (BF-STEM), which correspond well to *hcp* Ruthenium (P6_3_/*mmc*).

Powder X-ray diffraction (PXRD) failed to provide insights into the structure of these very small Ru NPs, giving broad diffuse scattering rather than sharp Bragg peaks.^57^ Therefore, the atomic pair distribution function (PDF) technique was utilized, by applying a Fourier transformation on the total scattering data (Bragg peaks plus diffuse scattering). This method elucidates the nanostructures of the sample by representing them as a histogram of interatomic distances in real space (Figures 3e, S13 and S14). The PDF of Ru NPs in Ru@SILP_GB_ (Figure 3e) is calculated from synchrotron PXRD data (Figure S13), and a refinement (see Figure S13f) confirms the presence of *hcp* Ru NPs with a P6_3_/*mmc* space group, consistent with the FFT analysis. Potential interactions between Ru NPs and the IL layer of SILP_GB_ cannot be concluded from the PDF data.

Ru K-edge X-ray absorption fine structure (XAFS) study of the Ru@SILP_GB_ was performed to investigate the electronic and geometric structures of Ru NPs (Figure 3f,g). The X-ray absorption near edge structure (XANES) spectrum of Ru@SILP_GB_ shows an absorption edge position (estimated by the energy position at 0.5 of normalized absorption) of 22119.3 eV, approximately 2.1 eV higher than that of Ru(0) in Ru metal foil (22117.2 eV), but 6.7 eV lower than that of Ru(IV) in RuO_2_ (22123.9 eV). This suggests that Ru is mainly in the metallic state with only traces of oxidation in Ru@SILP_GB_. Quantitative EXAFS fitting results of Ru@SILP_GB_ (Table S5, Figure S15) showed Ru–Ru scattering with a coordination number (C.N.) of 9.1 ± 0.9 at 2.68 ± 0.01 Å, and an additional Ru–O scattering with C.N. of 1.3 ± 0.5 at 1.97 ± 0.03 Å, which is in good agreement with the slight oxidation observed from the XANES analysis.

Two reference catalysts comprising different surface modifiers were prepared using the same methods for Ru NP deposition and surface modification: Ru@Si-Dec (Ru NPs on SiO_2_ functionalized with decyl chains),^58^ and Ru@SILP_IM_ (Ru NPs on an imidazolium-based SILP).^54^ Their structural and physicochemical properties were found very similar to that of Ru@SILP_GB_ and Ru@SiO_2_ (Table S1, Figures S16 and S17) allowing for a direct comparison of the surface modification on the adaptivity of the catalyst materials.

### 2.3 Catalytic Study

#### 2.3.1 Reactivity of Ru@SILP_GB_ with H_2_/CO_2_–Hydrogenation of CO_2_ and Formic Acid Decomposition

**Figure 4:**
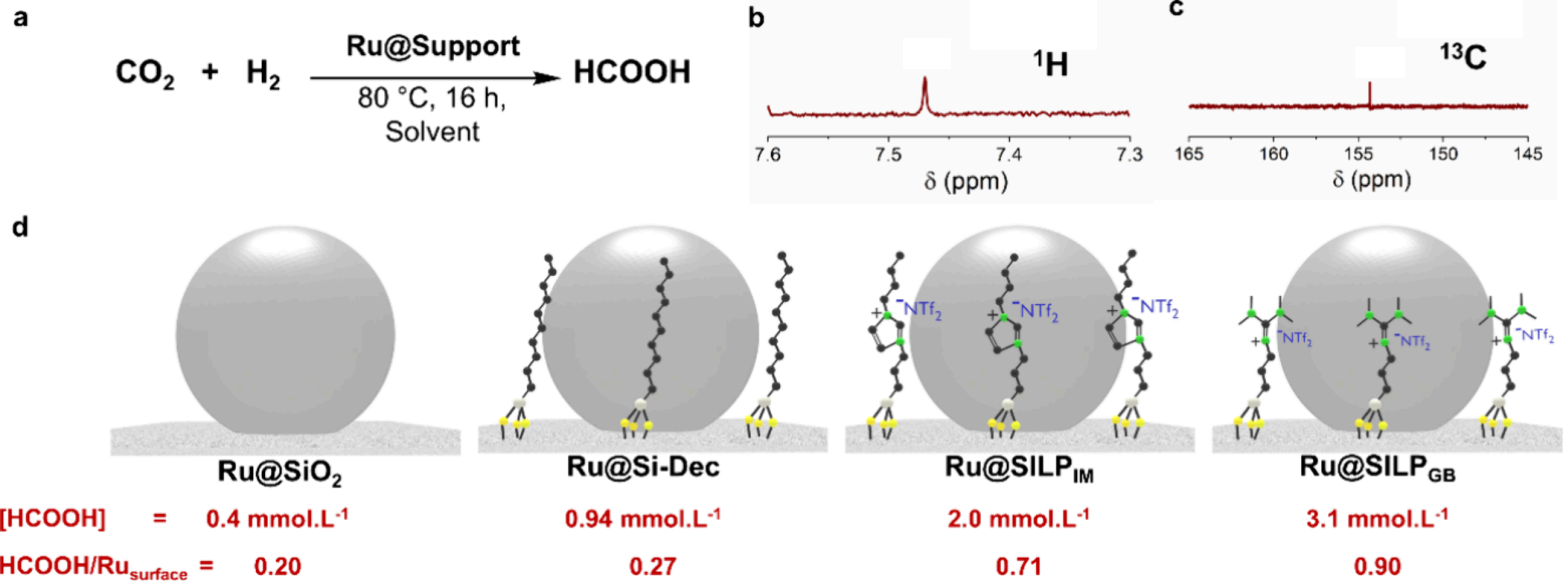
(a) Hydrogenation of CO_2_ using Ru@Support catalysts; (b) ^1^H and (c) ^13^C NMR spectra of the suspension solution after the hydrogenation of CO_2_, reaction conditions: Ru@SILP_GB_ (20 mg, 0.007 mmol), 1,4-dioxane (1 mL), 80 °C, 16 h, 500 rpm, H_2_/CO_2_ (45 bar, 1:2); (d) the structure of Ru@Support catalysts: Ru@SiO_2_, Ru@Si-Dec, Ru@SILP_IM_, Ru@SILP_GB_; the corresponding concentration of HCOOH in the suspension solution and molar ratio of HCOOH/Ru_surface_, reaction conditions: Ru@Support catalyst (0.007 mmol Ru), 1,4-dioxane (1 mL), 80 °C, 16 h, 500 rpm, H_2_/CO_2_ (45 bar, 1:2), concentration of HCOOH determined by ^1^H NMR spectra using CHCl_3_ as an internal standard.

The potential adaptivity toward the presence of carbon dioxide in the feed gas relies on the capacity of Ru@SILP_GB_ to stabilize significant concentrations of formic acid upon adjustment of the equilibrium. Therefore, Ru@SILP_GB_ was used as catalyst for the hydrogenation of CO_2_ (30 bar total pressure, H_2_/CO_2_ 1/1) at 80 °C for 16 h, using deuterated tetrahydrofuran (THF-d8) as a solvent (Figure 4a). The amount of formic acid formed was quantified by ^1^H NMR using chloroform as a standard (see SI for detailed calculation). Under these conditions, a formic acid concentration in solution of 0.4 mmol·L^–1^ was detected (Table S6). While replacing THF-d8 by 1-butanol did not have a significant impact ([HCOOH] = 0.5 mmol·L^–1^), the use of 1,4-dioxane led to a 3-fold increase in the formic acid concentration (1.3 mmol·L^–1^). Such pronounced solvent effect in CO_2_ hydrogenation to formic acid reflects the impact of solvent properties on the solubility, diffusion, and interaction of reactants (H_2_ and CO_2_) with the catalyst surface, and is consistent with previous findings.^59^ Further optimization of the pressure and H_2_/CO_2_ ratio (Table S6) resulted in a noticeable enhancement up to 3.1 mmol·L^–1^ (^1^H and ^13^C NMR spectra provided in Figure 4b,c), corresponding to a HCOOH/Ru_surface_ of 0.90. Thus, the following standard conditions were established for the rest of the study: 80 °C, 45 bar total pressure, H_2_/CO_2_ 1/2, 16 h, 1,4-dioxane as a solvent. Interestingly, applying the reference Ru@SiO_2_, Ru@Si-Dec, and Ru@SILP_IM_ catalysts under these conditions led to lower formic acid concentrations and HCOOH/Ru_surface_ ratios, with the trend Ru@SILP_GB_ > Ru@SILP_IM_ > Ru@Si-Dec > Ru@SiO_2_ (Figure 4d). Since Ru loading, NPs size, and BET surface area are similar on all these catalysts, the enhanced formic acid concentration observed when using Ru@SILP_GB_ can be associated with the capacity of the surface modifier to stabilize HCOOH.^46,47^ The enhanced formic acid stability around Ru@SILP_GB_ presumably originates from electrostatic interactions with the strong Y-shaped delocalization of the cationic center,^60^ as well as from the higher basicity of guanidinium-based ILs as compared to imidazolium ones.^61^

Since the rapid reversal of the trigger formation is an important aspect of the catalyst design for adaptivity, the reverse reaction of formic acid decomposition to CO_2_ and H_2_ was also investigated using the Ru@SILP_GB_. Solutions containing formic acid in the concentration formed under standard conditions were thus kept at 80 °C and 15 bar H_2_ in the absence of CO_2_ in the gas phase (see SI for detailed procedure). The formic acid was decomposed within 1 h to yield H_2_ and CO_2_ without any trace of CO as evidenced by headspace GC-TCD and FT-IR analyses (Figures S18 and S19, respectively). Similar observations were made with the reference catalysts (Figure S18). The high activity of Ru@SILP_GB_ for the production and decomposition of formic acid from CO_2_ and H_2_ confirms its potential to show adaptivity toward the presence of carbon dioxide in the feed gas.

#### 2.3.2 Adaptive Hydrogenation of Furanic Ketones Using Ru@SILP_GB_ under H_2_ and H_2_/CO_2_

**Figure 5:**
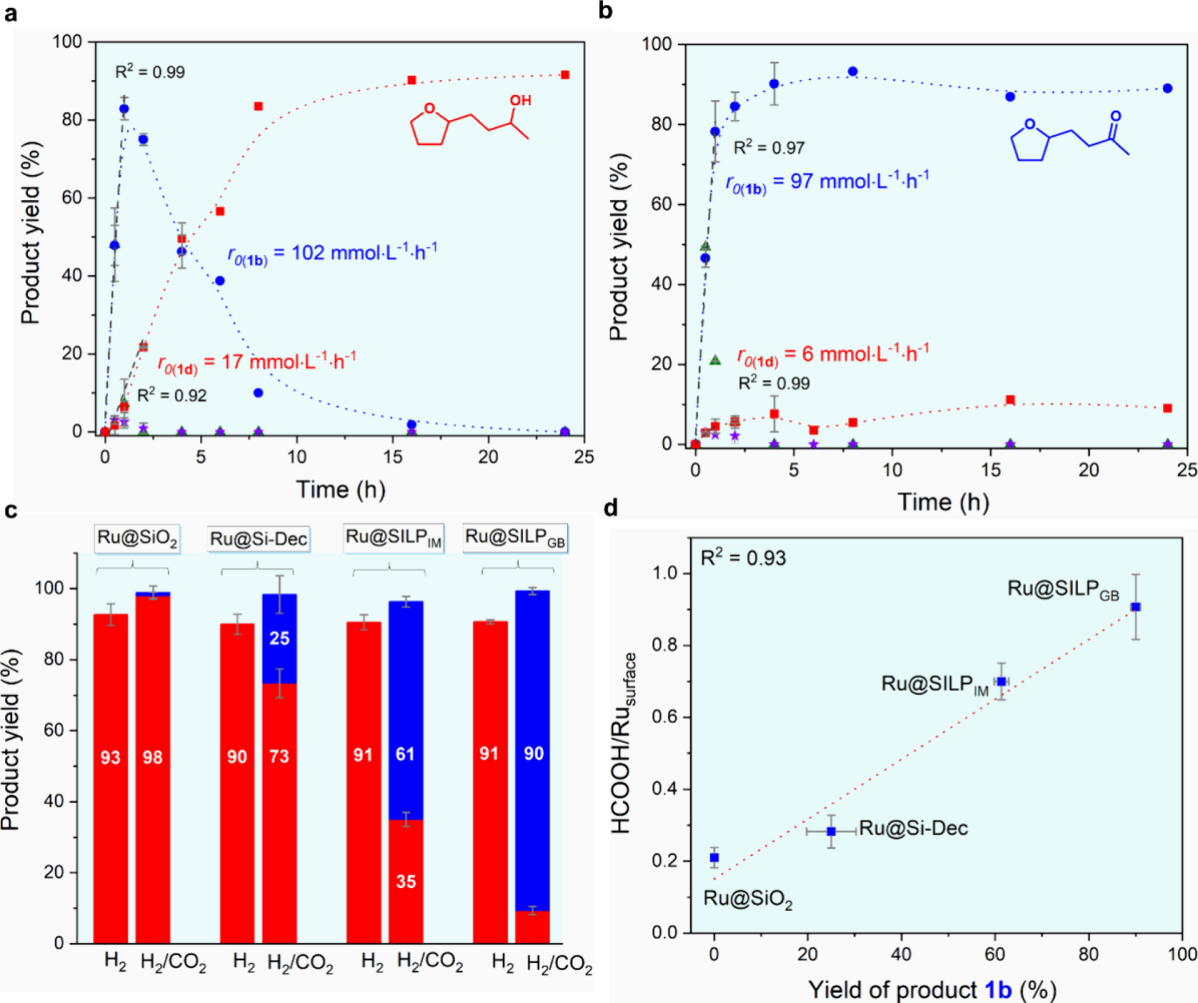
Hydrogenation of furfuralacetone (**1**) under H_2_ or H_2_/CO_2_ as feed gas. Time profiles of the hydrogenation of **1** using Ru@SILP_GB_ under (a) H_2_ and (b) H_2_/CO_2_. (c) product distribution after hydrogenation of **1** under H_2_ or H_2_/CO_2_ using Ru@Suppost catalysts. Blue bars represent product **1b** and red bars represent product **1d**. (d) Relationship of yield of product **1b** and HCOOH/Ru_surface_ ratio. Reaction conditions: Ru@Support (0.007 mmol Ru), furfuralacetone (**1**, 0.25 mmol, 35 equiv), 1,4-dioxane (1 mL), 80 °C, 500 rpm, H_2_ (15 bar) or H_2_/CO_2_ (45 bar, 1:2). Product yield determined by GC-FID using tetradecane as the internal standard. Byproduct is 2,2′-(oxybis(butane-3,1-diyl))bis(tetrahydrofuran). Data points are average values of two to four experiments and error bars represent standard deviations. Red bars: product **1d**; blue bars: product **1b**.

The hydrogenation of furfuralacetone (**1**) was selected to investigate the catalytic performance of Ru@SILP_GB_ under H_2_ and H_2_/CO_2_ as feed gases, respectively (optimization provided in Table S7), and time profiles of product formation were recorded (Figure 5a,b). Under pure H_2_, the hydrogenation of the C=C double bond and the furan ring was fast (initial rate for the formation of **1b**, *r*_0(**1b)**_ = 102 mmol L^–1^ h^–1^), followed by the hydrogenation of the ketone group with an initial rate *r*_0(**1d)**_ = 17 mmol L^–1^ h^–1^ (Figure 5a). The saturated alcohol **1d** was obtained in excellent yield (91%), which is the expected reactivity of Ru NPs under these conditions (Figure 5a).^24^

In sharp contrast, the saturated ketone **1b** was formed with high selectivity when H_2_/CO_2_ was used under otherwise identical conditions with the same partial pressure of H_2_ applied. The hydrogenation of the C=C double bond and the furan ring proceeded at a similar initial rate as under pure H_2_ (*r*_0(**1b**)_ = 97 mmol L^–1^ h^–1^), but ketone hydrogenation was essentially shut down with an initial reaction rate *r*_0(**1d**)_ = 6 mmol L^–1^ h^–1^ leading to less than 10% yield of **1d** even after 24 h (Figure 5b). The progressive decline of C=O hydrogenation activity reflects the necessary time to build-up sufficient concentrations of formic acid to suppress ketone hydrogenation activity. This was confirmed by reference catalytic experiments started directly in the presence of suitable concentrations of formic acid, and for which the initial C=O hydrogenation rate *r*_0(**1d**)_ was found much lower (1.7 mmol L^–1^ h^–1^, Figure S20).

In order to evaluate the influence of the molecular modifier on the adaptivity of the catalytic system, the performance of Ru@SILP_GB_ was compared to reference catalysts Ru@SiO_2_, Ru@Si-Dec, and Ru@SILP_IM_. The presence of CO_2_ together with H_2_ in the feed gas did not impact the performance of Ru@SiO_2_ leading to full hydrogenation to product **1d** under both gas mixtures in agreement with previous observations (Figure S21).^24^ However, mixtures of products **1b** and **1d** were obtained with the three other catalysts once CO_2_ was introduced. The selectivity toward the saturated ketone **1b** followed the order Ru@SILP_GB_ (90%) > Ru@SILP_IM_ (63%) > Ru@Si-Dec (25%, Figure 5c,d). Interestingly, this trend correlates directly with the HCOOH/Ru_surface_ ratio determined for each catalyst (Figure 5d), indicating that the C=O hydrogenation activity of Ru NPs can be effectively suppressed provided that a sufficient amount of HCOOH is adjusted by the surface molecular modifier *via* the CO_2_ hydrogenation equilibrium. This is consistent with reference experiments involving the hydrogenation of **1** under H_2_ with Ru@SiO_2_ and Ru@SILP_GB_ in the presence of various amount of formic acid as additive (Figure S22). The required starting HCOOH/Ru_surface_ ratio to suppress C=O hydrogenation is higher for Ru@SiO_2_ (11) than for Ru@SILP_GB_ (3), reflecting important differences in the catalysts’ abilities to stabilize a certain level of formic acid concentration under reaction conditions.

**Figure 6:**
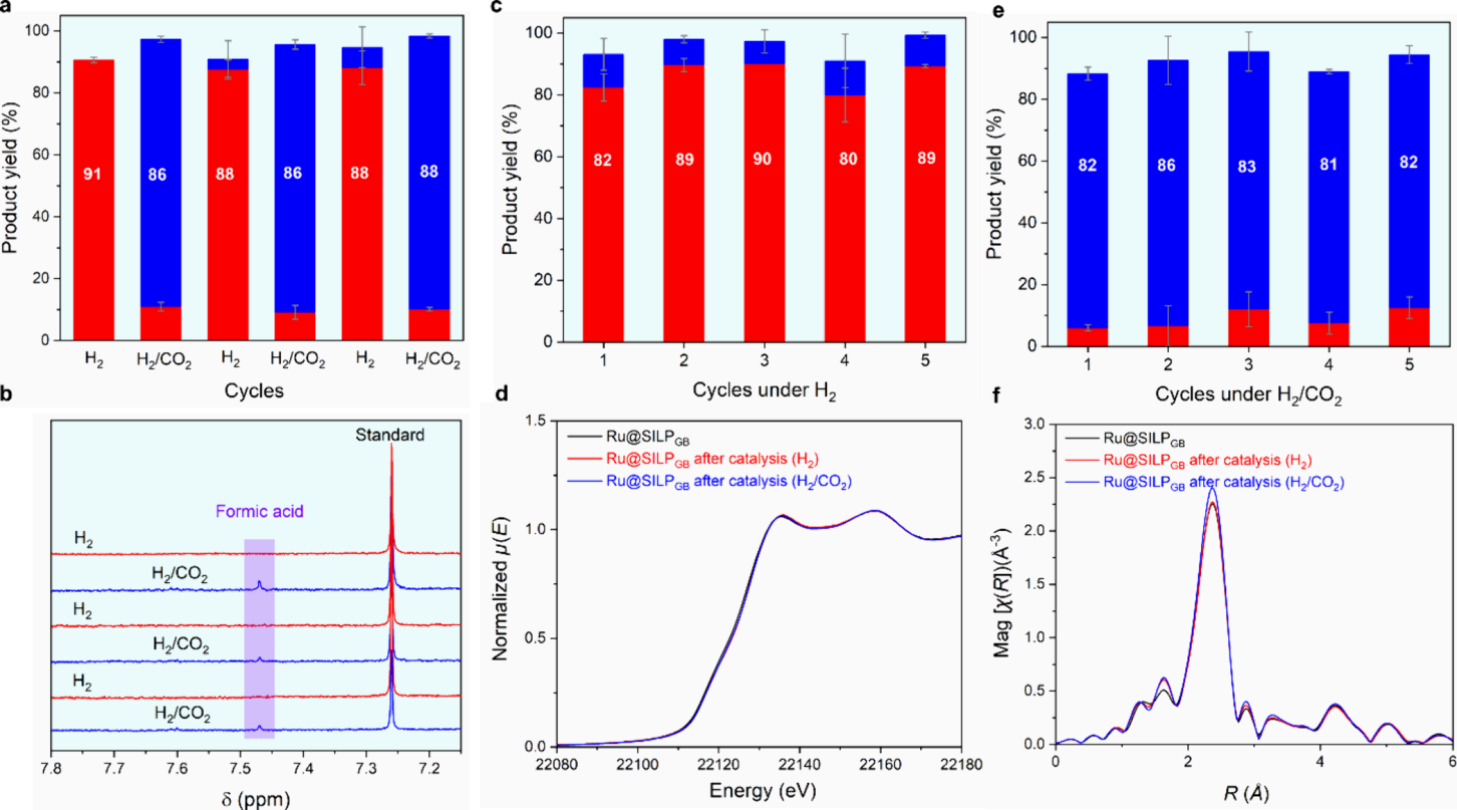
(a) Product distributions for consecutive cycles of the hydrogenation of **1** using Ru@SILP_GB_ while alternating the feed gas between H_2_ and H_2_/CO_2_ and (b) ^1^H NMR monitoring of the HCOOH content of the reaction solutions as a function of the feed gas composition. (c, e) Recycling experiments for the hydrogenation of furfuralacetone (**1**) using Ru@SILP_GB_ under (c) H_2_ and (e) H_2_/CO_2_; corresponding (d) *k*^2^-weighted R-space FT-EXAFS spectra and (f) Ru K-edge XANES spectra (normalized) plot in Magnitude without phase correction) for Ru@SILP_GB_ after reaction under H_2_ and H_2_/CO_2_. Reaction conditions: Ru@SILP_GB_ (20 mg, 0.007 mmol Ru), furfuralacetone (**1**, 0.25 mmol, 35 equiv), 1,4-dioxane (1 mL), H_2_ (15 bar) or H_2_/CO_2_ (45 bar, 1:2), 80 °C, 16 h for (a, b), 2 h for (c,e). Product yield determined by GC-FID using tetradecane as the internal standard. Conversion >99% and the byproduct is 2,2′-(oxybis(butane-3,1-diyl))bis(tetrahydrofuran). Data points are average values of three experiments and error bars represent standard deviations. Red bars: product **1d**; blue bars: product **1b**.

Satisfyingly, the selectivity switch was found fully ***reversible***, and alternating between H_2_ or H_2_/CO_2_ as feed gas allowed producing product **1d** (86–88%) or product **1b** (88–91%) in high yields and selectivity in six consecutive cycles without catalyst regeneration (Figure 6a). The direct correlation of the selectivity switch with the reversible formation of HCOOH was demonstrated by concomitant ^1^H NMR monitoring of the reaction mixture under H_2_ and H_2_/CO_2_ (Figure 6b). Importantly, no workup (e.g., heat treatment or washing step, Figures 6a,b and S23) was necessary between the cycles to regenerate ***rapidly*** the activity of pristine Ru NPs, demonstrating the real time reversibility of the selectivity switch as a noticeable improvement over previously reported Ru@PGS systems.^24,26^ The ***robustness*** of Ru@SILP_GB_ was further investigated through recycling experiments under H_2_ and H_2_/CO_2_. The conditions were slightly modified to obtain product mixtures to probe changes in performance directly. Five consecutive cycles were performed with H_2_ (Figure 6c) and with H_2_/CO_2_ (Figure 6e). Product distributions remained constant within experimental error under both sets of conditions without obvious signs of deactivation.

Independent of the used feed gas, T_2_ and T_3_ signals were unchanged in the solid-state ^29^Si CP-MAS NMR analysis of Ru@SILP_GB_ after catalysis indicating the presence of the chemisorbed IL-like molecular modifier (Figure S24). BET surface areas (Table S8) increased slightly to 330 m^2^·g^–1^ after reaction under H_2_ and 354 m^2^·g^–1^ after reaction under H_2_/CO_2_ as compared to that of fresh catalyst (302 m^2^·g^–1^). This results presumably from loss of small quantities of the guanidinium modifier physisorbed rather than chemisorbed to the SiO_2_ surface during preparation. Ru loadings after 5 cycles under H_2_ or H_2_/CO_2_ were determined to 3.7 and 4.0 wt %, respectively (Table S8). The slight increase relative to the fresh catalyst (3.5 wt %) also reflects probably the removal of the nonchemisorbed modifier. The heterogeneous nature of the catalysis was confirmed as the Ru content in the reaction solution was below 2 ppm (Table S9) and the reaction solution after hot filtration of the catalyst did not show any activity under both H_2_ and H_2_/CO_2_ (Figure S25). The used Ru@SILP_GB_ catalysts exhibited slight aggregation of Ru NPs as compared to the fresh catalyst, again independently of the used gas composition (Figure S26). XANES and EXAFS spectra of Ru@SILP_GB_ after catalysis were nearly identical to the fresh Ru@SILP_GB_, indicating good Ru NPs stability as no noticeable change in oxidation state nor coordination structure were observed (Figure 6d,f). These results demonstrate the robustness of the Ru@SILP_GB_ catalyst and the absence of irreversible structural or electronic modifications arising from its use under different feed gases.

The adaptivity of Ru@SILP_GB_ was further explored by expanding the substrate scope to a variety of ketone-containing furan derivatives. Satisfyingly, the hydrogenation selectivity could be controlled using CO_2_ as the molecular trigger for these substrates as well under optimized conditions, yielding either the saturated alcohol or ketone as products in high yields (optimization steps in Table S10). Together with the previous findings,^24^ this further substantiates the generalization of the concept for selective production of different hydrogenation products under H_2_ or under H_2_/CO_2_ with introduction of carbon dioxide into the feed gas as the only change in conditions (Table 2).

**Table 2:**
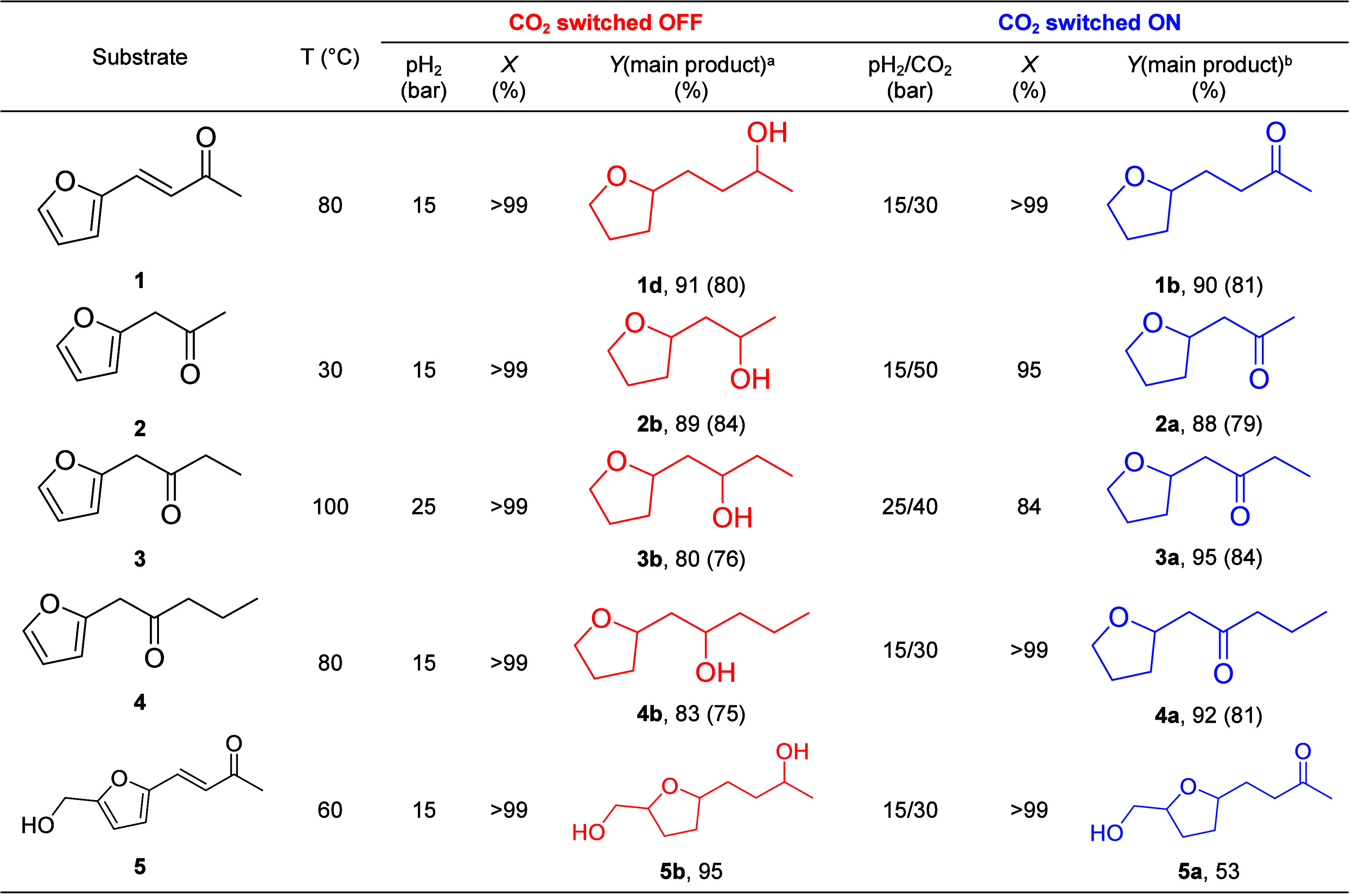
Hydrogenation of Ketone Derivatives using Ru@SILP_GB_ under H_2_ or H_2_/CO2^a^

a Reaction conditions: Ru@SILP_GB_ (20 mg, 0.007 mmol Ru), substrate (0.25 mmol, 35 equiv), 1,4-dioxane (1 mL), 16 h, X = conversion, Y = yield. Conversions and product yields determined by GC-FID using tetradecane as the internal standard.

b Rest = corresponding byproduct formed by the dehydration of saturated alcohol.

c Rest = saturated alcohol products. Isolated yields are given in parentheses and the NMR spectra of isolated products are provided in Figures S28–S43.

## 3 Conclusions

In conclusion, the adaptive control of ketone hydrogenation using Ru-based catalysts employing H_2_ or H_2_/CO_2_ feed gas was elucidated by mechanistic studies supported by DFT calculations, revealing formate coverage at the Ru surface as the key control factor for the reversible selectivity switch. The fundamental understanding served as basis for the development of a new adaptive catalytic system exploiting the reversible hydrogenation of CO_2_ to formic acid as a trigger to control product formation upon hydrogenation of furanic ketones. In particular, Ru NPs were immobilized on a new guanidinium-based supported ionic liquid phase (SILP_GB_). The resulting Ru@SILP_GB_ was characterized in pristine form as well as after exposure to catalytic relevant conditions using various techniques including N_2_ physisorption, electron microscopy, solid state NMR, pair distribution function analysis, and X-ray absorption spectroscopy. The hydrogenation of biomass-derived furfuralacetone and related ketone substrates confirmed the practically instantaneous on/off switching of the C=O hydrogenation with Ru@SILP_GB_ under H_2_ or H_2_/CO_2_. The guanidinium-based surface molecular modifier was found essential to stabilize concentrations of formic acid sufficient to observe a selectivity switch (HCOOH/Ru_surface_ ratio close to 1), which was not observed using imidazolium-based Ru@SILP_IM_, C10-chain modified Ru@Si-Dec, and Ru@SiO_2_ catalysts. The selectivity switch was found fully reversible, rapid, and robust, providing either full hydrogenation under H_2_ or partial hydrogenation under H_2_/CO_2_.

The elucidation of the control mechanism from formic acid/formate adsorption on metal surfaces may help to pave the way toward the development of next-generation adaptive catalytic systems exploiting more generally formic acid from CO_2_ as molecular trigger in NPs-catalyzed reactions. As evidenced in this study, the major prerequisite for catalyst design is the ability to shift the H_2_ + CO_2_  HCOOH equilibrium sufficiently to exploit a competing adsorption of surface formate and targeted functional groups. Consequently, the concept is not restricted to ruthenium nanoparticles as the active component nor to ketone vs furan hydrogenation and further studies to explore this potential seem promising.

## Supporting Information Available

Experimental details, data processing and evaluation, materials, theoretical calculations, additional data including reference catalyst characterization, IR spectra, data fitting details, spent catalyst characterization, parameter optimization, and NMR spectra (PDF)
