# Supplementary material for: Design and Understanding of Adaptive Hydrogenation Catalysts Triggered by the H2/CO2–Formic Acid Equilibrium
Source: J Am Chem Soc. 2024 Sep 25;146(44):30057–67. doi: 10.1021/jacs.4c06765 (PMC11544612; doi:10.1021/jacs.4c06765)
Supplement: Supplementary file 1 — ja4c06765_si_001.pdf [file ja4c06765_si_001.pdf]

Design and Understanding of Adaptive Hydrogenation Catalysts Triggered by the H_2/CO_2-Formic Acid Equilibrium

Yuyan Zhang,[a] Natalia Levin,[a] Liqun Kang,[a] Felix Müller,[c] Mirijam Zobel,[c] Serena DeBeer,[a] Walter Leitner,[a,b*] Alexis Bordet,[a*]

Contents

1 Safety Warning 2

2 General 2

3 Analytics 3

4 Theoretical calculations methodology 7

5 Synthesis 8

6 Catalytic study 10

7 Product analysis 13

8 Quantification of the HCOOH by NMR and determination of Ru surface atoms 15

9 Supplementary Figures 16

10 Supplementary Tables 34

11 Isolation of products 41

12 References 52

1 Safety Warning

High-pressure experiments involving compressed gases require the use of appropriate equipment and adherence to strict safety protocols. In this study, stainless steel autoclaves were used (max pressure = 200 bar; pressure-release safety valve set at 150 bar; used pressures up to 45 bar). After hydrogenation reactions, hydrogen was safely vented from the reactor in a ventilated fume hood. Carbon monoxide gas must be used only in a properly functioning gas cabinet or chemical fume hood. A CO gas detector should be continuously operated in laboratory areas where CO gas cylinders are stored or used. The detectors' proper operation should be regularly verified before use. Working with 1,4-dioxane requires proper training on its handling and storage. Store 1,4-Dioxane in a cool, dry, and well-ventilated location, away from light and oxidizing agents to prevent the formation of dangerous peroxides. Avoid sources of ignition, including smoking and open flames, in areas where 1,4-dioxane is used or stored.

2 General

If not otherwise stated, the immobilization of ruthenium nanoparticles (Ru NPs) on the supported materials (Ru@SiO_2, Ru@Si-Dec and Ru@SILP) was carried out under an inert atmosphere (Ar) using standard Schlenk techniques or inside a glovebox. Reaction mixtures were prepared under air, but were flushed with H_2 or H_2/CO_2 prior to catalysis. All other chemicals and solvents were purchased from commercial sources and used without purification, as listed below:

Chemicals	Abbreviation	Purity	Origin
3-chloropropyltriethoxysilane		95%	Sigma-Aldrich
Sodium iodide	NaI	99.999% trace metals basis	Sigma-Aldrich
Acetone		Anhydrous(<0.005% water), ≥99.8%	Thermo Scientific
1,1,3,3-tetramethylguanidine		99%	Sigma-Aldrich
Toluene		>99.8%	Carl Roth GmbH + Co. KG
Pentane		>99%	Carl Roth GmbH + Co. KG

Dichlormethan	DCM	>99.5	Carl Roth GmbH + Co. KG
Lithium bis(trifluoromethane)sulfonimide	$LiNTf_2$	>99%	Sigma-Aldrich
Bis(2-methylallyl)(1,5-cyclooctadiene)ruthenium(II)	[Ru(2-methylallyl)$_2$(cod)]	97%	ABCR GmbH
Tetrahydrofuran	THF	>99.5%	Carl Roth GmbH + Co. KG
1,4-Dioxane		≥99,8 % (≤100 ppm H_2O), stabilised	Carl Roth GmbH + Co. KG
1-Butanol		>99.5%	Carl Roth GmbH + Co. KG
Tetrahydrofuran-d8	THF-d8	≥99.5 %, contains 0.03 % (v/v) TMS	Sigma-Aldrich
Formic acid	HCOOH	≥95%	Sigma-Aldrich
Furfuralacetone		98%	ABCR GmbH
Tetradecane		≥99%	Sigma-Aldrich
Acetic acid	CH_3COOH	≥99%	Sigma-Aldrich
Chloroform	$CHCl_3$	≥99.8%	Carl Roth GmbH + Co. KG
2-Furylacetone		99%	ABCR GmbH
1-(2-Furyl)-2-butanone		95%	ABCR GmbH
1-(2-Furyl)-2-pentanone		95%	ABCR GmbH

3 Analytics

- Liquid state NMR spectra were recorded on a Bruker AV-400 spectrometer. The coupling constants (*J*) are given in Hertz (Hz), the chemical shifts (*δ*) are expressed in ppm relative to TMS at 298 K. The peak patterns are indicated as follows: *s* = singlet; *d* = doublet; *t* = triplet, *q* = quartet, *m* = multiplet.

- Solid state ^{13}C & ^{29}Si cross polarization-magic angle spinning (CP-MAS) NMR spectra were recorded on a Bruker double resonance 1.3 mm probe at 16.4 T static magnetic-field strength. The spectra were processed with the software Topspin (3.6.4 and 4.1.3,

Bruker Biospin) and calibrated relative to TMS using an external calibration on adamantane recorded in the same probe directly before the measurements.

- N_2 physisorption measurements were performed on a Quadrasorb SI (Quantachrom Instruments). Before the measurements, the samples were degassed under vacuum at 200 °C for 8 h. The specific surface area was evaluated using the BET method and adsorption data in the range of relative pressure p/p_0 = 0.05 - 0.25.

- Thermogravimetric analysis (TGA) was performed on a PerkinElmer TGA 8000 coupled with Clarus 600T MS. The samples were heated to 1000 °C with a heating rate of 50 °C/min in Ar atmosphere. All the measurements were performed by Marius Heise-Podleska.

- High-angle annular dark-field scanning transmission electron microscopy (HAADF-STEM) was performed on a Hitachi HF2000 cold FEG operating at 200 kV at the Max-Planck-Institut für Kohlenforschung. Samples were prepared by depositing the powder on a copper TEM grid with an amorphous carbon support film. High-resolution aberration-corrected BF-STEM and HAADF-STEM images were acquired using a probe-corrected (CEOS) JEOL ARM300CF electron microscope in the E02 lab of the electron Physical Science Imaging Centre (ePSIC) at Diamond Light Source (DLS, UK). The acceleration voltage for the electron gun was 300 kV. The probe size was set to 8C (Spot 8) with a 40 μm probe-forming aperture (CL aperture) selected, resulting in a probe convergence semi-angle of 33.6 mrad and a beam current of 41.4 pA. The STEM camera length was set to 9.0 cm, which allowed the ADF detector to integrate the scattered electron intensity between 77.0 ± 0.8 and 209.4 ± 10.7 mrad. In addition, a 3 mm aperture was inserted for the BF imaging, corresponding to a semi-angle of 15.3 ± 1.0 mrad (outer angle) for the BF detector. For each sample, a small amount of dry powder was sprinkled on a 400-mesh Cu grid with lacey carbon support film, and the grid was then baked inside a vacuum chamber at 60 ^{o}C for 15 minutes. Each sample was exposed to an intense electron beam for 10-15 minutes ('beam shower') to eliminate the accumulation of carbon contamination during the STEM imaging. Gatan Microscopy Suite software was used for image data acquisition. The FFT image processing was performed using the scikit-image python library (version 0.20.0).

- Total scattering data was acquired at the P02.1 beamline at DESY facility, Hamburg (Germany). Measurements were conducted for 100 seconds employing a photon energy of 59.8 keV and a Dectris Pilatus3 X CdTe 2M detector. Powder samples were packed in Kapton capillaries with a 1 mm diameter. Distance and tilt calibration were done with a LaB_6 standard (CAS: 12008-21-8). The xpdtools software was used for radial integration.[1] For the processing of data and subsequent refinement of the G(r) function, the software packages PDFgetX3 and Diffpy-CMI were employed.[2, 3] For fitting of the metallic Ru phase a hcp structure was used (database code: ICSD 235818).[4]

- Adsorption of CO for FT-IR spectroscopy was performed using the following procedure. Firstly, the catalyst (ca. 20 mg with 3.5 wt% Ru loading, corresponding to ca. 0.7 mg Ru) was placed in a Fischer-Porter bottle and evacuated under high vacuum for 15 min. Subsequently, the Fischer-Porter bottle was refilled with 4 bar CO. After 18 h, the carbon monoxide atmosphere was removed, and a high vacuum was applied for 5 min before the Fischer-Porter bottle was refilled with argon and transferred in a glovebox. There, the catalyst was recovered from the Fisher-Ported bottle and the Sandwich structure pellets using KBr (ca. 30 mg) were prepared for transmission IR measurements.[5]

-Adsorption of formic acid and acetic acid for FT-IR spectroscopy was using the following procedure. Firstly, the adsorption of acetic acid on $Ru@SiO_2$ (ca. 20 mg) was carried out at room temperature using a formic acid/Ar or acetic acid/Ar mixture for 60 min, followed by evacuated under high vacuum for 15 min at 100 °C. The resulting formic acid-adsorbed $Ru@SiO_2$ or acetic acid-adsorbed $Ru@SiO_2$ were solids, and were measured in ATR mode.

-Inductively coupled plasma optical emission spectroscopy (ICP-OES, for Ru quantification) was carried out at Mikroanalytisches Laboratorium Kolbe on a Perkin Elmer Analyst 200 Atomic Absorption Spectrometer.

-The Ru K-edge (22117 eV) X-ray Absorption Fine Structure (XAFS) of the $Ru@SILP_{GB}$ samples (fresh and spent $Ru@SILP_{GB}$ catalysts) were collected at the P65 beamline of PETRA III (P65 Applied X-ray Absorption Spectroscopy). At P65 beamline, monochromatic beam was introduced through an 11-periods undulator and a Si (311) double crystal monochromator (DCM) with energy resolution $\Delta E/E$ of 6.0×10^{-5}. The DCM

was operated in QEXAFS mode, and the undulator offset to the DCM was calibrated to have the maximum photon flux. The beam size at the sample was approx. 0.5 x 1.0 mm^2 (V x H) and the photon flux was ~10^{11} photons/s (without attenuation). The Ru *K*-edge XAFS of the reference Ru samples (Ru@$SILP_{Im}$, Ru@Si-Dec and Ru@SiO_2) were acquired at the B18 beamline of Diamond Light Source (B18 Core XAS). [6, 7] The B18 beamline is equipped with a bending magnet source and a Si(311) DCM (energy resolution $\Delta E/E = 3 \times 10^{-5}$) operated in QEXAFS mode to produce monochromatic X-ray beam. The photon flux on the sample is similar to that at P65 and the beam size is slightly bigger (approx. 1.0 x 1.0 mm^2). The XAFS spectra for all samples at each beamline were collected in transmission mode, and the intensity of incident beam (I_0) and the transmitted beam (I_t) was monitored by ionization chambers (filled with mixture of Kr and N_2). The energy ranges for the full XAFS spectra collected at P65 beamline and B18 beamline were 21917-23417 eV (k_{max} = 18.2) and 21917-23100 eV (k_{max} = 16), with the corresponding energy step sizes of 0.6 eV and 0.3 eV, respectively. The XAFS of each sample was measured 3 times and merged to improve the signal-noise ratio. Ru foil was measured simultaneously for each sample as the reference for energy calibration. The energy of the incident beam was calibrated by assigning the energy of the first inflection in the first derivative XANES of Ru foil to 22117 eV. In addition to Ru foil, commercial RuO_2 powder was also measured in transmission mode as a reference. The Ru K-edge XAS spectra were analyzed using the Demeter software package (including Athena and Artemis programs, version 0.9.26).[8] Pre-edge background subtraction and post-edge normalization of the XAFS data were performed using the Athena program. A linear regression background in the range of 21917 to 22027 eV) was determined, and a quadratic polynomial regression for post-edge normalization in the range of 22277 to 23100 eV was applied. The spectra were splined from k=0 to 16.5 $Å^{-1}$ with rbkg of 1.0 Å and *k*-weight of 2. The fitting of EXAFS spectra (R range: 1 to 3 Å, *k*-range: 3.7 to 13.5 $Å^{-1}$) was performed using the Artemis program based on scattering paths generated from FEFF6. The amplitude reduction factor S_0^2 is determined to be 0.70 by fitting of k^2-weighted R-space EXAFS of Ru foil based on the standard crystal parameters of Ruthenium metal (data from Crystal Open Database, entry ID: 9008513), and was used as fixed parameter for the EXAFS fitting of other Ru samples.

4 Theoretical calculations methodology

All quantum chemical calculations employed density functional theory (DFT) and were performed with Quantum Espresso 6.8[9-11] using the plane-wave PWscf code. The projector augmented wave (PAW) type of Perdew-Burke-Ernzenhof (PBE) pseudopotentials were used.[12, 13] Through convergence tests, the kinetic energy cutoff for wave functions was set at 67 Ry, the kinetic energy cutoff for charge density was set at 453 Ry, a degauss smearing of 0.05 Ry was applied and a Marzari-Vanderbilt[14] smearing was selected. Given that DFT often fails to represent dispersion interactions properly,[15] a correction based on the semiempirical Grimme's DFT-D3 was employed.[16]

For the calculations, a slab model of Ru was built based on the optimized geometry of the bulk material. The lattice plane Ru(0001) was selected as the surface plane since it is thermodynamically stable and believed to be more reactive than other planes.[17, 18] A 4 × 4 supercell with 4 layers was used to describe the slab, of which the bottom two were frozen to their bulk positions and the upper two were relaxed during optimizations. A separation of 15 Å of vacuum was used to prevent spurious interactions between the periodic slabs. The Brillouin zone was sampled for the slab geometries with a Monkhorst-Pack 6 × 6 × 1 mesh. Several initial geometries for the adsorption of each compound were tested in order to obtain representative information of possible different adsorption modes. The adsorption energies of each molecule were calculated according to the next equation:

$$E_{ads} = E_{slab+adsorbate} - E_{free\ slab} - E_{free\ adsorbate} \quad \text{(Eq. 1)}$$

To investigate HCOOH adsorption, the proton of the HCOO-H molecule was placed in a perturbed starting positing and left to relax. Starting from different configurations led to the same final converged adsorption mode with the two O interacting with the Ru surface through a bridge configuration and the dissociated H stabilized in an hcp hollow site. Although the calculation specifies the charge of the complete dissociated formic acid, which is neutral, it does not describe explicitly the charges of the individual fragments.

5 Synthesis

5.1. Synthesis of 3-iodopropyltriethoxysilane:

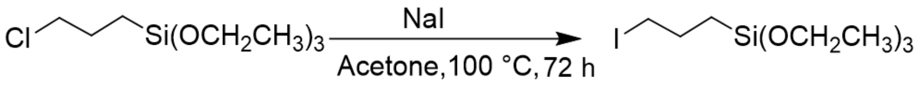

3-chloropropyltriethoxysilane (100 mL, 1 eq.) was added to a solution of NaI (65.4 g, 1.05 eq.) in anhydrous acetone (150 mL) and was stirred for 72 h under Ar at 100 °C in a reflux set up. After 72 h, the reaction mixture was cooled down to room temperature, and the slurry obtained was distilled under vacuum at 150 °C to yield 3-iodopropyltriethoxysilane as pale-yellow oil (126.3 g, 380 mmol, 87%).[19]

^{1}H NMR (400 MHz, Chloroform-*d*): δ (ppm) = 3.75 (q, *J* = 7.0 Hz, 6H), 3.15 (t, *J* = 7.0 Hz, 2H), 1.91-1.81(m, 2H), 1.16 (t, *J* = 7.0 Hz, 9H), 0.71 - 0.58 (m, 2H).

5.2. Synthesis of 1,1,3,3-tetramethyl-2-[3-(trimethoxysilyl)propyl] guanidium bis(trifluoromethylsulfonyl)imide (IL$_{GB}$)

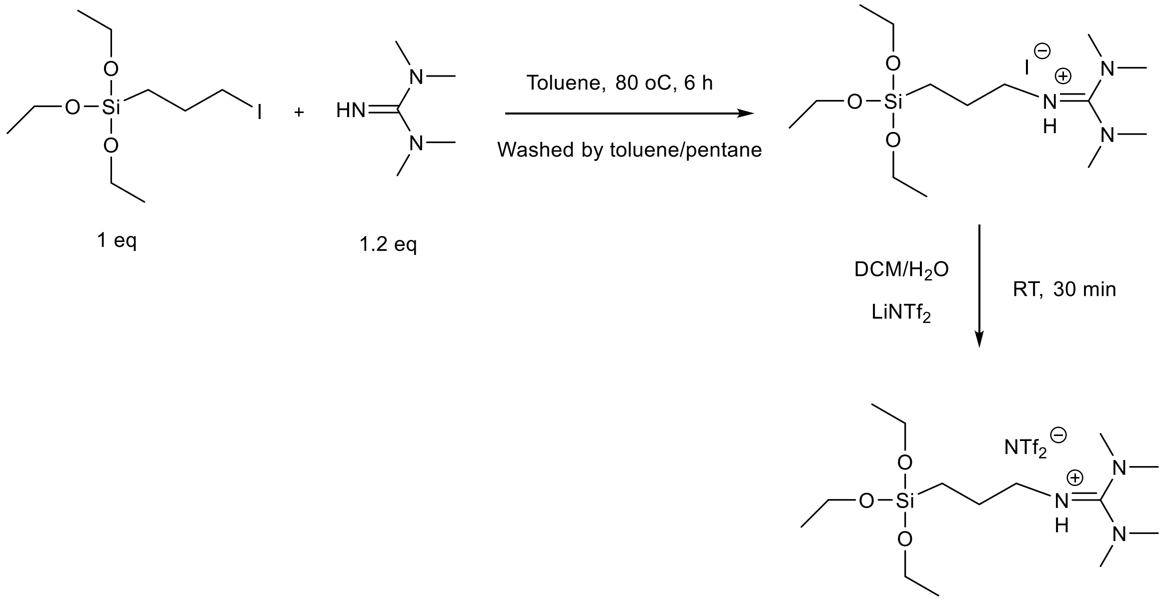

1,1,3,3-tetramethylguanidine (1.38 g, 12 mmol, 1.2 eq.) was weighed in a Schlenk flask in the glovebox and dried under vacuum prior to use, then dissolved in 60 mL anhydrous toluene. 3-iodopropyltriethoxysilane (3.3 g, 10 mmol, 1.0 eq.) was weighed separately into a Schlenk flask in the glovebox and added dropwise to the 1,1,3,3-tetramethylguanidine solution. The mixture was refluxed for 18 h at 130 °C under Ar. After

cooling down the reaction, the obtained mixture was washed 3 times with toluene/pentane (10 mL/10 mL) and then dried under vacuum at RT for 15 h to form a viscous orange oil (80 % yield).[20]

For the anion exchange, 1,1,3,3-tetramethyl-2-[3-(trimethoxysilyl)propyl] guanidium iodate (12.1 g, 27 mmol, 1.0 eq.) was dissolved in 50 mL DCM.[21] In a separate flask, lithium bis(trifluoromethane)sulfonimide ($LiNTf_2$) (8.8 g, 29.7 mmol, 1.1 eq.) was dissolved in 25 mL water. The solutions were mixed and vigorously stirred for 90 min at RT. The organic phase was washed three times with water and the solvent was removed under reduced pressure. The resulting viscous liquid was dried overnight at 60 °C in vacuo. After drying, a thick yellow/brown oil was obtained (66% yield).

^{1}H NMR (400 MHz, Chloroform-*d*): δ (ppm) = 6.15 (d, *J* = 21.6 Hz, 1H), 3.75 (qd, *J* = 7.0, 1.4 Hz, 6H), 3.14 - 3.09 (m, 2H), 2.96 - 2.90 (m, 12H), 1.67 (p, *J* = 7.6 Hz, 2H), 1.15 (t, *J* = 7.0 Hz, 9H), 0.58 - 0.48 (m, 2H).

The observed splitting of the NMe groups protons is due to a restricted rotation around the C-N bonds, consistent with literature reports.[31]

^{13}C NMR (101 MHz, Chloroform-*d*): δ (ppm) = 162.80 (s, 1C), 58.46 (s, 3C), 47.54 (s, 1C), 40.31 (s, 4C), 26.50 (s, 1C), 18.29 (s, 3C), 8.04 (s, 1C).

NMR spectra and their assignments can be found in Figure S5 and S6.

5.3. Synthesis of $SILP_{GB}$

$SILP_{GB}$ was prepared by mixing a solution of (1,1,3,3-tetramethyl-2-[3-(trimethoxysilyl)propyl] guanidium bis(trifluoromethylsulfonyl)imide) (IL_{GB} 8.8 mmol, 5.29 g in 20 mL anhydrous toluene) with a suspension of dehydroxylated (500 °C, high vacuum for 16 h) silica (10 g in 50 mL toluene). The resulting mixture was refluxed at 130 °C for 48 h under Ar. After carefully removing the solvent by decantation, the $SILP_{GB}$ was washed 3 times with toluene (15 mL, anhydrous) and dried under vacuum at 60 °C for 8 h (13.2 g $SILP_{GB}$). The organic phases were combined and evaporated to determine the amount of IL_{GB} that was not grafted on silica (recovered IL_{GB} 3.0 mmol 1.78 g)). (Total IL_{GB} grafted (5.8 mmol, 3.51 g) = starting amount of IL_{GB} (8.8 mmol, 5.29 g) – recovered IL_{GB} (3.0 mmol, 1.78 g).

Si-Dec and $SILP_{IM}$ were prepared following reported procedures.[21-24]

5.4. Synthesis of Ru@$SILP_{GB}$

[Ru(2-methylallyl)$_2$(cod)] (120 mg, 0.376 mmol) was dissolved in THF (5 mL) and added to a suspension of $SILP_{GB}$ (0.75 g) in THF (5 mL). After solvent removal, the dried impregnated $SILP_{GB}$ powder was loaded into a 10 mL high pressure autoclave and subjected to an atmosphere of H_2 (25 bar) at 100 °C for 18 h. Under this reducing environment, the impregnated $SILP_{GB}$ transformed from a white to a black color indicating the immobilization of the Ru NPs onto the $SILP_{GB}$.

5.5. Synthesis of Ru@SiO_2, Ru@Si-Dec and Ru@$SILP_{IM}$

[Ru(2-methylallyl)$_2$(cod)] (120 mg, 0.376 mmol) was dissolved in THF (5 mL) and added to a suspension of dehydroxylated SiO_2 (0.75 g) in THF (5 mL). After solvent removal, the dried impregnated SiO_2 powder was loaded into a 10 mL high pressure autoclave and subjected to an atmosphere of H_2 (25 bar) at 100 °C for 18 h. Under this reducing environment, the impregnated dehydroxylated SiO_2 transformed from a white to a black color indicating the immobilization of the Ru NPs onto the SiO_2.

Ru@Si-Dec and Ru@$SILP_{IM}$ were prepared following the same procedure.

6 Catalytic study

6.1 Hydrogenation of CO_2

In a typical experiment, Ru@$SILP_{GB}$, Ru@$SILP_{IM}$, Ru@Si-Dec or Ru@SiO_2 (0.007 mmol Ru) and solvent (1 mL) were charged in a glass insert and placed in a stainless-steel high-pressure autoclave (volume: 10 mL) equipped with a magnetic stir bar. The autoclave was sealed, purged, and pressurized with CO_2 and H_2. Then the reaction mixture was stirred at 80 °C in an aluminum heating block. Once the reaction was finished, the reactor was cooled in an ice bath and carefully vented. The suspension solution was transferred into an NMR tube and analyzed by ^{1}H and ^{13}C NMR spectroscopy using $CHCl_3$ as standard.

6.2 Decomposition of HCOOH

In a typical experiment, Ru@$SILP_{GB}$ (20 mg, 0.007 mmol Ru), 1,4-dioxane (1 mL), and formic acid were charged in a glass insert and placed in a high-pressure autoclave. The autoclave was sealed, purged, and pressurized with 15 bar H_2. The reaction mixture was

stirred at 80 °C in an aluminum heating block. Once the reaction was finished, the reactor was cooled in an ice bath and analyzed by headspace GC-FID/TCD and ^{1}H NMR using $CHCl_3$ as standard for formic acid quantification.

*6.3 Hydrogenation of furfuralacetone (**1**) with H_2 as feed gas*

In a typical experiment, the Ru@Support catalysts (0.007 mol Ru), 1,4-dioxane (1 mL), and **1** (0.25 mmol, 35 eq.) were charged in a glass insert and placed in a high-pressure autoclave. The autoclave was sealed. The reaction mixture was stirred at 80 °C in an aluminum heating block. Once the reaction was finished, the reactor was cooled in an ice bath and carefully vented. After filtration, a sample of the reaction mixture was taken and analyzed via GC-FID using tetradecane as the internal standard.

*6.4 Hydrogenation of furfuralacetone (**1**) with H_2/CO_2 as feed gas*

In a typical experiment, the Ru@Support catalysts (0.007 mol Ru), 1,4-dioxane (1 mL), and **1** (0.25 mmol, 35 eq.) were charged in a glass insert and placed in a high-pressure autoclave. The autoclave was sealed, purged, and pressurized with CO_2 and H_2. The reaction mixture was stirred at 80 °C in an aluminum heating block. Once the reaction was finished, the reactor was cooled in an ice bath and carefully vented. After filtration, a sample of the reaction mixture was taken and analyzed via GC-FID using tetradecane as the internal standard and ^{1}H NMR using $CHCl_3$ as standard for formic acid quantification.

*6.5 Time profile of hydrogenation of furfuralacetone (**1**) with H_2 or H_2/CO_2*

The time profile of hydrogenation of **1** was recorded by loading Ru@SILP$_{GB}$ (20 mg, 0.007 mmol Ru), **1** (0.25 mmol, 35 eq.), 1,4-dioxane (2 mL) into 10 glass inserts placed in high-pressure autoclaves. The mixture was diluted two times as compared to standard conditions to slow down the reaction and facilitate the collection of kinetically-relevant data. The autoclave was sealed, purged, and pressurized with the desired feed gas. The reaction mixtures were stirred at 80 °C in aluminum heating blocks. Once the reactions were finished after 0.5 h, 1 h, 2 h, 3 h, 4 h, 5 h, 6 h, 7 h, 8 h and 24 h, the reactors were cooled in an ice bath and carefully vented. After filtration, the samples of the reaction mixture were taken and analyzed via GC-FID using tetradecane as the internal standard.

6.6 Reversibility experiments

In a typical experiment, Ru@$SILP_{GB}$ (20 mg, 0.007 mmol Ru), 1,4-dioxane (1 mL) and **1** (0.25 mmol, 35 eq.) were loaded in a glass insert and placed in a high-pressure autoclave. The autoclave was sealed, purged, and pressurized with the desired feed gas. The reaction mixture was stirred at 80 °C in an aluminum heating block. Once the reaction was finished, the reactor was cooled in an ice bath and carefully vented. The mixture was centrifuged and a sample of the solution was taken and analyzed via NMR using $CHCl_3$ as standard and GC-FID using tetradecane as the internal standard. The catalyst was washed with 1,4-dioxane (3 x 1 mL), and re-used in the following cycle. For the next, fresh portions of substrate (0.25 mmol, 35 eq.) and 1,4-dioxane (1 mL) were added and the reaction was performed again. This procedure was repeated for each catalytic cycle by alternatively pressurizing the autoclave either with only H_2 or with CO_2 and H_2.

6.7 Recycling experiments under H_2

In a typical experiment, Ru@$SILP_{GB}$ (20 mg, 0.007 mmol Ru), 1,4-dioxane (1 mL) and **1** (0.25 mmol, 35 eq.) were loaded in a glass insert and placed in a high-pressure autoclave. The autoclave was sealed, purged, and pressurized with 15 bar H_2. The reaction mixture was stirred at 80 °C in an aluminum heating block. Once the reaction was finished, the reactor was cooled in an ice bath and carefully vented. The mixture was centrifuged and a sample of the solution was taken and analyzed via GC-FID using tetradecane as the internal standard. The catalyst was washed with 1,4-dioxane (3 x 1 mL). For the next cycle, fresh portions of substrate (0.25 mmol, 35 eq.) and 1,4-dioxane (1 mL) were added and the reaction mixture was performed again. This procedure was repeated for each catalyst cycle by pressurizing the autoclave with 15 bar H_2.

6.8 Recycling experiments under H_2/CO_2

In a typical experiment, Ru@$SILP_{GB}$ (20 mg, 0.007 mmol Ru), 1,4-dioxane (1 mL) and **1** (0.25 mmol, 35 eq.) were loaded in a glass insert and placed in a high-pressure autoclave. The autoclave was sealed, purged, and pressurized with a mixture of H_2/CO_2 (45 bar total pressure, 1:2 ratio). The reaction mixture was stirred at 80 °C in an aluminum heating block. Once the reaction was finished, the reactor was cooled in an ice bath and carefully vented. The mixture was centrifuged and a sample of the solution was taken and analyzed

via GC-FID using tetradecane as the internal standard. The catalyst was washed with 1,4-dioxane (3 x 1 mL). For the next cycle, fresh portions of the substrate (0.25 mmol, 35 eq.) and 1,4-dioxane (1 mL) were added and the reaction mixture was performed again. This procedure was repeated for each catalyst cycle by pressurizing the autoclave with a mixture of H_2/CO_2 (45 bar total pressure, 1:2 ratio).

6.9 Hydrogenation of other ketone derivatives with H_2 or H_2/CO_2

In a typical experiment, Ru@SILP$_{GB}$ (20 mg, 0.007 mmol Ru), 1,4-dioxane (1 mL) and ketone derivatives (0.25 mmol, 35 eq.) were loaded in a glass insert and placed in a high-pressure autoclave. The autoclave was sealed, purged, and pressurized with the desired feed gas. The reaction mixture was stirred at the desired temperature in an aluminum heating block. Once the reaction was finished, the reactor was cooled in an ice bath and carefully vented. After filtration, a sample of the reaction mixture was taken and analyzed via GC-FID using tetradecane as the internal standard.

6.10 Concentration of acetic acid or formic acid additives under room temperature

In a typical experiment, Ru@SILP$_{GB}$ (20 mg, 0.007 mmol Ru), 1,4-dioxane (1 mL), and acetic acid or formic acid (0.019 mmol) were charged in a glass vial. The reaction mixture was stirred at room temperature. Once the reaction was finished, the solution was analyzed by ^{1}H NMR using $CHCl_3$ as standard for acetic acid or formic acid quantification.

6.11 Concentration of acetic acid or formic acid additives under reaction conditions

In a typical experiment, Ru@SILP$_{GB}$ (20 mg, 0.007 mmol Ru), 1,4-dioxane (1 mL), and acetic acid or formic acid (0.019 mmol) were charged in a glass insert and placed in a high-pressure autoclave. The autoclave was sealed, purged, and pressurized with 15 bar H_2. The reaction mixture was stirred at 80 °C in an aluminum heating block. Once the reaction was finished, the reactor was cooled in an ice bath and analyzed by ^{1}H NMR using $CHCl_3$ as standard for acetic acid or formic acid quantification.

7 Product analysis

Product analysis was done by GC-FID (gas chromatography coupled with flame ionization detector) on a Shimadzu GC 2030 (see details in the Tables below), as well as by GC-MS (gas chromatography coupled with a mass spectrometer) on a Shimadzu QP 2020

instrument. The identification of the compounds was done by injecting the pure compounds in GC-FID, or by GC-MS. Product quantification was done by referencing the product peak area to the peak area of the added tetradecane standard, following internal GC calibration. The following conditions and equipment settings have been used for the liquid products analysis from the hydrogenation of **1**,**2**,**3**,**4**,**5** (or related substrates):

Stationary Phase (Column) from Agilent	CP-WAX 52 CB (0.25 µm, 0.25 mm, 60 m)
Flow Control Mode Linear Velocity	50.0 cm/sec
Injection Volume	0.3 µL
Injector Temperature	200 °C
Split Ratio	30
Temperature Program	50 °C for 5 min, to 200 °C with 8 °C/min for 10 min
Detector Temperature	200°C

The quantification of product mixtures was achieved by GC-FID using tetradecane as an internal standard. Prior to routine analysis, calibration curves were built for each substrate using the pure compounds. Since the calibration factors of each substrate and corresponding products is similar based on the Effective Carbon Number (ECN) approach,[25] here we used the same calibration factors of each substrate and corresponding products. Correction factors are as follows:

- **1** and related products: *k* = 1.58
- **2** and related products: *k* = 1.87
- **3** and related products: *k* = 2.29
- **4** and related products: *k* = 2.13
- **5** and related products: *k* = 2.40

The quantification of reactants and products were based on the following equation:

$$\frac{n_s}{A_s} = k * \frac{n_x}{A_x}$$

Where n_s = the mole of tetradecane, A_s = the GC area of tetradecane, n_x = the mol of each reactant/product, A_x = the GC area of each reactant/product."

Gas phase analysis was done by headspace GC-FID/TCD (gas chromatography coupled with FID and TCD-detectors) on a Shimadzu GC 2030 Nexis equipped with two columns installed with a Y-connection at the injector side.

Stationary Phase (Column) from Agilent	Rt-Q-Bond by Restek (FID)	Carboxen 1010 PLOT by Supelco (TCD)
Injector Temperature	250 °C	250
Split Ratio	5	5
Temperature Program	45°C for 5 min, to 250°C with 15°C/min, 260°C for 10 min	45°C for 5 min, to 250°C with 15°C/min, 260°C for 10 min
Detector Temperature	250°C	260°C

8 Quantification of HCOOH by ^{1}H NMR and determination of Ru surface atoms

The concentration of formic acid ($c_{HCOOH,}$ mmol/L) and acetic acid ($c_{\text{acetic acid,}}$ mmol/L) were quantified using the following formula:

$$c_{HCOOH} = \frac{m_{std}}{M_{std}} \times \frac{N_{std}}{N_{HCOOH}} \times \frac{S_{HCOOH}}{S_{std}} / V \quad (1)$$

$$c_{acetic\ acid} = \frac{m_{std}}{M_{std}} \times \frac{3 \times N_{std}}{N_{acetic\ acid}} \times \frac{S_{acetic\ acid}}{S_{std}} / V \quad (2)$$

S = Integrated area of the peak, *N* = number of protons generating the selected signals for integration, *m* = mass (mg), *M* = Molecular weight (g·mol^{-1}), *V* = the volume of reaction mixture (L).

^{1}H NMR measurement parameters for the quantification of formic acid were as follows: delay time of 1 s, 16 scans, a 10° pulse angle, and a sample temperature of 298 K.

Determination of Ru surface atoms

The %(surface Ru) were estimated for each catalyst by calculating the volume of the Ru NPs as well as the volume of the shell containing the first layer of Ru atoms

Volume of nanoparticles:

$$V_{NPs} = \frac{4}{3}\pi \times r^3{}_{NPs}$$

Volume of the shell containing the first layer of ruthenium atoms

$$V_{NPs} = \frac{4}{3}\pi \times \left(r^{3}{}_{NPs} - \ \left(r_{NPs} - \ r_{at(Ru)}\right)^{3}\right)$$

With the atomic radius of the Ru ($r_{at(Ru)}$) = 0.13 nm.

$$\%_{Surface\ Ru} = \frac{V_{Shell}}{V_{NPs}}$$

9 Supplementary Figures

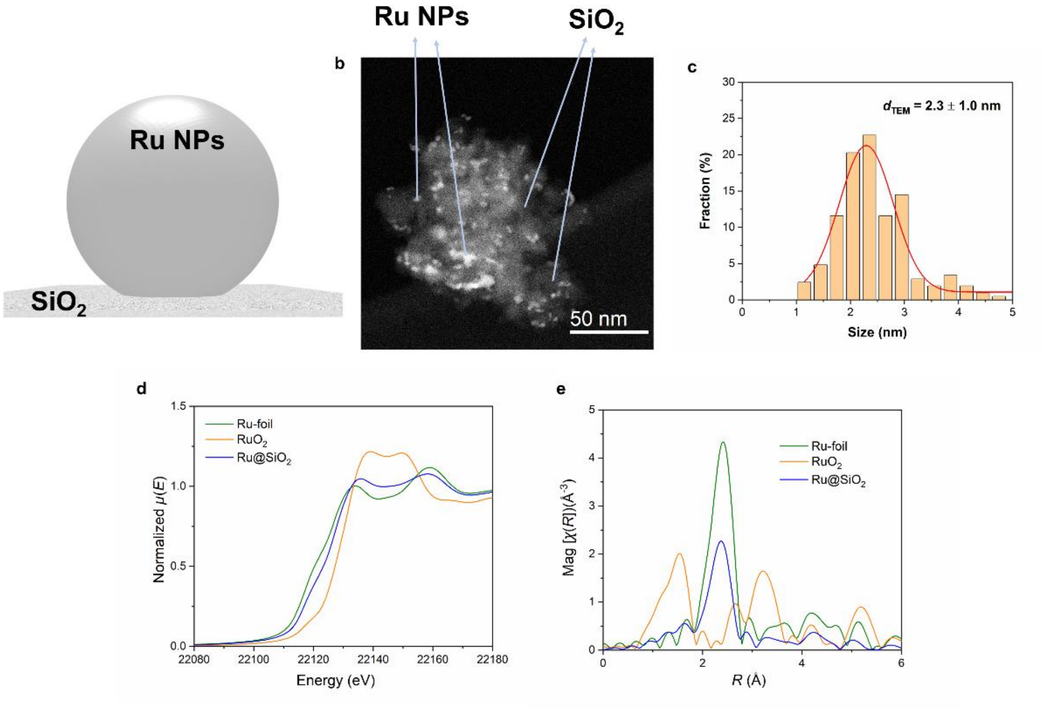

Figure S1. (a) Schematic representation of Ru@SiO_2, (b) HAADF-STEM image, indicating the Ru NPs as bright spot and dark area for support SiO_2, (c) particle size distribution on 207 Ru NPs, (d) k^2-weighted R-space FT-EXAFS spectra (plot in Magnitude without phase correction) and (e) K-edge XANES spectra (normalized) for Ru-foil, Ru@SiO_2 and RuO_2, (green, blue and orange curves, respectively).

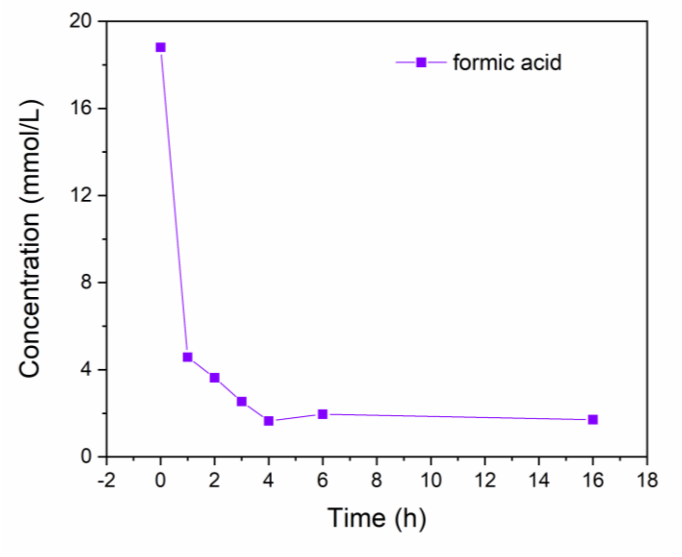

Figure S2. Change in the concentration of formic acid with time under following reaction conditions: $Ru@SiO_2$ (20 mg, 0.007 mmol Ru), 1,4-dioxane (1 mL), 80 °C, 500 rpm, H_2 (15 bar), determined by NMR spectra using $CHCl_3$ as an internal standard.

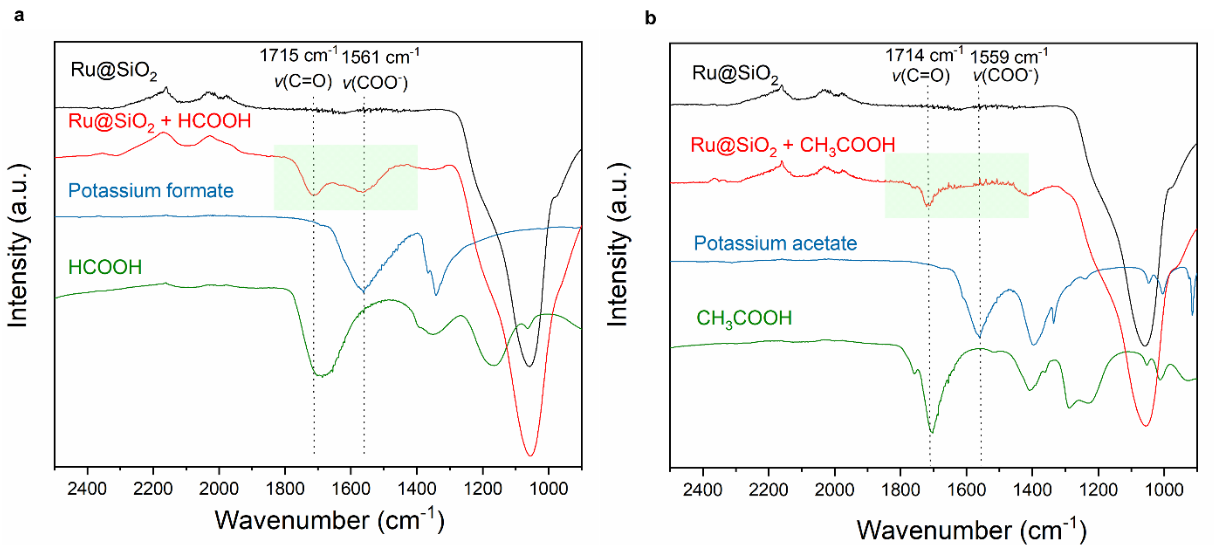

Figure S3. (a) FTIR spectrum of $Ru@SiO_2$, after adsorb formic acid on $Ru@SiO_2$, potassium formate and HCOOH measured in ATR mode, (b) FTIR spectrum of $Ru@SiO_2$, after adsorb acetic acid on $Ru@SiO_2$, potassium acetate and acetic acid measured in ATR mode.

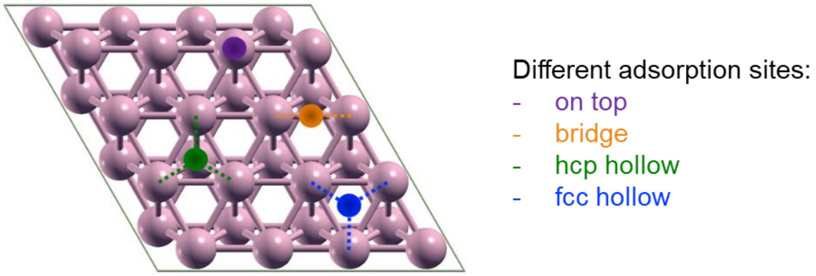

Figure S4. Different adsorption sites available on Ru(0001).

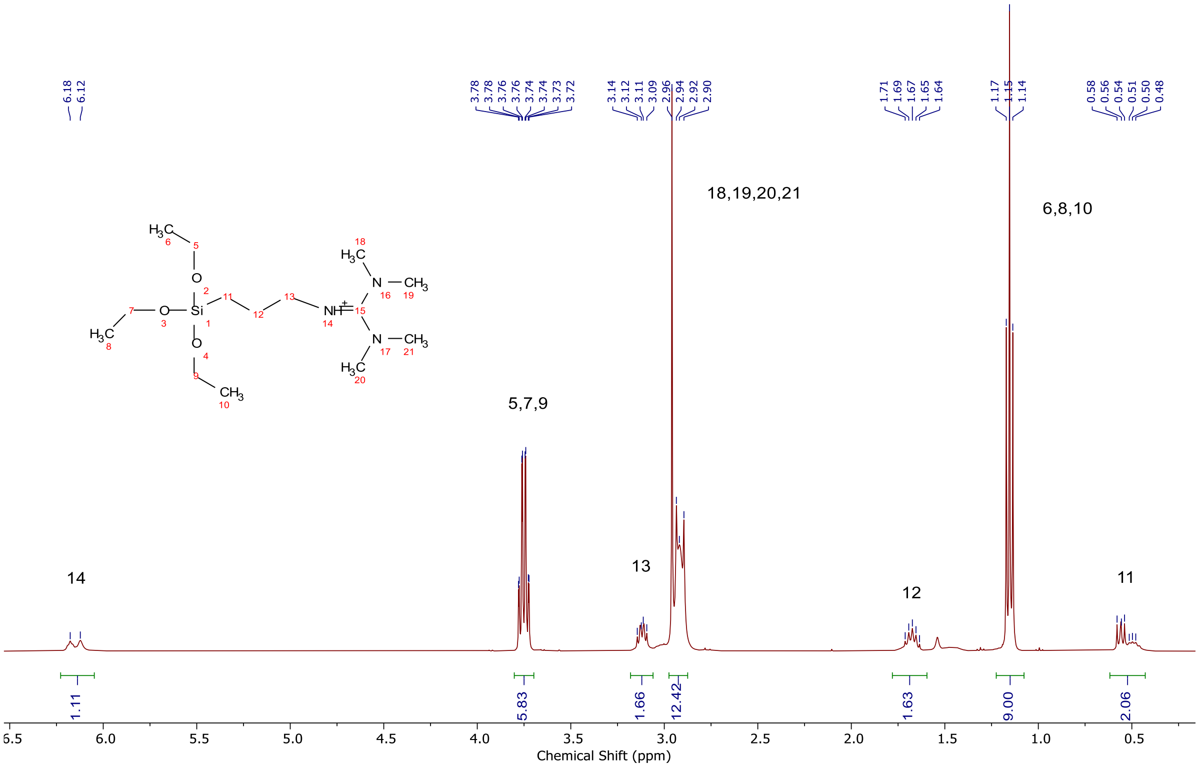

Figure S5. ^{1}H NMR spectrum of 1,1,3,3-Tetramethyl-2-[3-(trimethoxysilyl)propyl] guanidium bis(trifluoromethylsulfonyl)imide in $CDCl_3$.

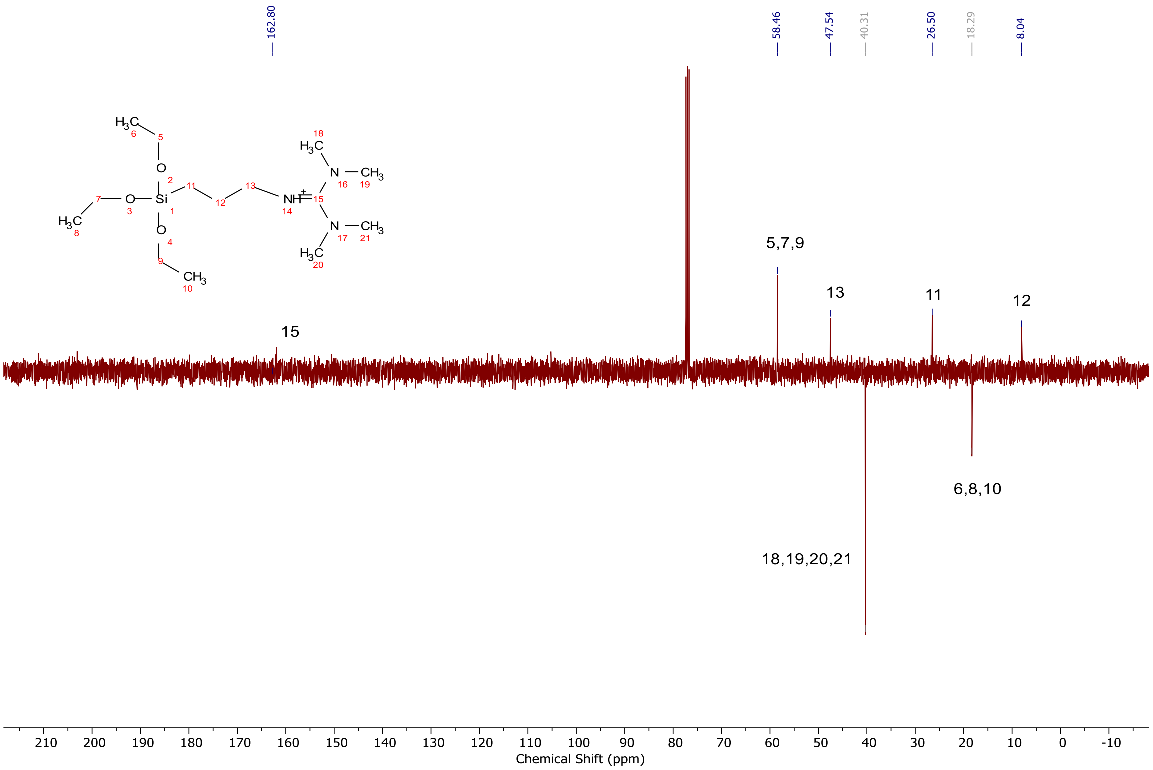

Figure S6. ^{13}C NMR spectrum of 1,1,3,3-Tetramethyl-2-[3-(trimethoxysilyl)propyl] guanidium bis(trifluoromethylsulfonyl)imide in $CDCl_3$.

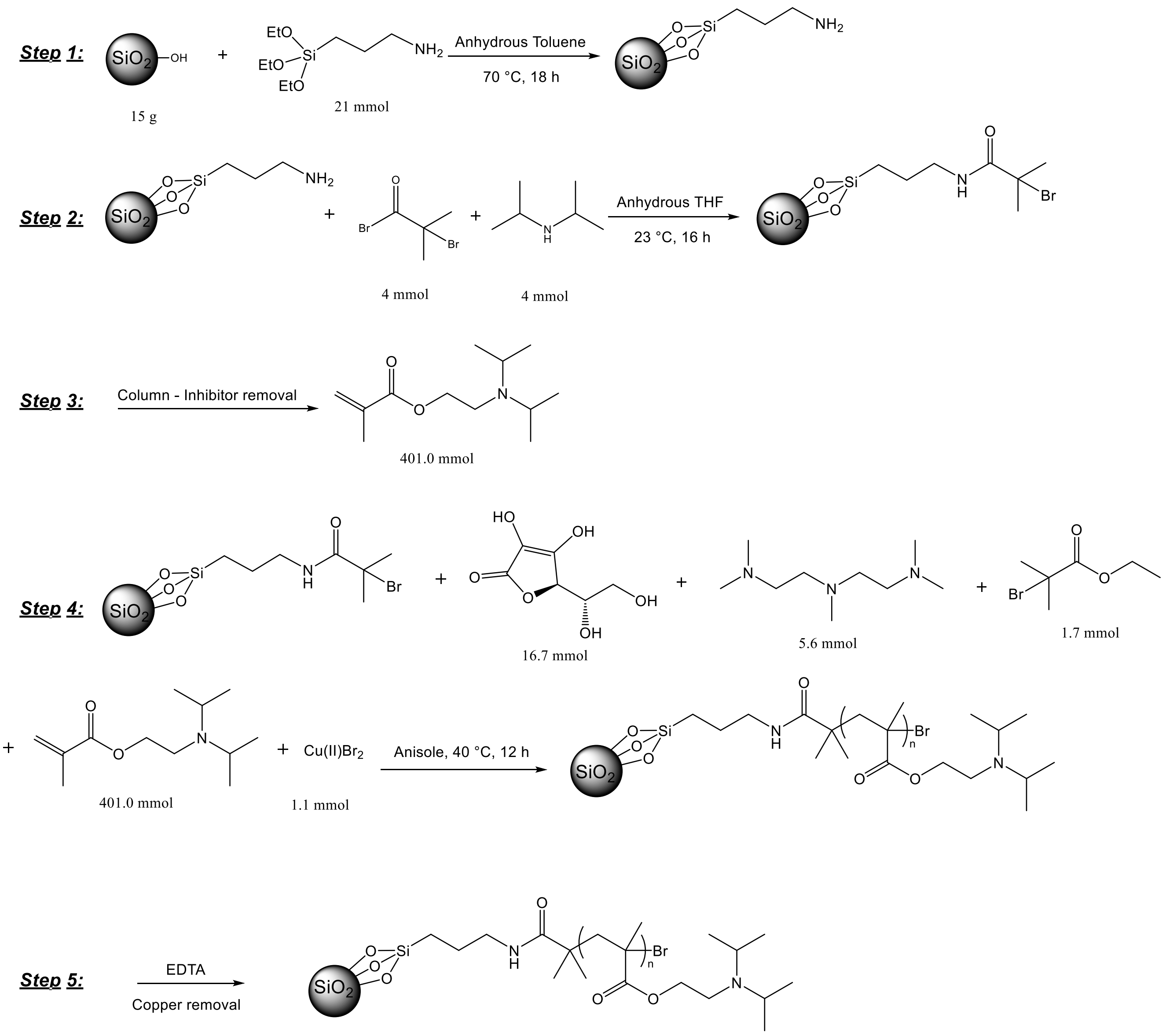

Figure S7. Synthesis of polymer-grafted silica (PGS) used in ref. 30.

$$AE = \frac{\text{Mw (desired product)} * \text{n (desired product)}}{\sum(\text{Mw (reactants)} * \text{n(reactants)})} \times 100 \qquad \text{(eq.1)}$$

Mw = Molecular weight, n = number of mole.

Mn (Number average molar mass of the polymeric molecular modifier) = 21382 g/mol [1]

Number of repeating unit in each polymer chain ~ (21382 g/mol) / (213.2 g/mol) ~ 100

Accessible amines = 1.14 mmol/g[1]

Loading of polymeric molecular modifiers = 1.14 / 100 = 0.0114 mmol/g

Thus, starting from 1 g of SiO_2:

$$AE = \frac{21382 * 0.0114}{221.1 * 1.43 + 227.9 * 0.27 + 213.2 * 26.7 + 194.1 * 0.12} \times 100 = 4\ \%$$

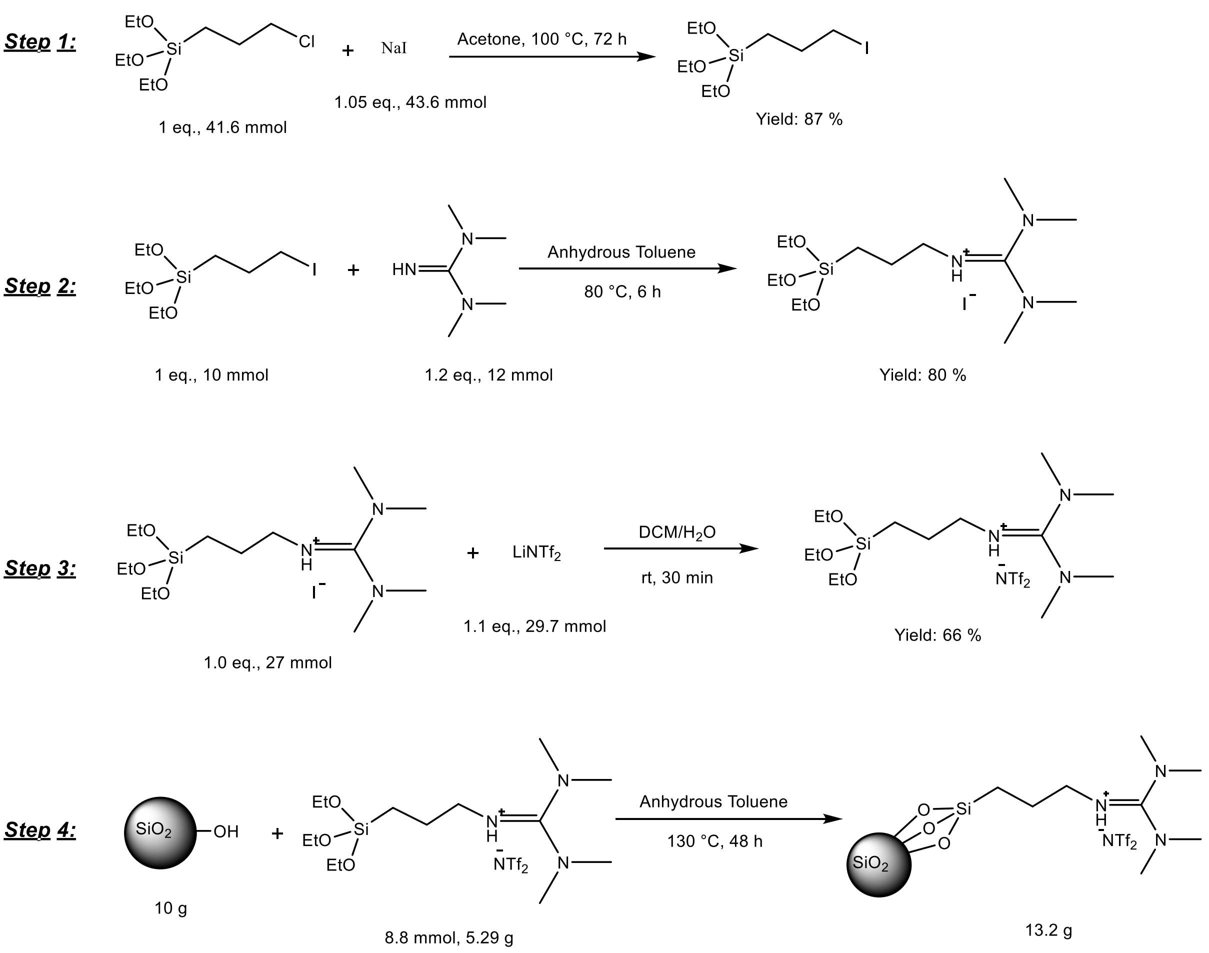

Figure S8. Synthesis of SILPGB.

$$AE = \frac{600.2 * 0.88 * 0.66}{(240.1 * 1.93) + (149.9 * 2.03) + (115.1 * 2) + (286.9 * 1.47)} \times 100 = 24\ \%$$

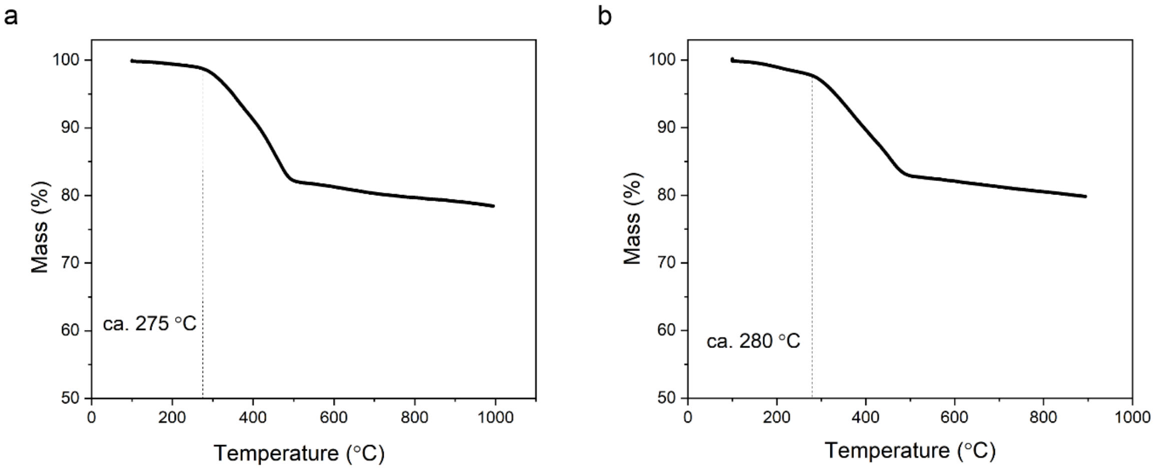

Figure S9. Thermal stability of (a) SILPGB and (b) Ru@SILPGB investigated by thermogravimetric analysis under Ar. Conditions: 5 °C/min under an argon flow of 100 mL/min.

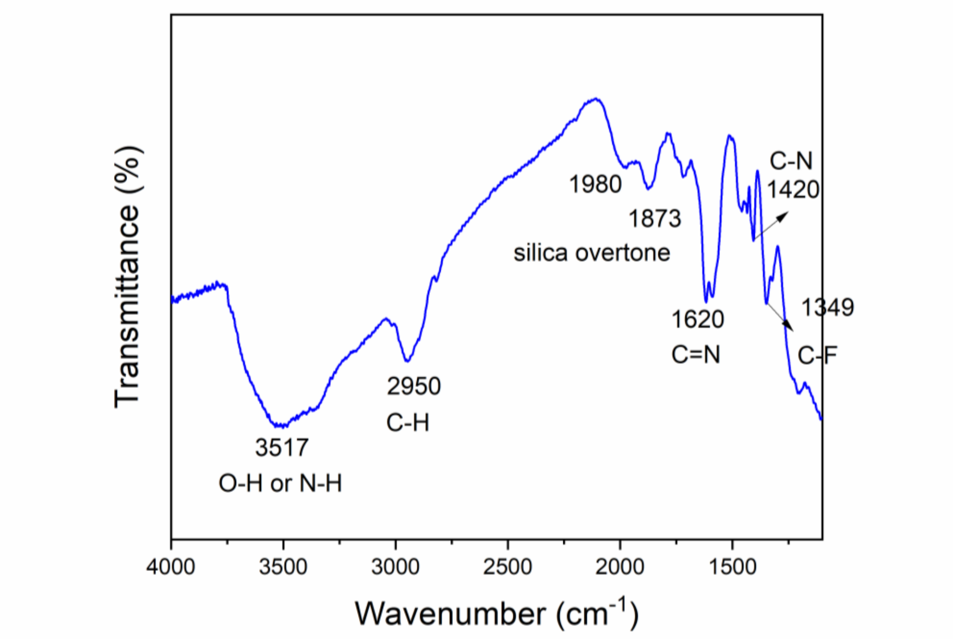

Figure S10. Transmission IR spectrum of Ru@SILP$_{GB}$.

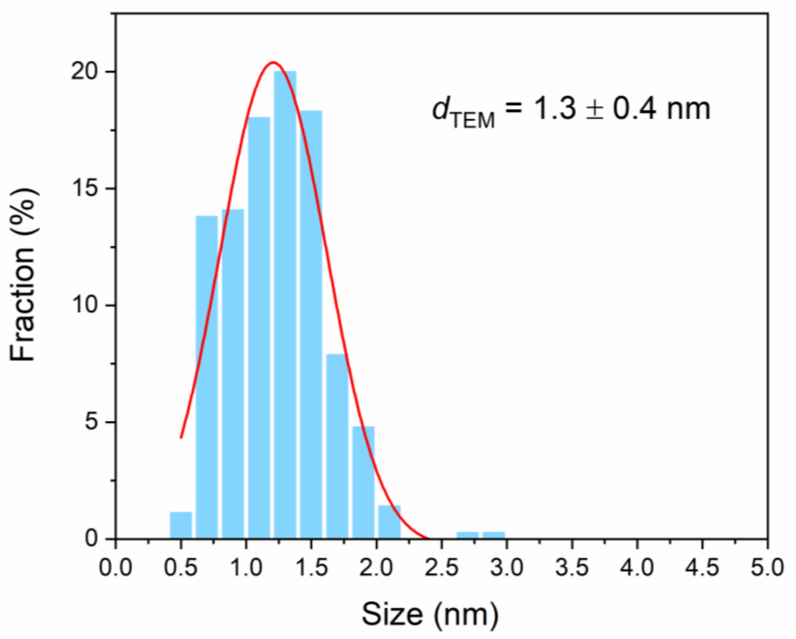

Figure S11. Ru particle size distribution of Ru@SILP$_{GB}$, determined by measuring 355 Ru NPs in TEM images.

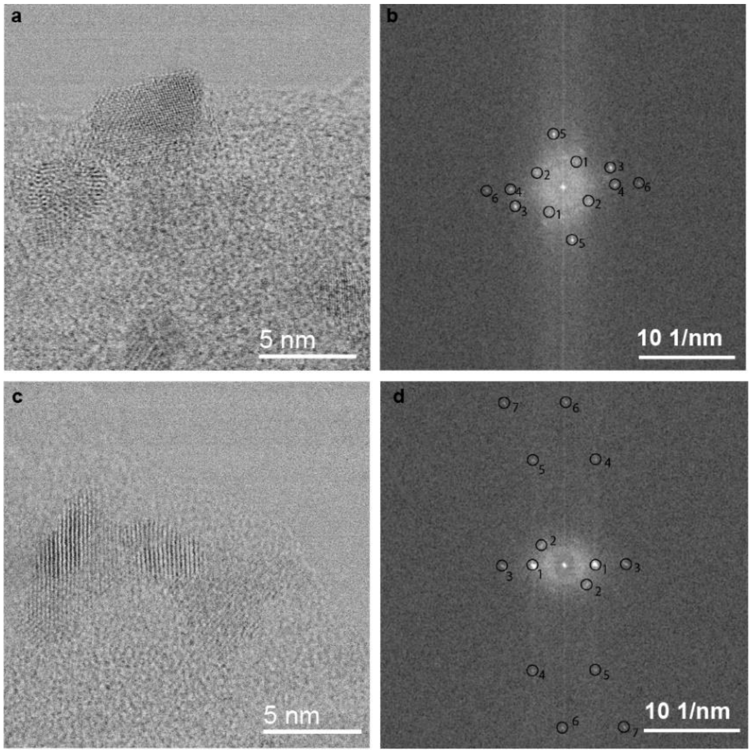

Figure S12. Bright field STEM image (BF-STEM) of Ru@SILP$_{GB}$ (a,c) and the corresponding Fast Fourier Transformation (b,d).

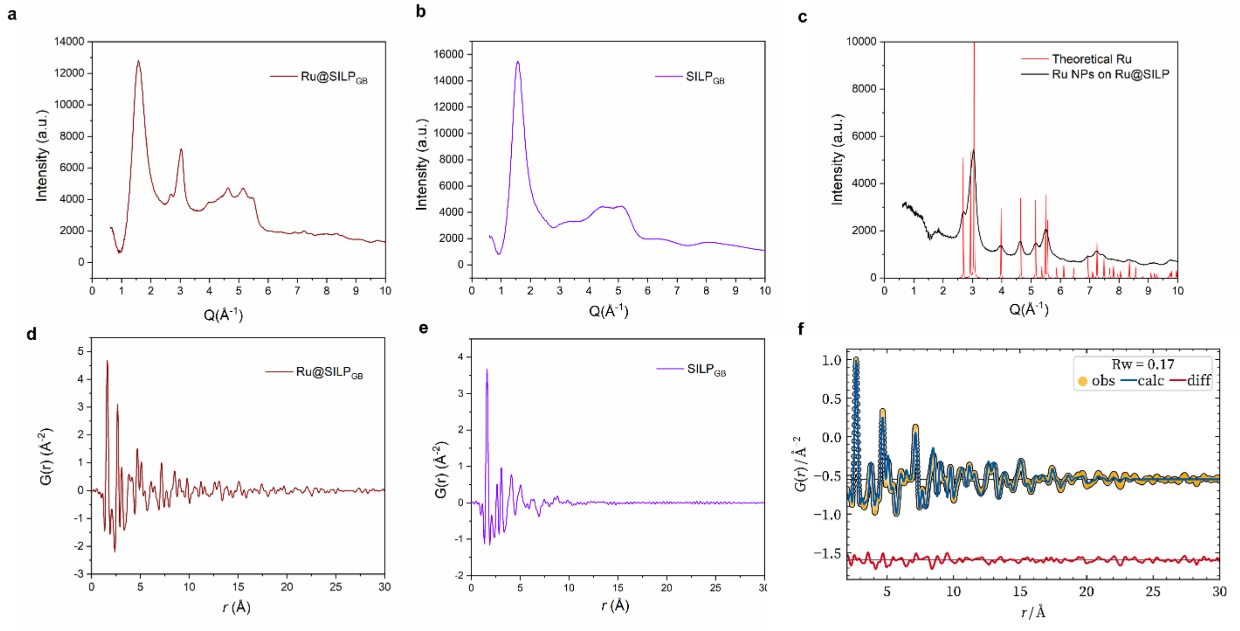

Figure S13. High energy XRD patterns of (a) Ru@SILP$_{GB}$, (b) SILP$_{GB}$, (c) Ru NPs obtained by subtracting the diffraction pattern of SILP$_{GB}$ from the one of Ru@SILP$_{GB}$, complemented by the theoretical XRD pattern of bulk phase Ru *hcp* (#9008513); Pair distribution function (PDF) G(r) obtained by Fourier Transformation of XRD data of (d) Ru@SILP$_{GB}$, (e) SILP$_{GB}$, and (f) Ru NPs. The experimental PDF of Ru NPs (yellow dots) was fitted (blue line) with Ru *hcp*, yielding close to no structural residuals in the difference curve (red).

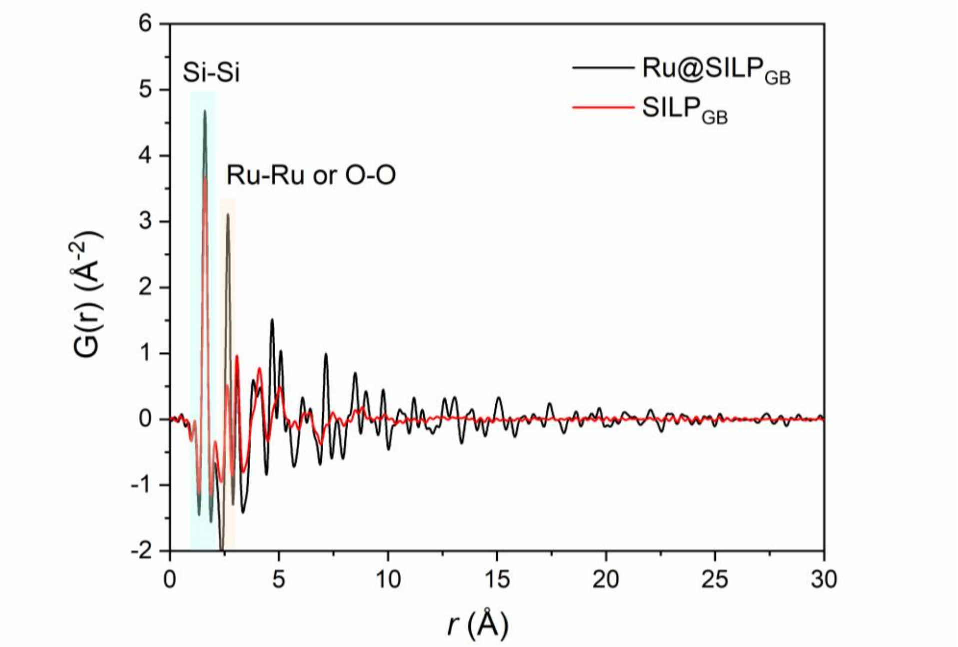

Figure S14. Experimental PDFs G® of Ru@SILP$_{GB}$ (black) and SILP$_{GB}$ (red), highlighting interatomic distances in the sample.

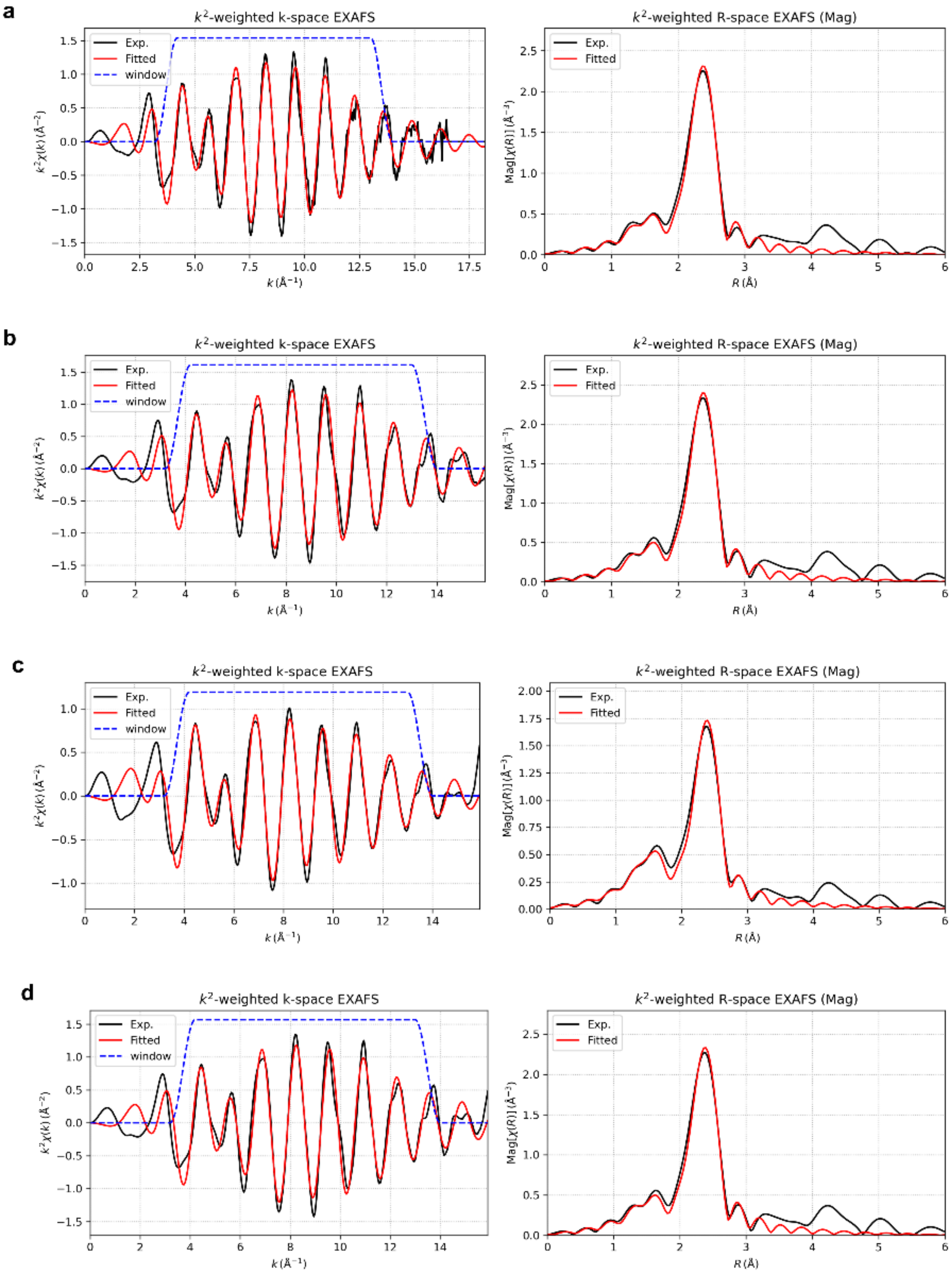

Figure S15. Fitting results of k^2-weighted k-space and R-space FT-EXAFS spectra of (a) Ru@SILP$_{GB}$ catalyst, (b) Ru@SILP$_{IM}$, (c) Ru@Si-Dec and (d) Ru@SiO_2. The experimental EXAFS spectra and fitted spectra are plotted in black and red curves, respectively. The *R*-space spectra (both magnitude and real part) are plotted without phase correction.

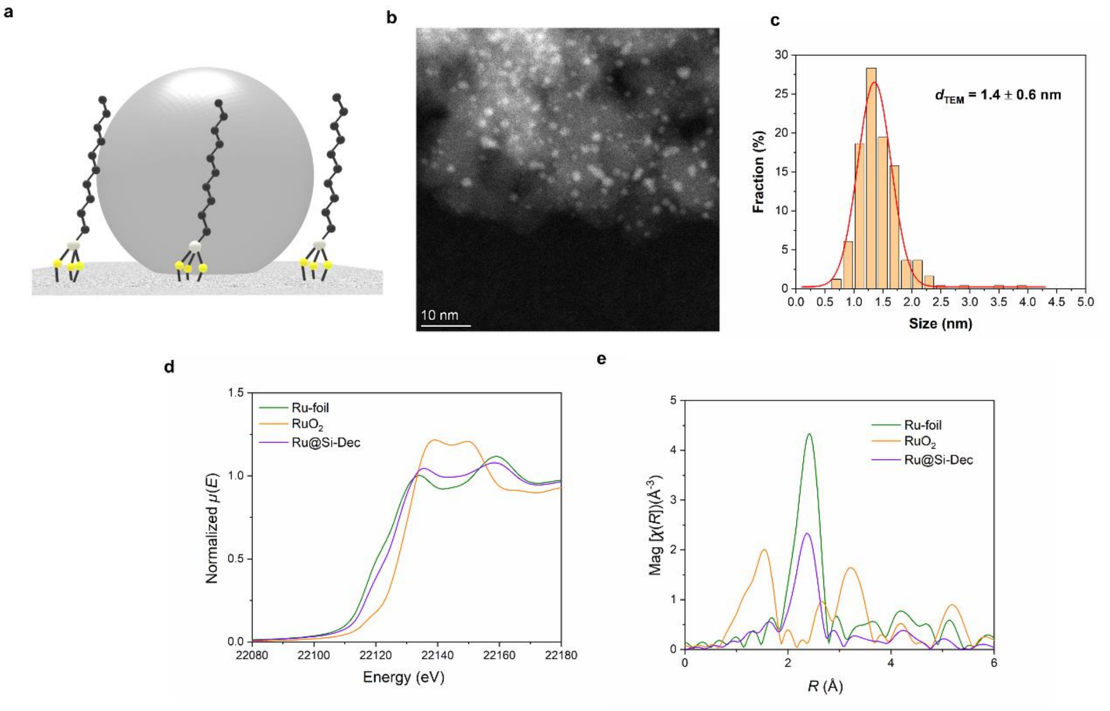

Figure S16. Structure of the Ru@Si-Dec (a) composed of Ru NPs immobilized on silica modified with decyl chain, (b) HAADF-STEM image and (c) particle size distribution by 247 Ru NPs, (d) k^2-weighted R-space FT-EXAFS spectra (plot in Magnitude without phase correction) and K-edge (e) XANES spectra (normalized) for Ru-foil, Ru@Si-Dec and RuO_2 (green, purple and orange curves, respectively).

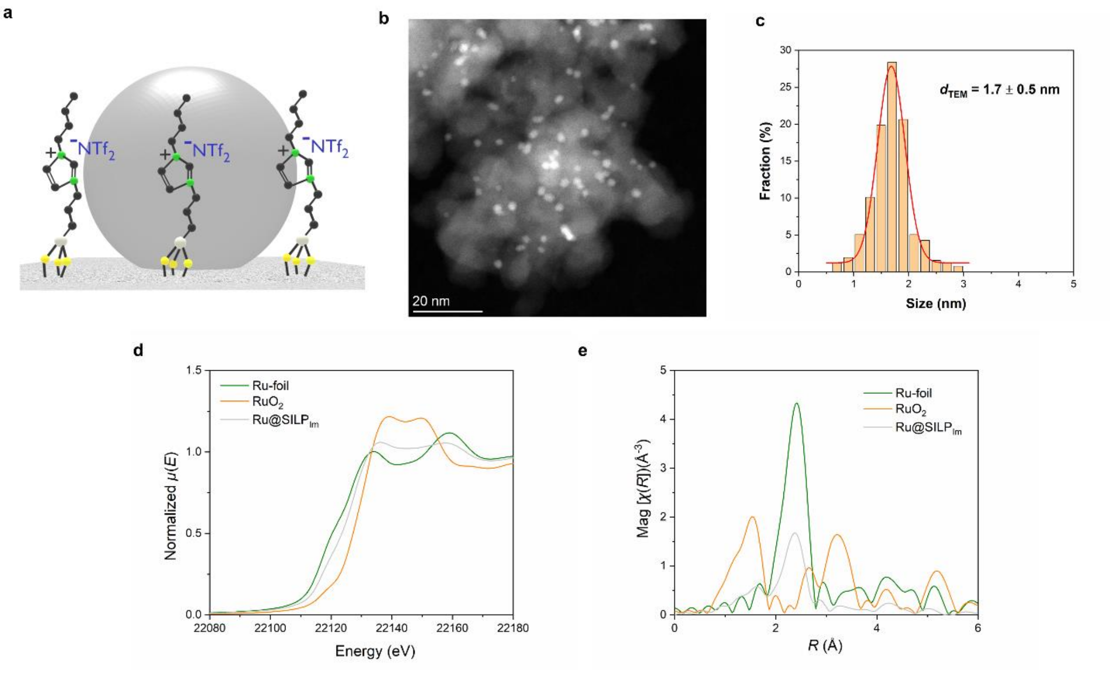

Figure S17. Structure of the Ru@SILP$_{IM}$ (a) composed of Ru NPs immobilized on silica modified with imidazolium-based ionic liquid, (b) HAADF-STEM image, (c) the particle size distribution by 257 Ru NPs, (d) k^2-weighted R-space FT-EXAFS spectra (plot in Magnitude without phase correction) and K-edge (e) XANES spectra (normalized) for Ru-foil, Ru@SILP$_{IM}$ and RuO_2 (green, grey and orange curves, respectively).

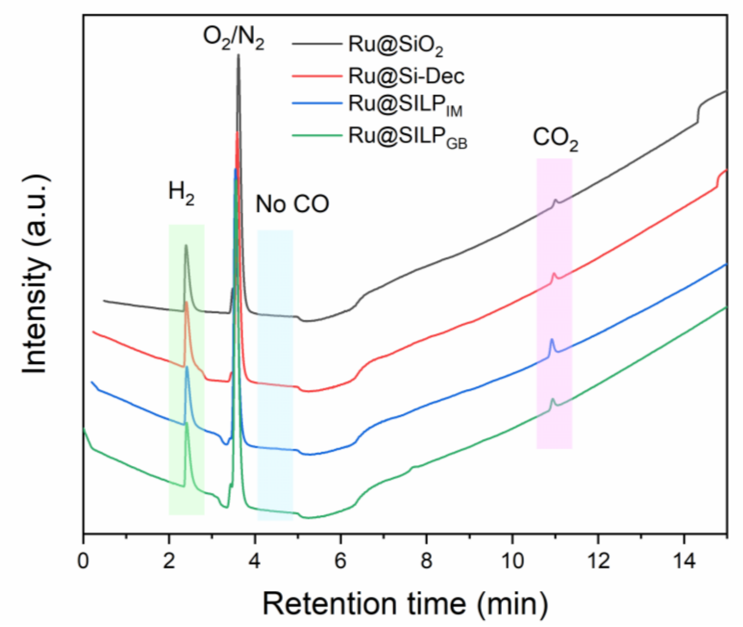

Figure S18. Headspace GC-TCD chromatogram after decomposition of HCOOH in the presence of different catalysts. Reaction conditions: Ru@SiO$_2$, Ru@Si-Dec, Ru@SILP$_{IM}$ or Ru@SILP$_{GB}$ (0.007 mmol Ru), 1,4-dioxane (1 mL), and formic acid (0.22 mmol, 31 eq.), 80 °C, 16 h, H_2 (15 bar).

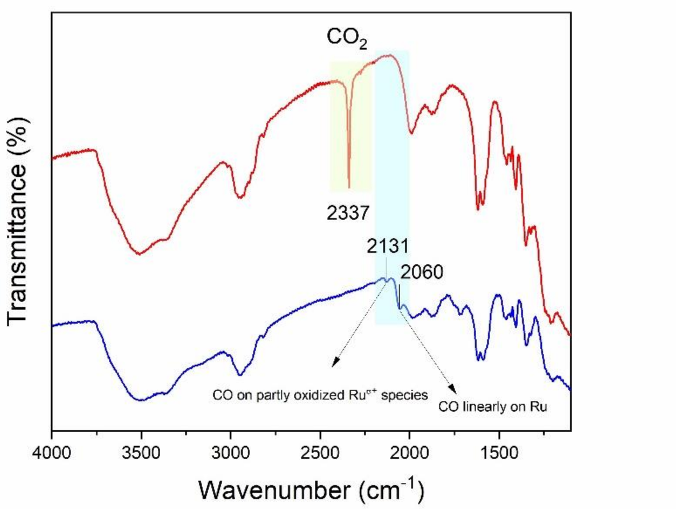

Figure S19. Transmission IR spectra of spent Ru@SILP$_{GB}$ catalyst after the hydrogenation of furfuralacetone under H_2/CO_2 as feed gas (red curve), and the adsorption/desorption of CO on fresh Ru@SILP$_{GB}$ catalyst (blue curve).

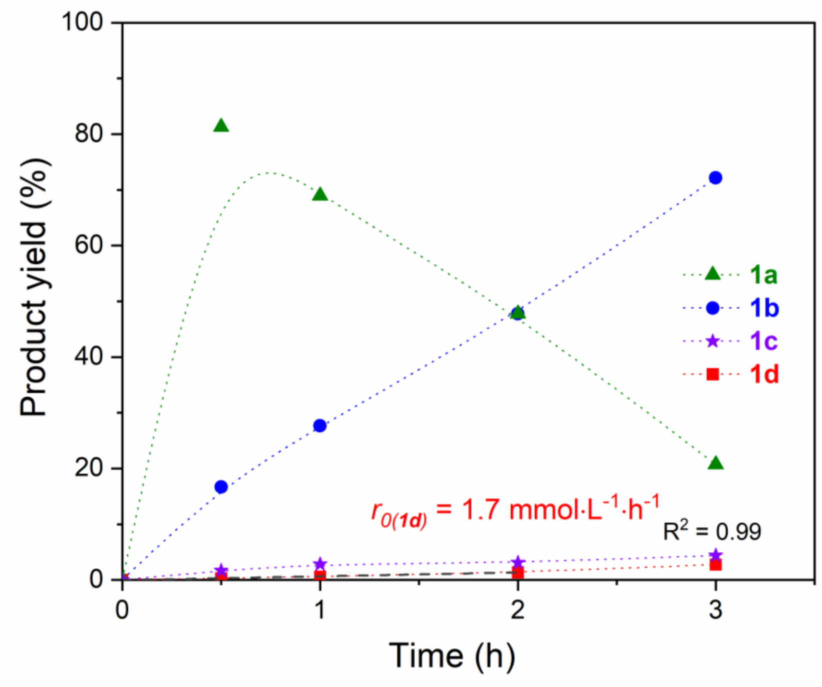

Figure S20. Time profile of the hydrogenation of **1** under H_2/CO_2 as feed gas with added formic acid. Reaction conditions: Ru@SILP$_{GB}$ (0.007 mmol Ru), furfuralacetone (**1**, 0.25 mmol, 35 eq.), 1,4-dioxane (2 mL), 80 °C, 500 rpm, H_2/CO_2 (45 bar, 1:2), formic acid concentration 3.5 mmol L^{-1}.

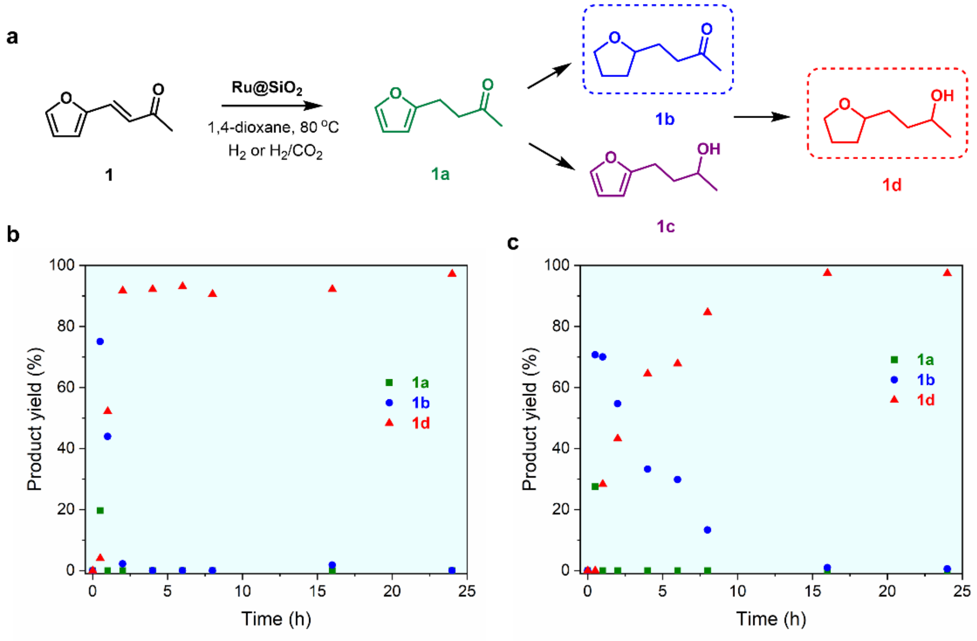

Figure S21. Time profiles of the hydrogenation of **1** (scheme in a) using Ru@SiO$_2$ under (b) H_2 and (c) H_2/CO_2. Reaction conditions: Ru@SiO$_2$ (20 mg, 0.007 mmol Ru), **1** (0.25 mmol, 35 eq.), 1,4-dioxane (2 mL), 80 °C, 500 rpm, (b) H_2 (15 bar) or (c) H_2/CO_2 (45 bar, 1:2). Product yield determined by GC-FID using tetradecane as the internal standard. The by-product is 2,2'-(oxybis(butane-3,1-diyl))bis(tetrahydrofuran). Green squares, blue disks and red triangles represent yields of products **1a**, **1b** and **1d**, respectively.

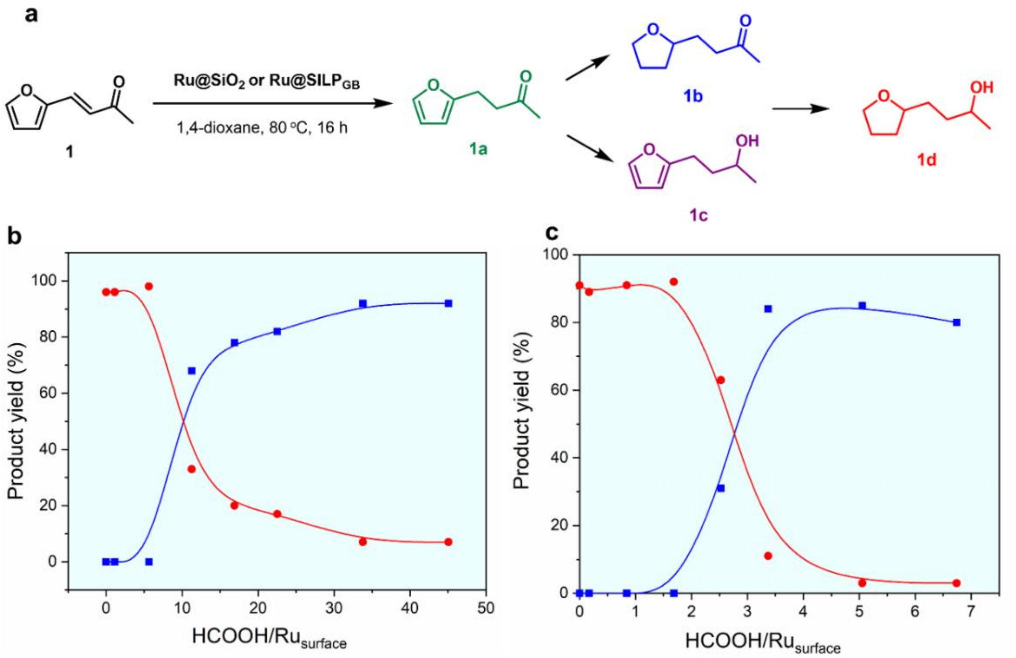

Figure S22. Hydrogenation of furfuralacetone under H_2 with various amounts of formic acid as additive. a) Reaction scheme; b) Ru@SiO_2 as catalyst, c) Ru@$SILP_{GB}$ as catalyst. Reaction conditions: Ru@SiO_2 (20 mg, 0.007 mmol Ru), furfuralacetone (1, 0.25 mmol, 35 eq.), 1,4-dioxane (1 mL), 80 °C, 16 h, 500 rpm, H_2 (15 bar), the red curve and blue curve are for products 1d and product 1b, respectively. HCOOH/$Ru_{surface}$ = molar ratio between HCOOH and Ru centers exposed at the surface of Ru NPs, see SI for details.

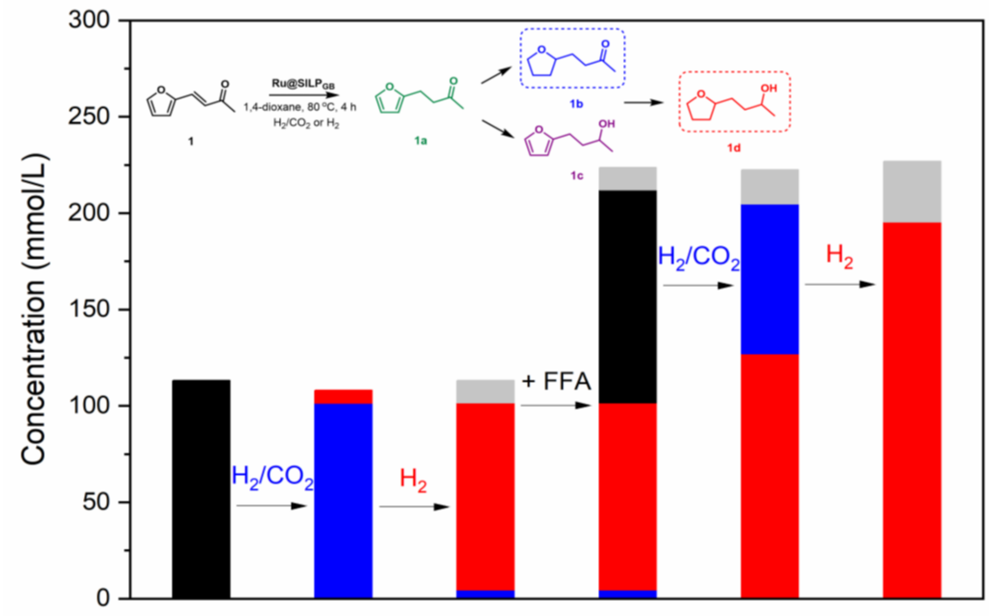

Figure S23. Hydrogenation of **1** with Ru@$SILP_{GB}$ while alternating the feed gas between H_2/CO_2 and H_2 without any washing steps between cycles. Reaction conditions: Ru@$SILP_{GB}$ (20 mg, 0.007 mmol Ru), furfuralacetone (**1**, 0.25 mmol, 35 eq.), 1,4-dioxane (2 mL), H_2/CO_2 (45 bar, 1:2) or H_2 (15 bar), 80 °C, 4 h per cycle. Grey represents the by-product: 2,2'-(oxybis(butane-3,1-diyl))bis(tetrahydrofuran).

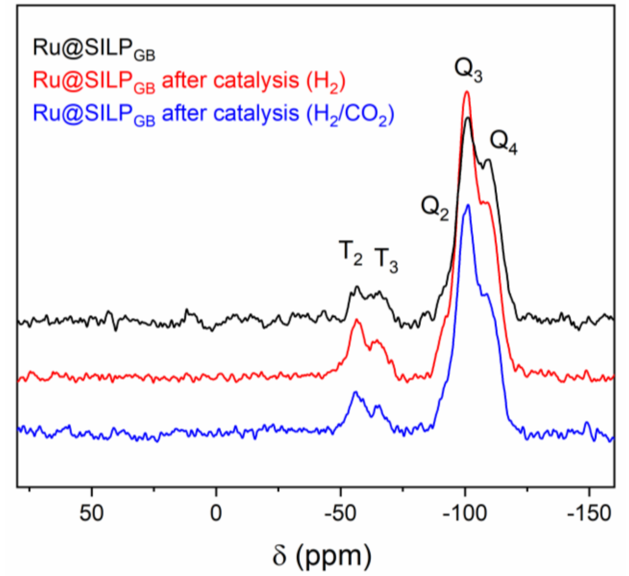

Figure 24. Solid state [29]Si cross polarization-magic angle spinning (CP-MAS) of fresh and spent Ru@SILP$_{GB}$ catalysts after hydrogenation of furfuralacetone (**1**) under H_2 (red) and H_2/CO_2 (blue) as feed gas.

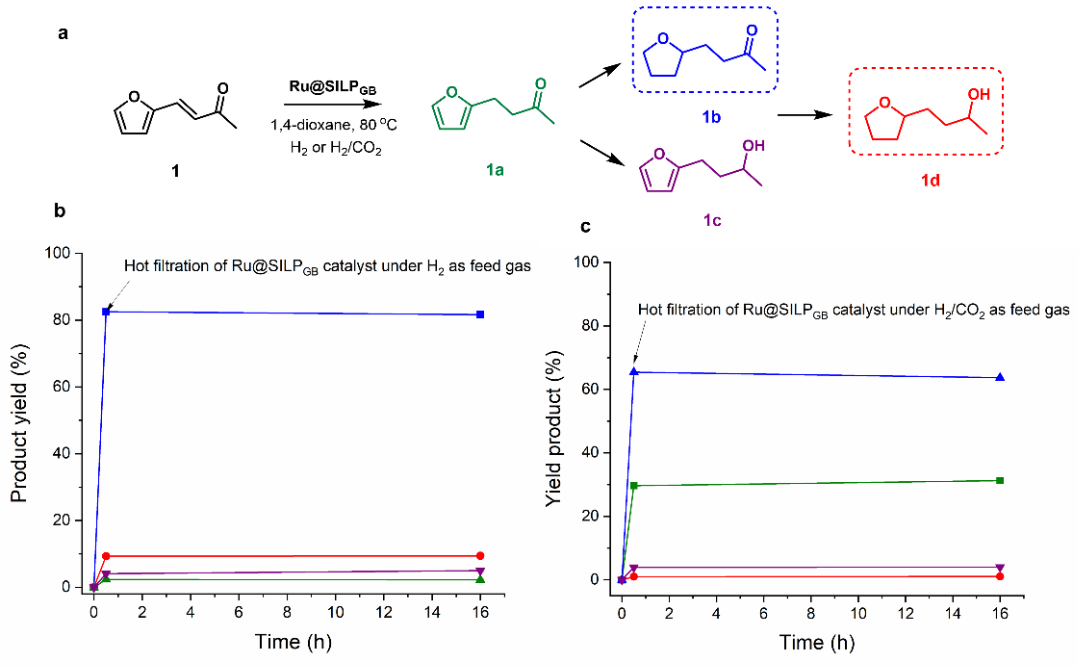

Figure S25. Hot filtration test for the hydrogenation of **1** under (b) H_2 and (c) H_2/CO_2 using Ru@SILP$_{GB}$ as catalyst. After 30 min of reaction, the catalyst powder was removed from the reaction solution by filtration, and the reaction was continued under standard conditions. The results show that the product distribution does not change after removal of the catalyst powder, indicating that the catalytically active sites are on the solid catalyst, and not in solution. Reaction conditions: Ru@SILP$_{GB}$ (20 mg, 0.007 mmol Ru), **1** (0.25 mmol, 35 eq.), 1,4-dioxane (1 mL), 80 °C, 500 rpm, (b) H_2 (15 bar) or (c) H_2/CO_2 (45 bar, 1:2). Product yield determined by GC-FID using tetradecane as the internal standard.

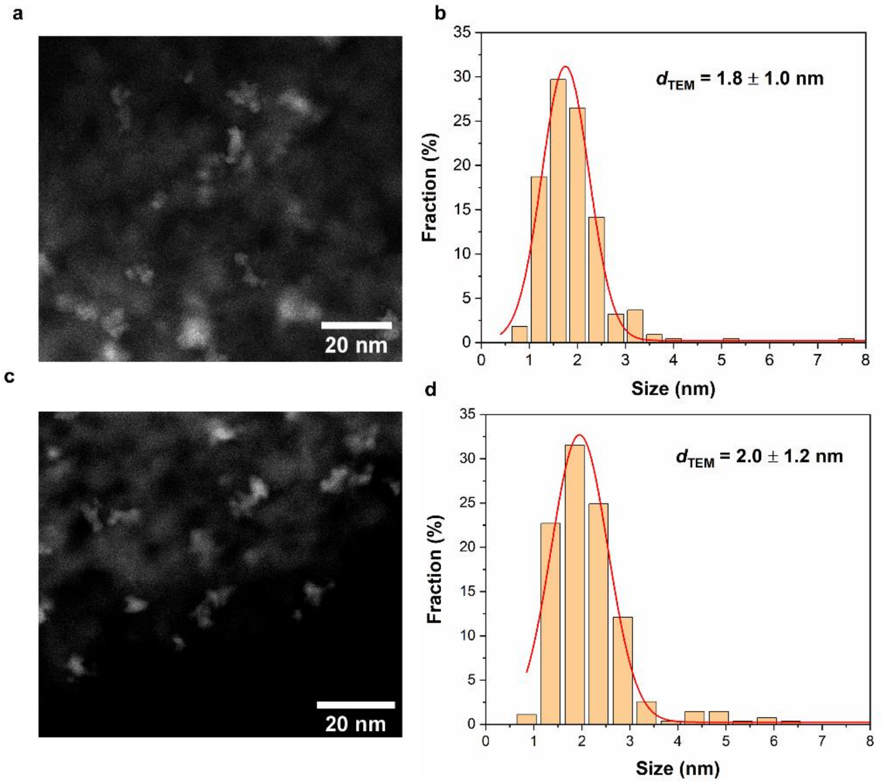

Figure S26. HAADF-STEM images and corresponding particle size distribution of spent Ru@SILP$_{GB}$ catalysts after hydrogenation of furfuralacetone (**1)** under (a) H_2 and (b) H_2/CO_2 as feed gas.

a

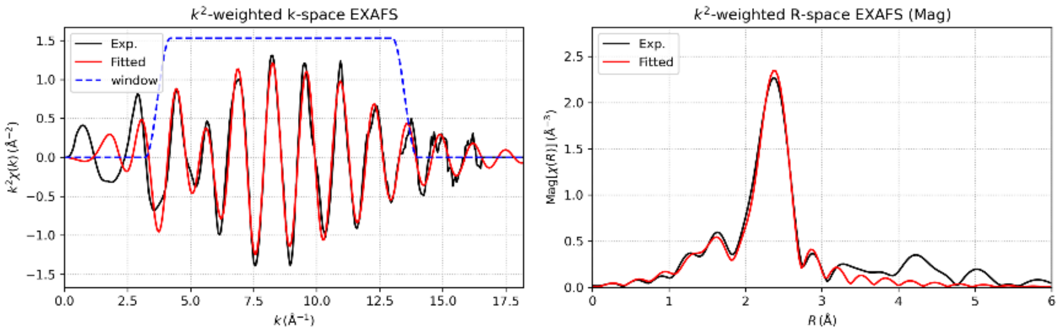

b

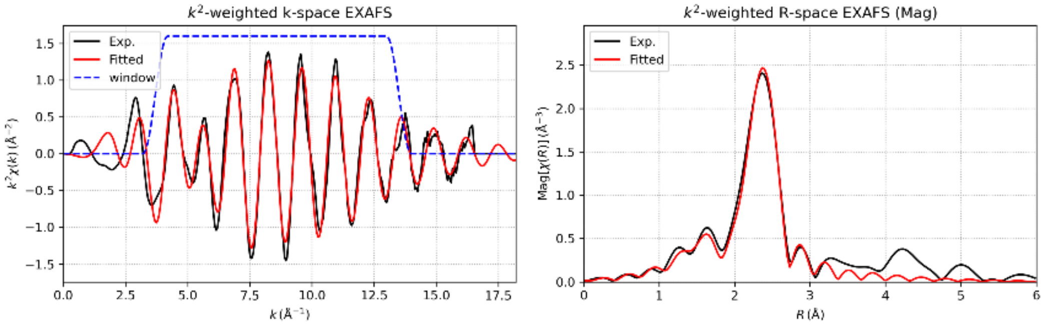

Figure S27. Fitting results of k^2-weighted k-space and R-space FT-EXAFS spectra of spent Ru@SILP$_{GB}$ after reaction under (a) H_2 and (b) H_2/CO_2. The experimental EXAFS spectra and fitted spectra are plotted in black and red curves, respectively. The *R*-space spectra (both magnitude and real part) are plotted without phase correction.

10 Supplementary Tables

Table S1. NPs size, Ru content and N_2 physisorption of SiO_2, $SILP_{GB}$, Ru@$SILP_{GB}$ and reference Ru@$SILP_{IM}$, Ru@Si-Dec and Ru@SiO_2.

Material	NPs size (TEM)	Surface Ru (%)	Ru loading (wt%) (ICP-OES)	BET Surface Area ($m^2 \cdot g^{-1}$)
SiO_2	-	-	-	453 ± 6
$SILP_{GB}$	-	-	-	303 ± 5
Ru@$SILP_{GB}$	1.3 ± 0.4 nm	48.8 ± 9	3.5 ± 0.04	306 ± 4
Ru@$SILP_{IM}$	1.7 ± 0.5 nm	39.2 ± 8	3.8 ± 0.02	232 ± 7
Ru@Si-Dec	1.4 ± 0.6 nm	46.0 ± 12	3.8 ± 0.02	309 ± 12
Ru@SiO_2	2.3 ± 1.0 nm	30.2 ± 8	3.7 ± 0.06	362 ± 20

Table S2. Chemicals entering in the preparation of PGS, including prices and quantities.

Chemicals	M_W (g mol^{-1})	Price (€/g, Sigma-Aldrich)	Mass for 1 g SiO_2 (g)	Price for 1 g SiO_2 (€)	GHS Hazard
3-(Triethoxysilyl)propylamine	221.37	0.51	0.32	0.17	Harmful + Flammable + corrosive
Diisopropylamine	101.19	0.34	0.027	0.01	Harmful + Flammable + Toxic
2-bromo-2-methylpropionyl bromide	229.9	0.52	0.062	0.031	Flammable + corrosive
2-(diisopropylamino)ethyl methacrylate	213.32	3.23	5.7	18.4	Flammable
L-ascorbic acid	176.12	0.63	0.2	0.13	Flammable
Cu(II)Br2	223.35	0.57	0.016	0.01	Flammable + corrosive
N,N,N′,N″,N″-pentamethyldiethylenetriamine	129.24	1.79	0.065	0.12	Toxic + Flammable
Ethyl 2-bromo-2-methylpropionate	167.00	1.81	0.023	0.04	Harmful + corrosive
Cost of converting 1 g of SiO_2 into PGS				18.9	

Table S3. Chemicals entering in the preparation of $SILP_{GB}$, including prices and quantities.

Chemicals	M_W (g mol^{-1})	Price (€/g, Sigma-Aldrich)	Mass for 1 g SiO_2 (g)	Price for 1 g SiO_2 (€)	GHS Hazard
(3-Chloropropyl)triethoxysilane	240.80	0.52	0.46	0.24	Flammable
Sodium iodide	149.89	0.95	0.304	0.29	Toxic + Flammable
1,1,3,3-Tetramethylguanidine	115.18	0.84	0.23	0.2	Flammable
Lithium bis(trifluoromethanesulfonyl)imide	287.09	4.06	0.42	1.71	Toxic + Flammable
Cost of converting 1 g of SiO_2 into $SILP_{GB}$				2.5	

Table S4. The lattice spacing of corresponding spot in Figure S10b and Figure S10d.

Image	Spot	Lattice-spacing (Å)	*hkl*
Figure S10b	1	2.34	1 -1 0
	2	2.28	1 -1 0
	3	1.30	2 -1 0
	4	1.28	2 -1 0
	5	1.24	1 -1 3
	6	0.87	3 -2 1
Figure S10d	1	2.12	0 0 2
	2	2.23	1 -1 0
	3	1.08	2 -2 1

Table S5. Structural parameters obtained from EXAFS fitting of all Ru-based catalysts.

Sample	Scattering path	S_0^2 (amp)	C.N.	R (Å)	R_{ref} (Å)	σ^2 (Å^2)	ΔE_0 (eV)
Ru-foil	Ru-Ru	0.70 ± 0.06	12 (fixed)	2.68 ± 0.01	2.65	0.0033 ± 0.0001	4.5 ± 0.8
$Ru@SILP_{GB}$	Ru-O	0.70 (fixed)	1.3 ± 0.5	1.97 ± 0.03		0.0044 ± 0.0005	-3.7 ± 0.9
	Ru-Ru		9.1 ± 0.9	2.68 ± 0.01		0.0054 ± 0.0006	
$Ru@SiO_2$	Ru-O	0.70 (fixed)	1.4 ± 0.5	1.97 ± 0.03		0.0046 ± 0.0006	-3.5 ± 0.9
	Ru-Ru		9.1 ± 1.0	2.68 ± 0.01		0.0054 ± 0.0006	
$Ru@SILP_{IM}$	Ru-O	0.70 (fixed)	2.1 ± 0.5	1.98 ± 0.02		0.0055 ± 0.0007	-3.5 ± 0.9
	Ru-Ru		7.5 ± 0.9	2.68 ± 0.01		0.0061 ± 0.0007	
Ru@Si-Dec	Ru-O	0.70 (fixed)	1.2 ± 0.5	1.97 ± 0.03		0.0041 ± 0.0006	-3.6 ± 0.9
	Ru-Ru		9.3 ± 0.9	2.68 ± 0.01		0.0053 ± 0.0006	
$Ru@SILP_{GB}$ after catalysis under H_2	Ru-O	0.70 (fixed)	1.5 ± 0.5	1.98 ± 0.02		0.0046 ± 0.0005	-3.5 ± 0.9
	Ru-Ru		9.5 ± 0.9	2.68 ± 0.01		0.0056 ± 0.0005	
$Ru@SILP_{GB}$ after catalysis under H_2/CO_2	Ru-O	0.70 (fixed)	1.4 ± 0.5	1.98 ± 0.03		0.0042 ± 0.0005	-3.5 ± 0.9
	Ru-Ru		9.2 ± 0.9	2.68 ± 0.01		0.0052 ± 0.0006	
RuO_2 (Reference)	Ru-O		2		1.94		
	Ru-O		4		1.99		
	Ru-Ru		2		3.10		
	Ru-Ru		8		3.54		

S_0^2 = amplitude reduction factor, this value is determined by fitting the EXAFS of Ru foil and used as a fixed parameter for the EXAFS fitting of other Ru samples; C.N. = coordination number; R = interatomic distance; R_{ref} = interatomic distances of reference materials. The interatomic distances in Ru metal and RuO_2 are obtained from the standard crystal structure retrieved from the Crystal Open Database (entry ID of Ru: 9008513; entry ID of RuO_2: 1000058). σ^2 = Debye-Waller factor. ΔE_0 = energy shift refers to the E_0 position in the EXAFS fitting model.

Table S6. Parameter optimization for the hydrogenation of CO_2 to HCOOH using Ru@SILP$_{GB}$ as catalyst.

Entry	Solvent	P_{H_2} (bar)	P_{CO_2} (bar)	[HCOOH] (mmol/L)	HCOOH/Ru molar ratio
1	1-butanol	15	15	0.5	0.07
2	1,4-dioxane	15	5	0.7	0.10
3	1,4-dioxane	15	15	1.3	0.19
4	1,4-dioxane	15	20	2.3	0.33
5	1,4-dioxane	15	30	3.1	0.44
6	THF-d8	15	5	0.1	0.01
7	THF-d8	15	15	0.4	0.06
8	THF-d8	15	20	0.5	0.07
9	THF-d8	15	30	1.5	0.21

Reaction conditions: Ru@SILP$_{GB}$ (20 mg, 0.007 mmol Ru), solvent (1 mL), H_2 (bar), CO_2 (bar), 80 °C, 16 h, concentration of HCOOH determined by ^{1}H NMR spectra using $CHCl_3$ as an internal standard.

Table S7. Parameter optimization for the hydrogenation of furfuralacetone (**1**) using Ru@SILP$_{GB}$ under H_2 or H_2/CO_2 as feed gas.

Entry	T (°C)	Solvent	P_{H_2} (bar)	P_{CO_2} (bar)	X (%)	Y_{1d} (%)	Y_{1b} (%)
1	100	0.5 mL Butanol	15	-	>99	91	0
2	100	0.5 mL Butanol	15	15	>99	76	15
3	100	0.5 mL DME	15	15	>99	90	1
4	100	0.5 mL heptane	15	15	>99	86	7
5	100	0.5 mL EG	15	15	>99	44	31
6	100	0.5 mL decalin	15	15	>99	94	2
7	100	0.5 mL Dioxane	15	15	>99	40	54
8	80	0.5 mL Dioxane	15	15	>99	46	50
9	80	1 mL Dioxane	15	15	>99	57	39
10	70	1 mL Dioxane	15	15	>99	41	56
11	60	1 mL Dioxane	15	15	>99	44	54
12	90	1 mL Dioxane	15	15	>99	32	64
13	80	2 mL Dioxane	15	15	>99	24	73
14	80	1 mL Dioxane	15	20	>99	32	65
15	80	1 mL Dioxane	15	30	>99	10	90
16	80	1 mL Dioxane	15	-	>99	91	0

Reaction conditions: Ru@SILP$_{GB}$ (20 mg, 0.007 mmol Ru), furfuralacetone (**1**, 0.25 mmol, 35 eq.), 1,4-dioxane (0.65 mL), H_2 (bar), CO_2 (bar), T (°C), 16 h, X = conversion, Y = yield, determined by GC-FID using tetradecane as an internal standard, the by-product is 2,2'-(oxybis(butane-3,1-diyl))bis(tetrahydrofuran).

Table S8. BET surface area and Ru content (ICP-OES) in Ru@SILP$_{GB}$ after hydrogenation of **1** under H_2 or H_2/CO_2 as feed gas.

Material	BET Surface Area ($m^2 \cdot g^{-1}$)	Ru (wt%)
Ru@SILP$_{GB}$ after catalysis under H_2	330	3.7
Ru@SILP$_{GB}$ after catalysis under H_2/CO_2	354	4.0

Table S9. Ru content (ICP-MS) in reaction mixtures after hydrogenation of furfuralacetone using Ru@SILP$_{GB}$ under H_2 or H_2/CO_2 as feed gas.

Material	Ru (ppm)	Lost content (%)
Reaction mixture under H_2 as feed gas	2	0.28
Reaction mixture under H_2/CO_2 as feed gas	1	0.14

Table S10. Optimization steps for the hydrogenation of ketone-containing derivatives.

Substrate	T (°C)	P_{H_2} (bar)	P_{CO_2} (bar)	*X* (%)	$Y_{saturated\ alcohol}$ (%)	$Y_{saturated\ ketone}$ (%)
**6**	80	15	-	>99	94	0
	80	15	30	>99	32	65
	60	15	-	>99	97	0
	60	15	30	>99	31	68
	40	15	30	>99	33	67
	30	15	50	>99	12	88
	30	15	-	>99	89	10
**7**	80	15	-	>99	55	45
	80	15	30	>99	9	91
	100	25	-	>99	80	20
	100	15	-	>99	50	50
	100	15	45	>99	5	95
**9**	80	15	-	>99	93	7
	80	15	30	>99	66	34
	60	15	-	>99	95	5
	60	15	30	>99	47	53

Reaction conditions: $Ru@SILP_{GB}$ (20 mg, 0.007 mmol Ru), substrate (0.25 mmol, 35 eq.), 1,4-dioxane (1 mL), 16 h, H_2, CO_2, 500 rpm. *X* = conversion, *Y* = yield, determined by GC-FID using tetradecane as an internal standard.

Table S11. Hydrogenation of furfuralacetone (**1**) under H_2 with acetic acid as an additive.

Entry	Catalyst	P_{H_2} (bar)	P_{CO_2} (bar)	*X* (%)	Y_{1d} (%)	Y_{1b} (%)	*Acetic acid/Ru_{surface}*
1	$Ru@SILP_{GB}$	15	-	>99	95	0	7
2	$Ru@SILP_{GB}$	15	-	>99	95	0	14
3	$Ru@SILP_{GB}$	15	-	>99	93	0	34
4	$Ru@SILP_{GB}$	15	-	>99	94	0	67
5	$Ru@SiO_2$	15	-	>99	93	0	7
6	$Ru@SiO_2$	15	-	>99	93	0	37

Reaction conditions: $Ru@SILP_{GB}$ or $Ru@SiO_2$ (20 mg, 0.007 mmol Ru), furfuralacetone (**1**, 0.25 mmol, 35 eq.), 1,4-dioxane (1 mL), H_2 (bar), CO_2 (bar), T (80 °C), 16 h, acetic acid, *X* = conversion, *Y* = yield, determined by GC-FID using tetradecane as an internal standard, the by-product is 2,2'-(oxybis(butane-3,1-diyl))bis(tetrahydrofuran).

11 Isolation of products

General procedure: Ru@SILP$_{GB}$ (60 mg) and substrates (0.75 mmol) were dispersed in 1,4-dioxane (2.5 mL) in a glass insert and placed in a high-pressure autoclave, then sealed and pressurized with H_2 or H_2/CO_2. After purging, the reaction mixture was stirred at desired temperature in an aluminum heating block. Once the reaction was finished, the reactor was cooled in an ice bath and carefully vented. The catalyst and the solution were separated and collected by centrifugation.

4-(tetrahydrofuran-2-yl)butan-2-ol (1d) Compound **1d** was prepared according to the

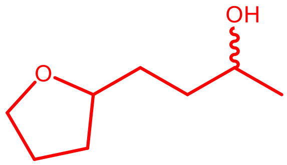

general procedure. The product was isolated from the reaction mixture using flash column chromatography (Pentane: EtOAc = 60:40) in 80% yield. ^{1}H NMR (400 MHz, Chloroform-*d*): δ (ppm) = 3.84 - 3.63 (m, 4H), 1.97 - 1.73 (m, 3H), 1.65 - 1.49 (m, 3H), 1.46 - 1.36 (m, 2H), 1.12 (dd, *J* = 6.2, 2.2 Hz, 3H). ^{13}C NMR (101 MHz, Chloroform-*d*): *δ* (ppm) = 79.7/79.4, 68.1/67.6, 67.8, 36.6/36.0, 32.6/31.6, 31.5/31.4, 25.7/25.6, 23.6/23.3. Mixture of isomers, NMR data are consistent with literature.[26]

4-(tetrahydrofuran-2-yl)butan-2-one (1b) Compound **1b** was prepared according to the

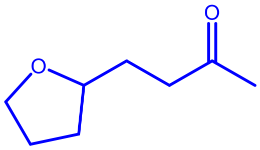

general procedure. The product was isolated from the reaction mixture using flash column chromatography (Pentane: EtOAc = 90:10) in 81% yield. ^{1}H NMR (400 MHz, Chloroform-*d*): δ = 3.79-3.70 (m, 2H), 3.62 (q, *J* = 8 Hz, 1H), 2.63 - 2.35 (m, 2H), 2.08 (s, 3H), 1.95 - 1.87 (m, 1H), 1.85 - 1.72 (m, 3H), 1.69 - 1.60 (m, 1H), 1.44 - 1.35 (m, 1H). ^{13}C NMR (101 MHz, Chloroform-*d*): δ (ppm) = 208.72, 78.29, 67.65, 40.43, 31.31, 29.95, 29.48, 25.70.[27]

1-(tetrahydrofuran-2-yl)propan-2-ol (2b) Compound **2b** was prepared according to the

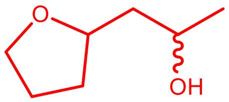

general procedure. The product was isolated from the reaction mixture using flash column chromatography (DCM: Methanol = 100:1.5) in 84% yield. ^{1}H NMR (400 MHz, Chloroform-*d*): δ (ppm) = 4.08 - 3.89 (m, 2H), 3.85 - 3.78 (m, 1H), 3.74 - 3.55 (m, 1H), 1.96 - 1.77 (m, 3H), 1.68 - 1.39 (m, 3H), 1.13 (dd, *J* = 16.6, 6.3 Hz, 3H). ^{13}C NMR (101 MHz, Chloroform-*d*): δ (ppm) = 79.8/76.8, 68.1/65.4, 68.0/67.9, 43.97/42.62, 32.2/31.2, 25.64/25.19, 23.37/23.36. Mixture of isomers, NMR data are consistent with literature.[26]

1-(tetrahydrofuran-2-yl)propan-2-one (2a) Compound **2a** was prepared according to the general procedure. The product was isolated from the reaction mixture using flash column chromatography (DCM: Methanol = 100:0.5) in 79% yield. ^{1}H NMR (400 MHz, Chloroform-*d*): δ (ppm) = 4.15 (p, *J* = 6.7, 1H), 3.82 - 3.77 (m, 1H), 3.69 - 3.64 (m, 1H), 2.71 - 2.65 (m, 1H), 2.52 - 2.47 (m, 1H), 2.12 (s, 3H), 2.07 -1.99 (m, 1H), 1.86 - 1.79 (m, 2H), 1.45 - 1.36 (m, 1H). ^{13}C NMR (101 MHz, Chloroform-*d*): δ (ppm) = 207.39, 74.98, 67.87, 49.59, 31.50, 30.68, 25.55.[29]

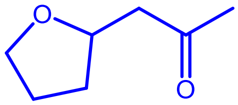

1-(tetrahydrofuran-2-yl)butan-2-ol (3b) Compound **3b** was prepared according to the general procedure. The product was isolated from the reaction mixture using flash column chromatography (DCM: Methanol = 100:1.5) in 76% yield. ^{1}H NMR (400 MHz, Chloroform-*d*): δ (ppm) = 4.06 - 3.94 (m, 1H), 3.86 - 3.80 (m, 1H), 3.73 - 3.60 (m, 2H), 2.02 - 1.74 (m, 3H), 1.70 - 1.57 (m, 1H), 1.55 - 1.33 (m, 4H), 0.90 - 0.85 (m, 3H). ^{13}C NMR (101 MHz, Chloroform-*d*): δ (ppm) = 80.03/76.93, 73.29/70.67, 67.97/67.87, 41.55/40.49, 32.28/31.22, 30.22/30.16, 26.66/25.21, 10.12/9.79. Mixture of isomers, NMR data are consistent with literature.[30]

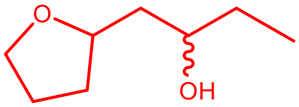

1-(tetrahydrofuran-2-yl)butan-2-one (3a) Compound **3a** was prepared according to the general procedure. The product was isolated from the reaction mixture using flash column chromatography (DCM: Methanol = 100:0.5) in 84% yield. ^{1}H NMR (400 MHz, Chloroform-*d*): δ (ppm) = 4.16 (p, *J* = 6.7, 1H), 3.82 - 3.76 (m, 1H), 3.68 - 3.63 (m, 1H), 2.67 - 2.64 (m, 1H), 2.49 - 2.39 (m, 3H), 2.07 - 1.99 (m, 1H), 1.86 - 1.78 (m, 2H), 1.44 - 1.35 (m, 1H), 0.99 (t, *J* = 7.3 Hz, 3H). ^{13}C NMR (101 MHz, Chloroform-*d*): δ (ppm) = 209.93, 75.15, 67.83, 48.32, 36.73, 31.53, 25.56, 7.58.[29]

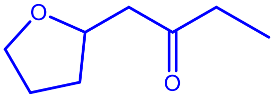

1-(tetrahydrofuran-2-yl)pentan-2-ol (4b) Compound **4b** was prepared according to the general procedure. The product was isolated from the reaction mixture using flash column chromatography (DCM: Methanol = 100:1.5) in 75% yield. ^{1}H NMR (400 MHz, Chloroform-*d*): δ (ppm) = 4.10 - 3.89 (m, 1H), 3.89 - 3.69 (m, 2H), 3.69 -3.51 (m, 1H), 2.04 - 1.71 (m, 3H), 1.69 - 1.51 (m, 2H), 1.50 - 1.24 (m, 5H), 0.88 - 0.84 (m, 3H). ^{13}C NMR

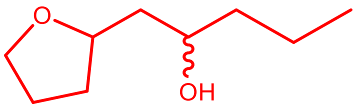

(101 MHz, Chloroform-*d*): δ (ppm) = 80.59/76.69, 71.70/69.04, 67.98/67.89, 42.13/40.94, 39.72/39.57, 32.28/31.25, 25.65/25.21, 19.47/18.66, 14.16/14.13. Mixture of isomers, NMR data are consistent with literature.[30]

1-(tetrahydrofuran-2-yl)pentan-2-one (4a) Compound **4a** was prepared according to

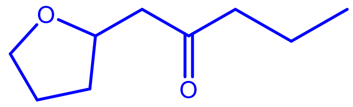

the general procedure. The product was isolated from the reaction mixture using flash column chromatography (DCM: Methanol = 100:0.5) in 81% yield. ^{1}H NMR (400 MHz, Chloroform-*d*): δ (ppm) = 4.16 (p, *J* = 6.7, 1H), 3.81 - 3.76 (m, 1H), 3.68 - 3.63 (m, 1H), 2.67 - 2.63 (m, 1H), 2.45 - 2.42 (m, 1H), 2.38 - 2.32 (m, 2H), 2.07 - 1.97 (m, 1H), 1.86 - 1.78 (m, 2H), 1.54 (h, *J* = 7.4 Hz, 2H), 1.43 - 1.34 (m, 1H), 0.85 (t, *J* = 7.4 Hz, 3H). ^{13}C NMR (101 MHz, Chloroform-*d*): δ (ppm) = 209.51, 75.09, 67.82, 48.64, 45.50, 31.53, 25.57, 17.03, 13.71.[29]

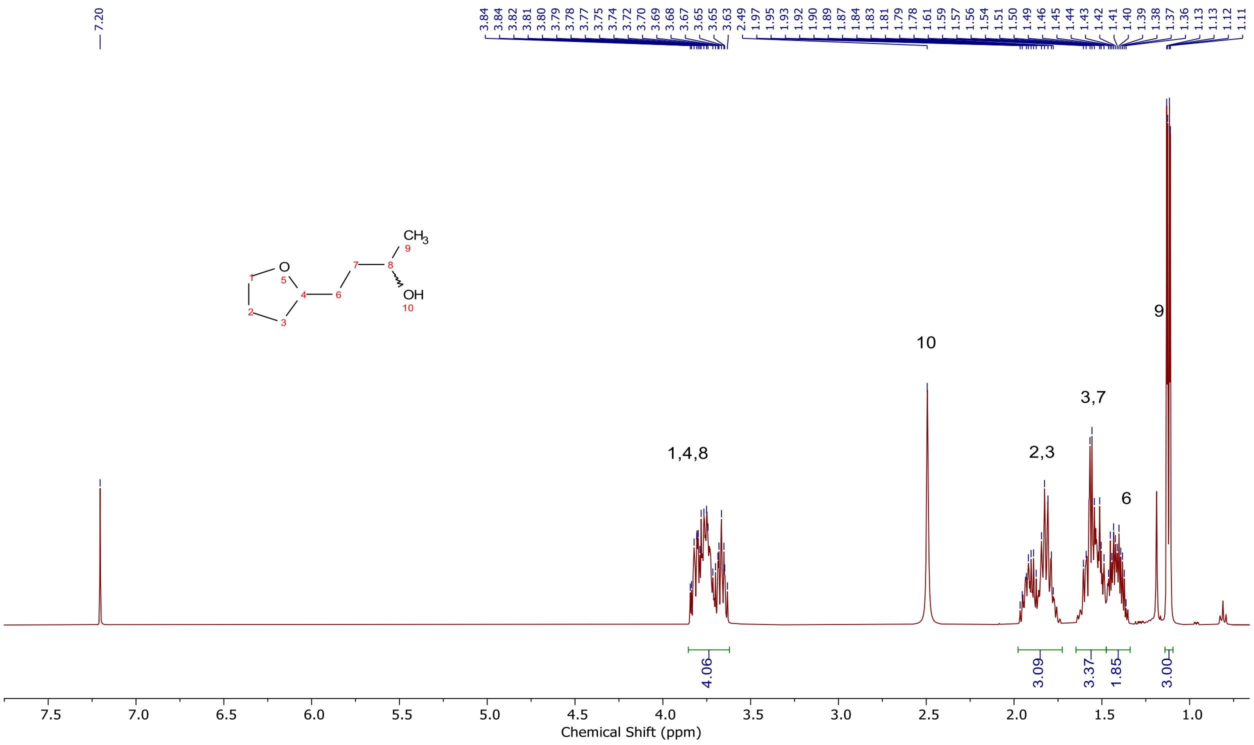

Figure S28. ^{1}H NMR spectrum of **1d** in $CDCl_3$.

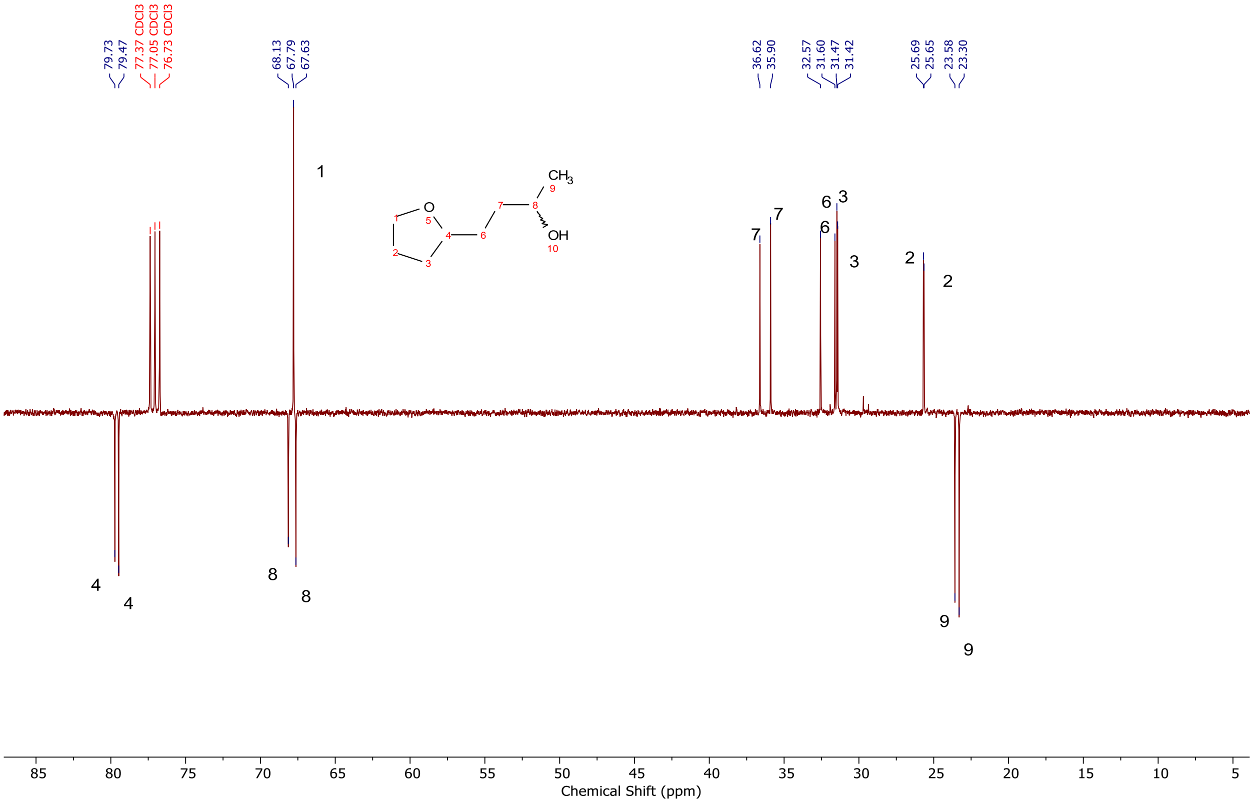

Figure S29. ^{13}C NMR spectrum of **1d** in $CDCl_3$.

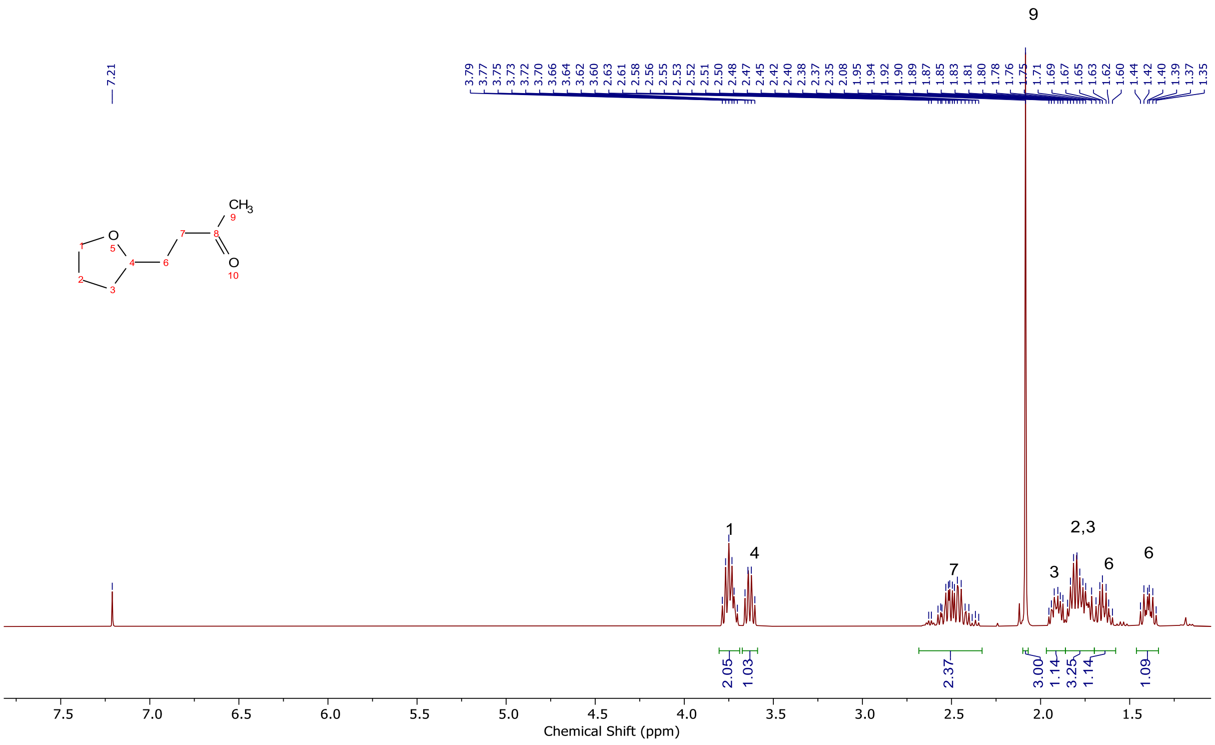

Figure S30. ^{1}H NMR spectrum of **1b** in $CDCl_3$.

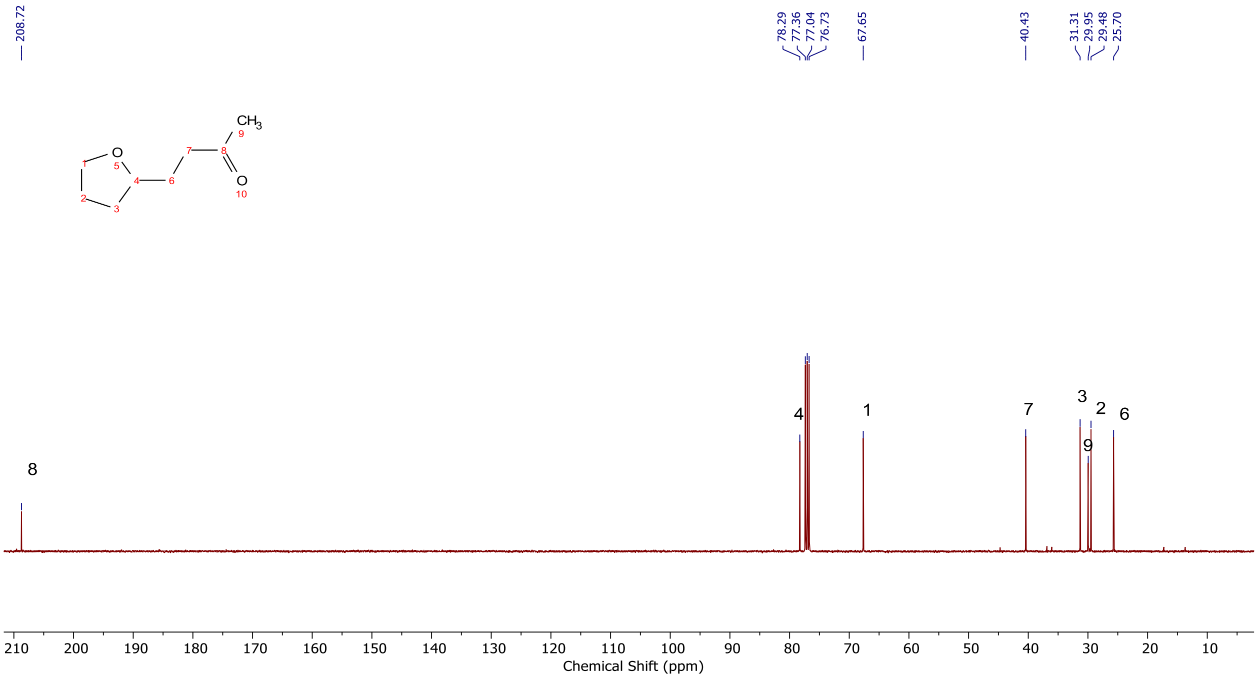

Figure S31. ^{13}C NMR spectrum of **1b** in $CDCl_3$.

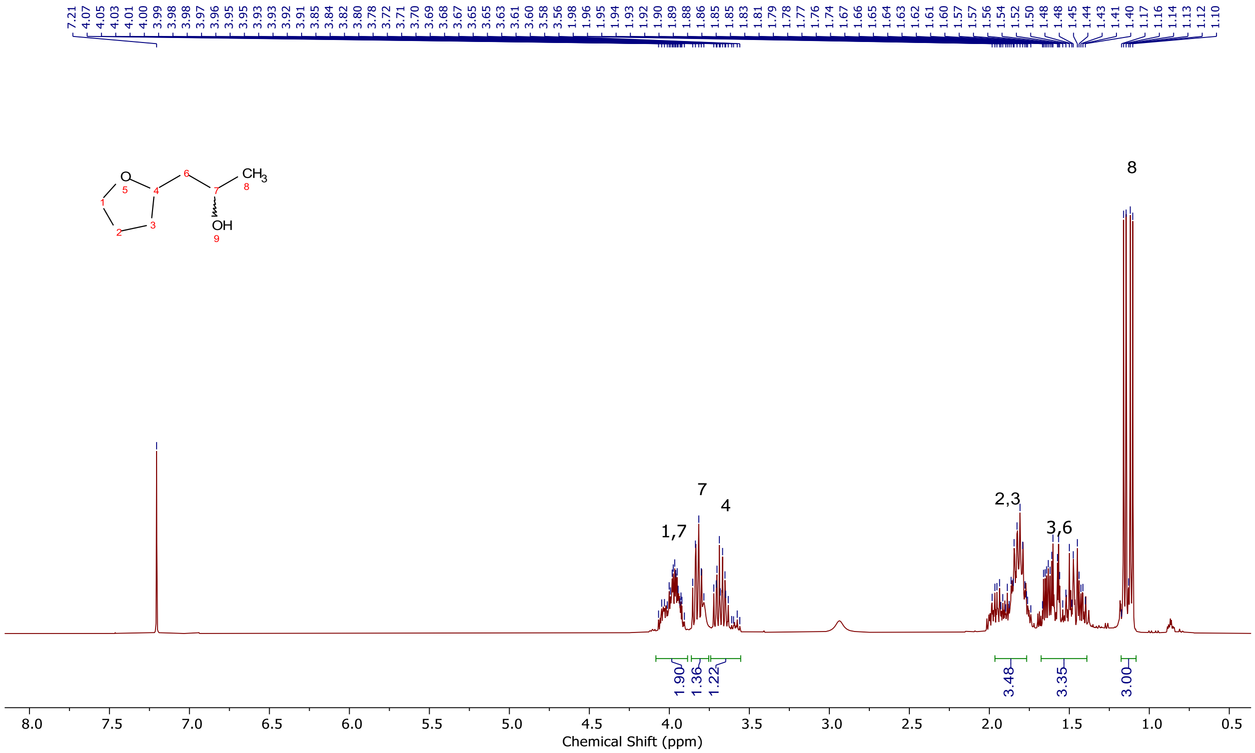

Figure S32. ^{1}H NMR spectrum of **2b** in $CDCl_3$.

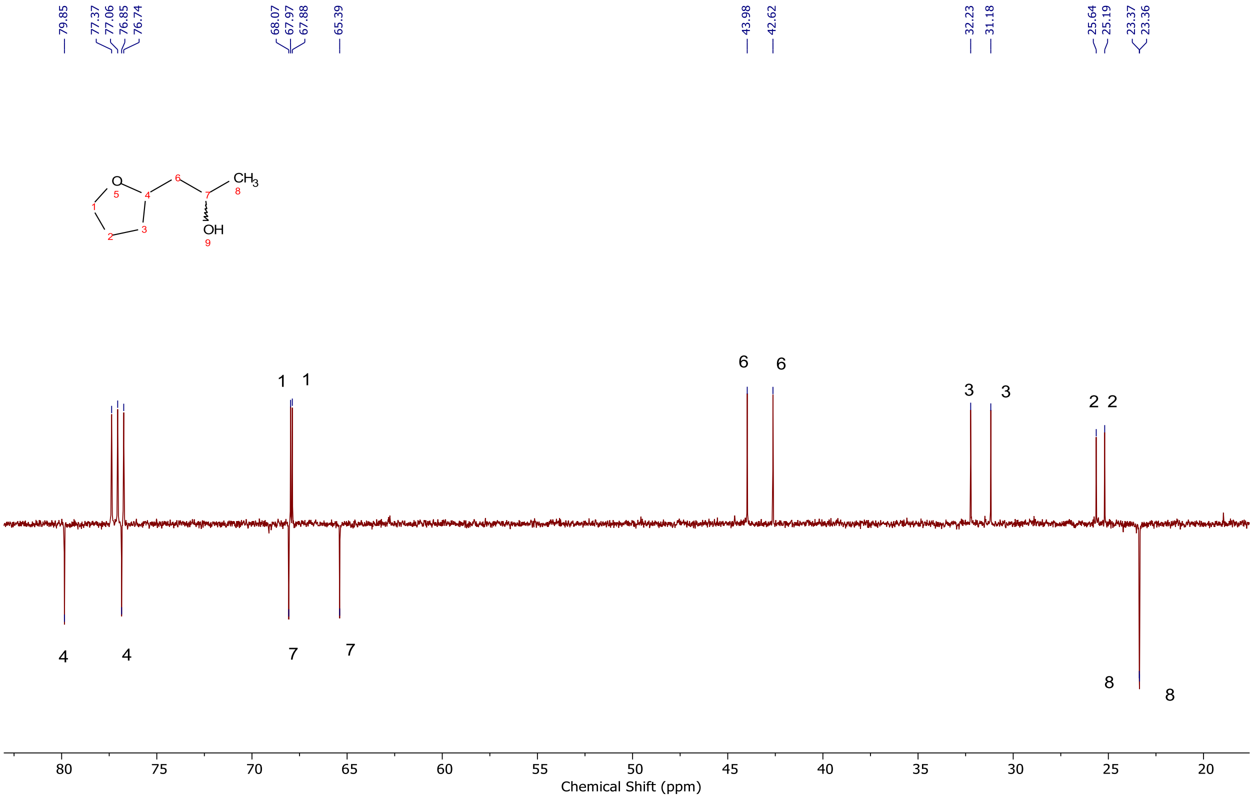

Figure S33. ^{13}C NMR spectrum of **2b** in $CDCl_3$.

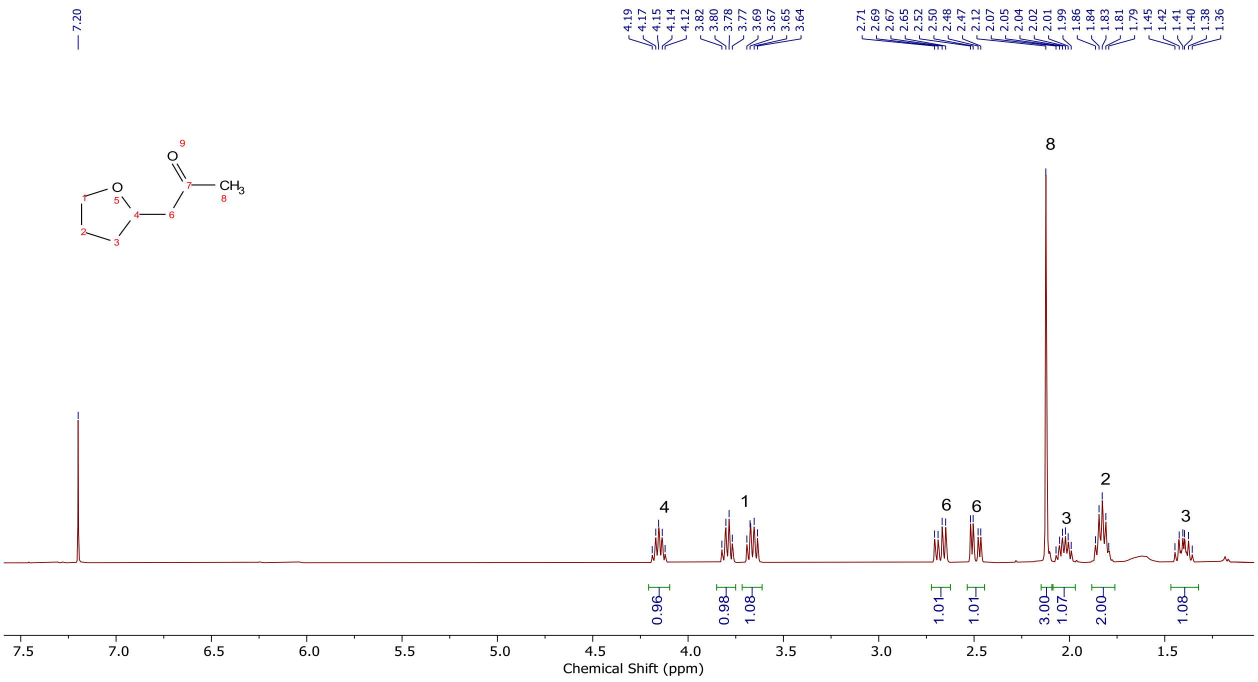

Figure S34. ^{1}H NMR spectrum of **2a** in $CDCl_3$.

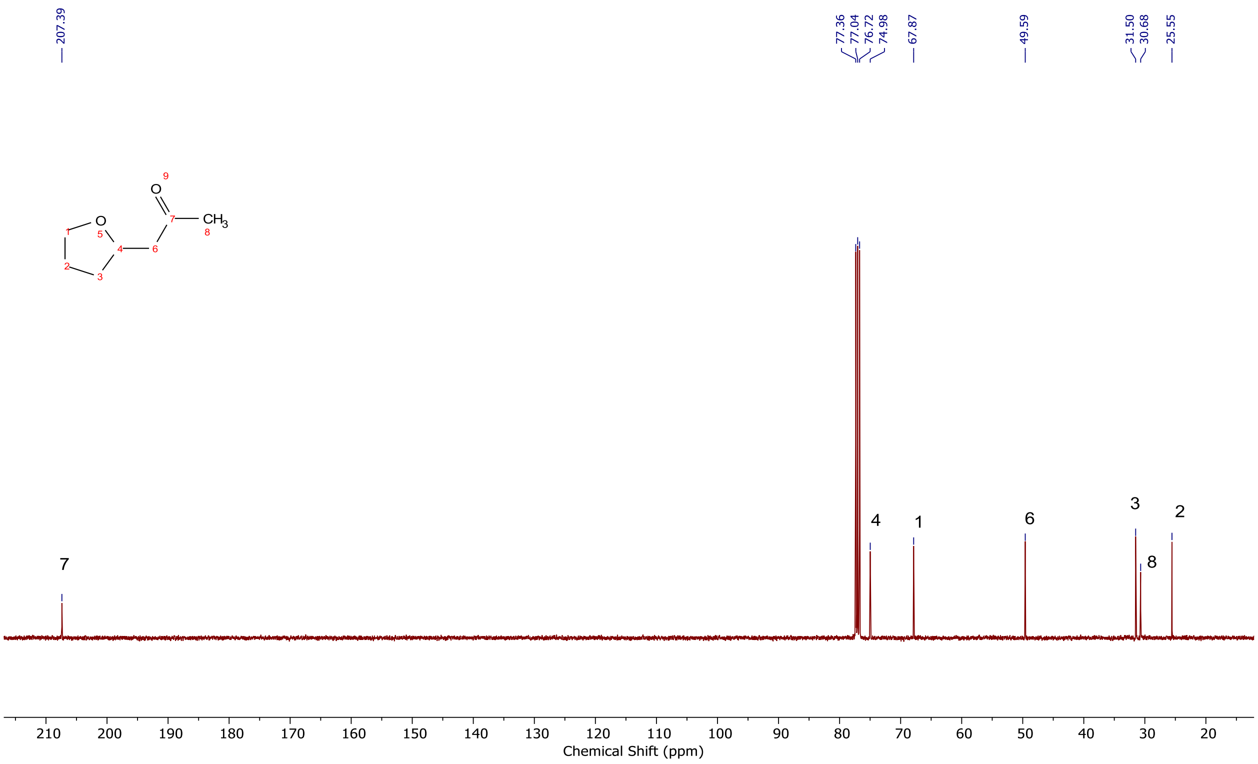

Figure S35. ^{13}C NMR spectrum of **2a** in $CDCl_3$.

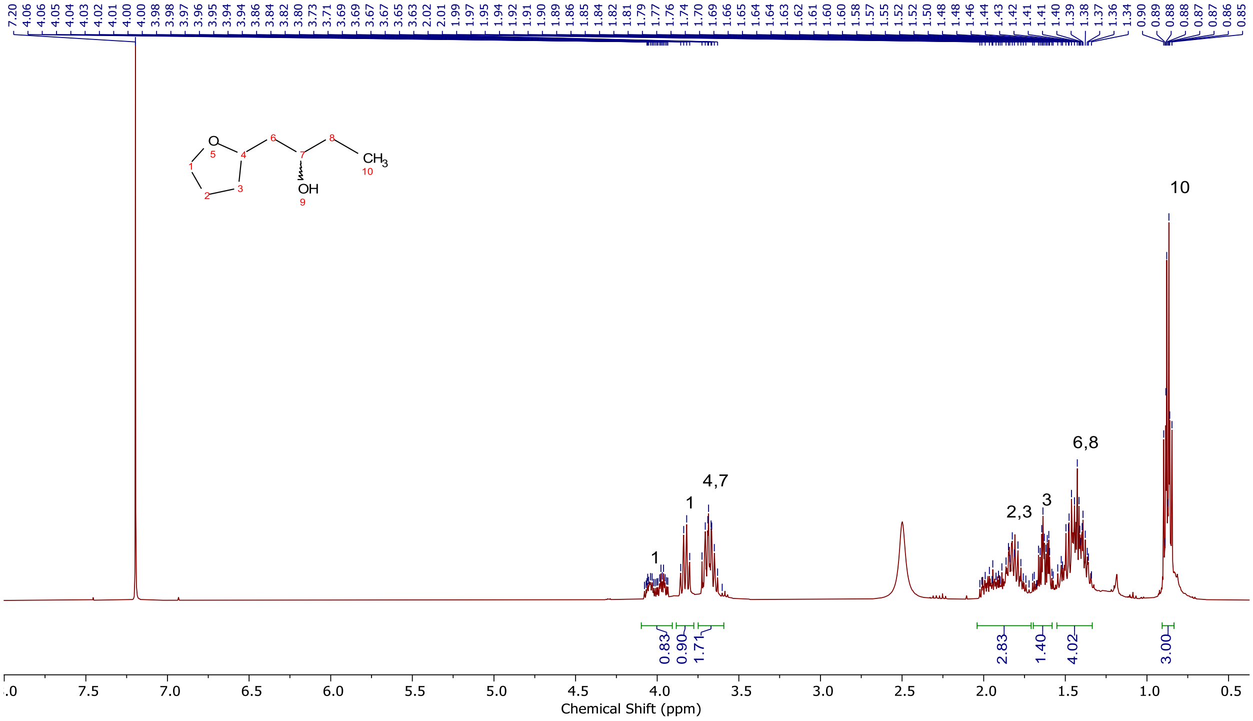

Figure S36. ^{1}H NMR spectrum of **3b** in $CDCl_3$.

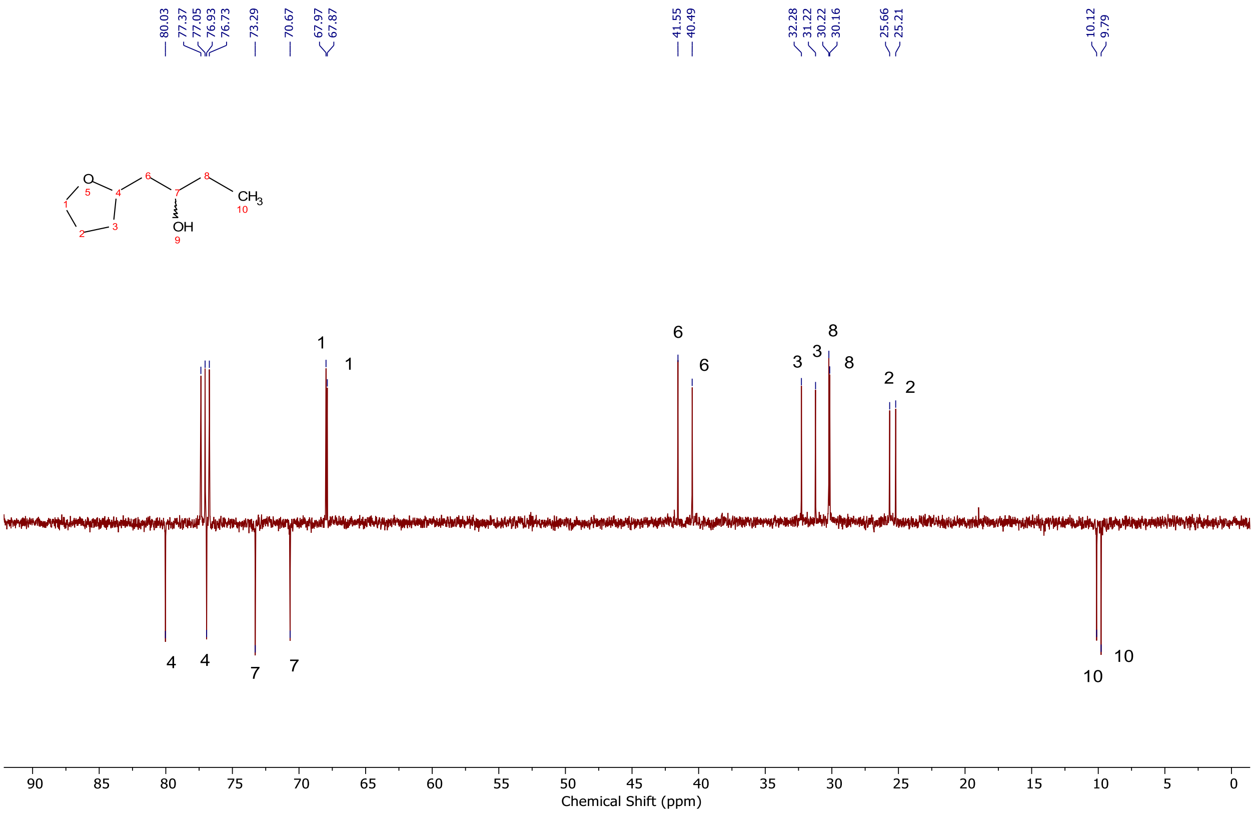

Figure S37. ^{13}C NMR spectra of **3b** in $CDCl_3$.

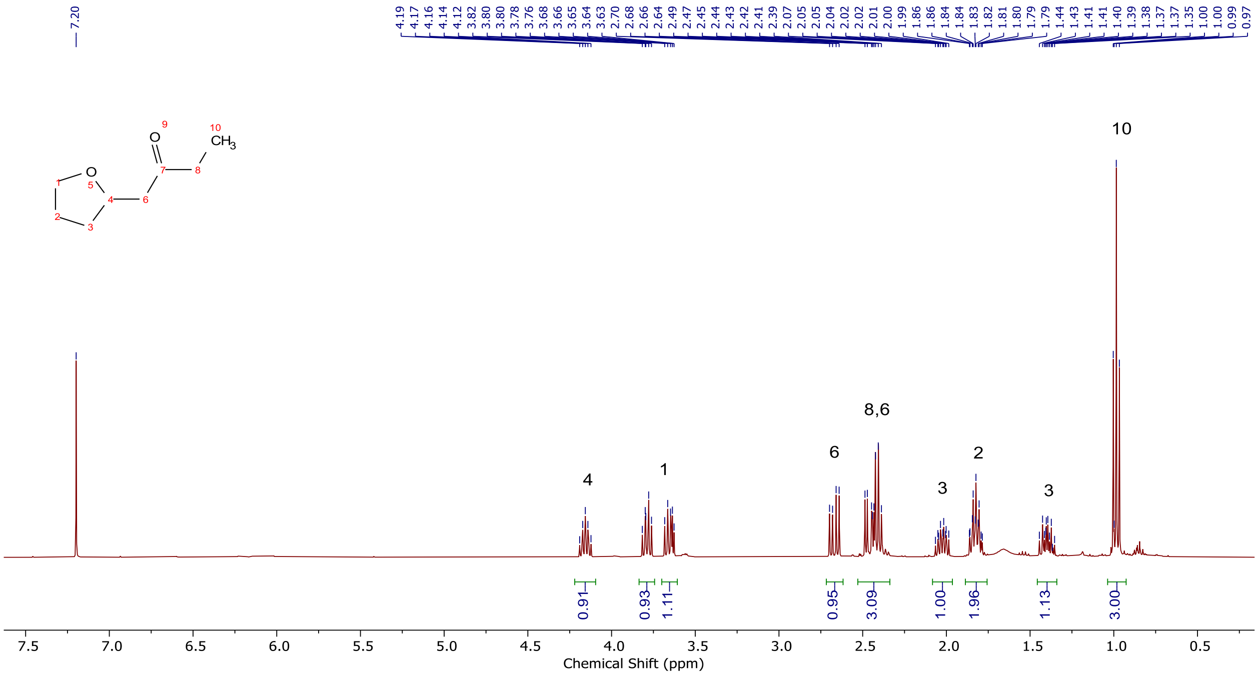

Figure S38. ^{1}H NMR spectra of **3a** in $CDCl_3$.

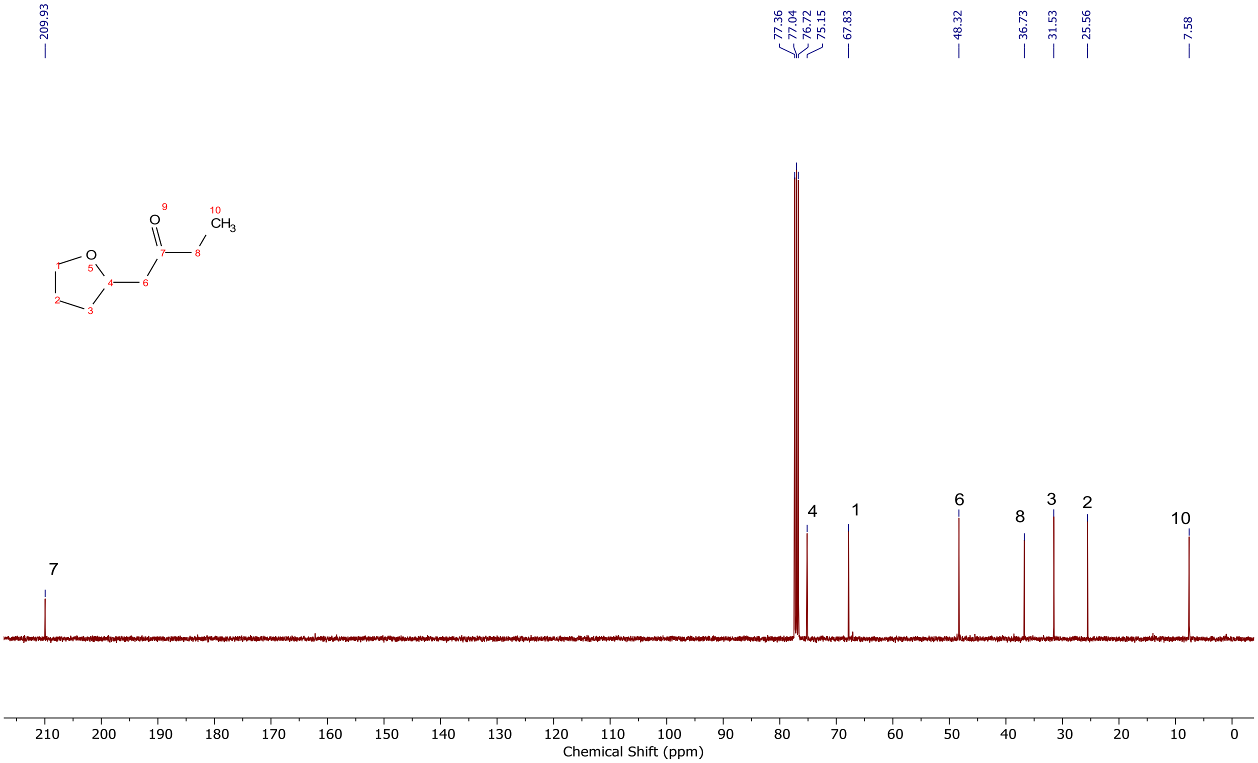

Figure S39. ^{13}C NMR spectra of **3a** in $CDCl_3$.

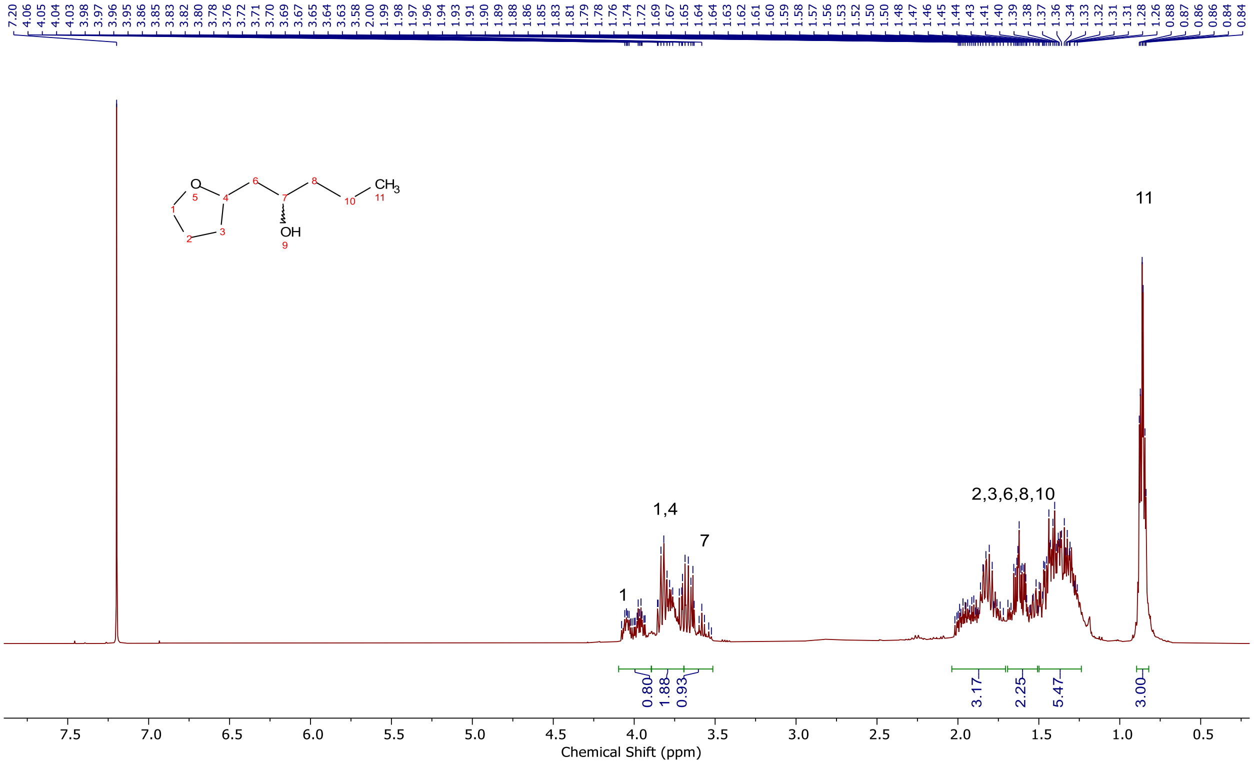

Figure S40. ^{1}H NMR spectra of **4b** in $CDCl_3$.

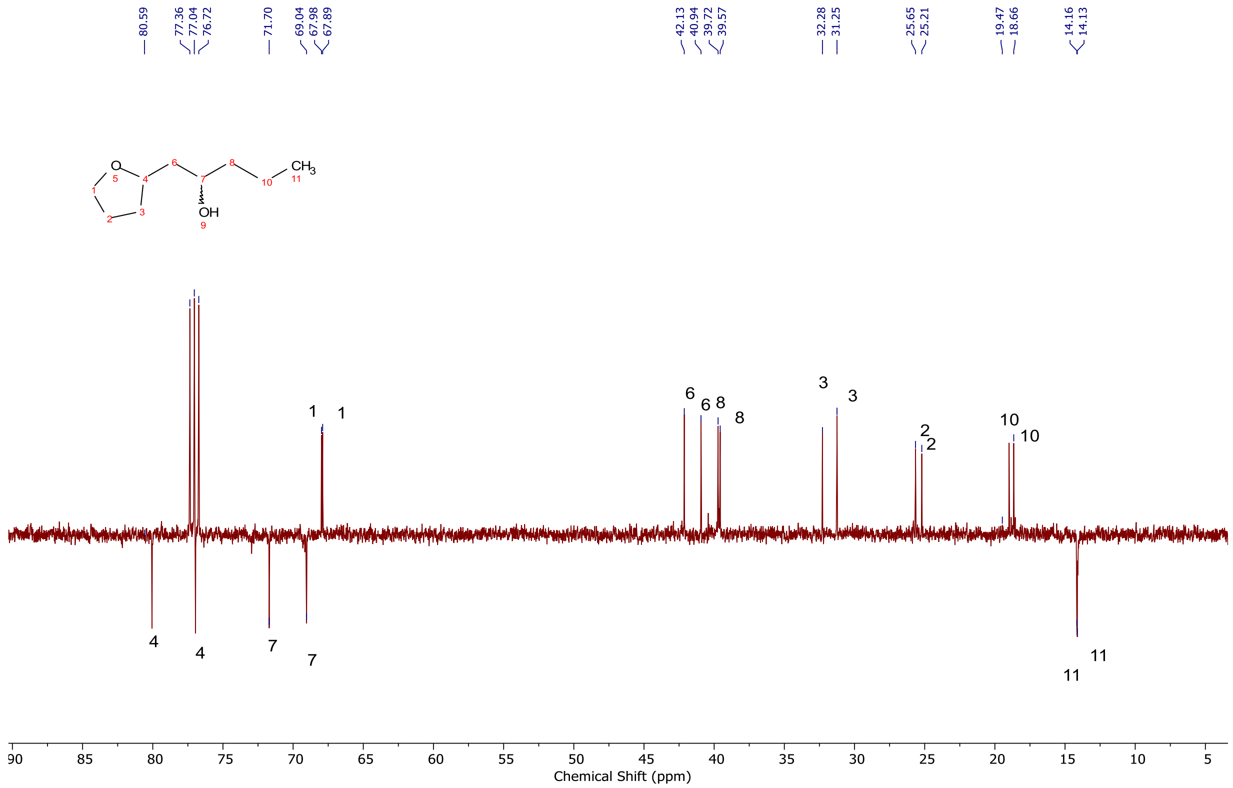

Figure S41. ^{13}C NMR spectra of **4b** in $CDCl_3$.

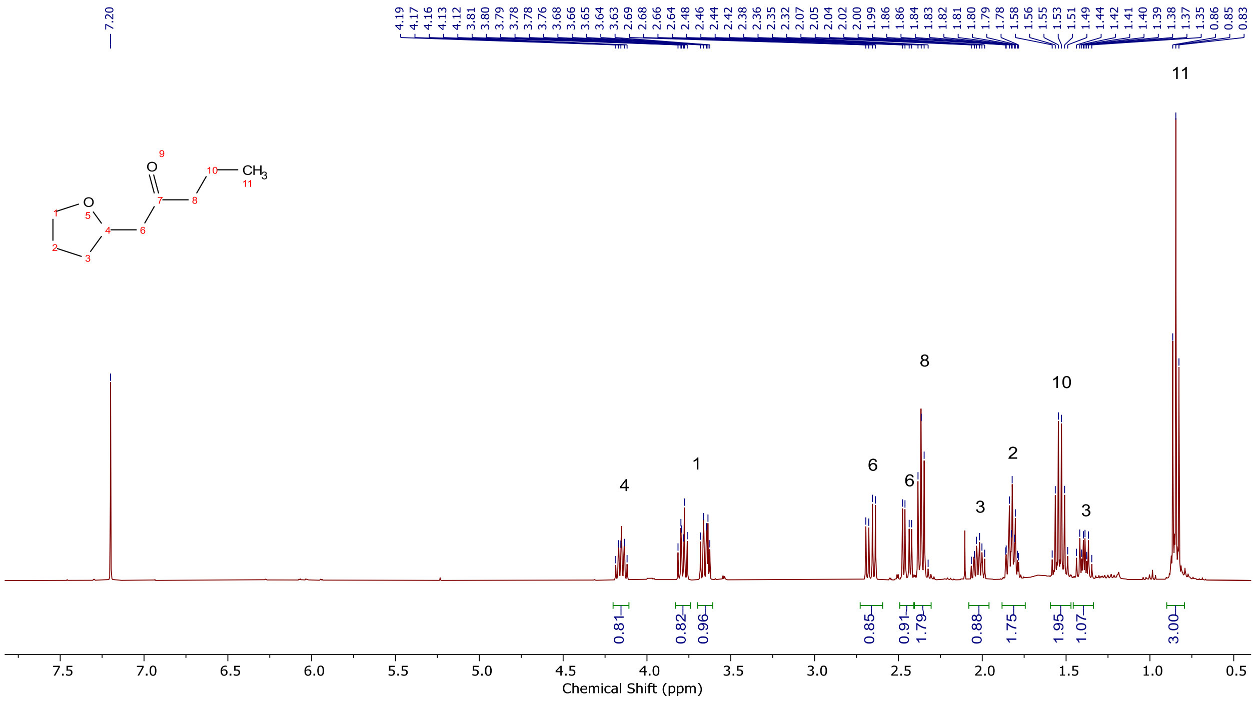

Figure S42. ^{1}H NMR spectra of **4a** in $CDCl_3$.

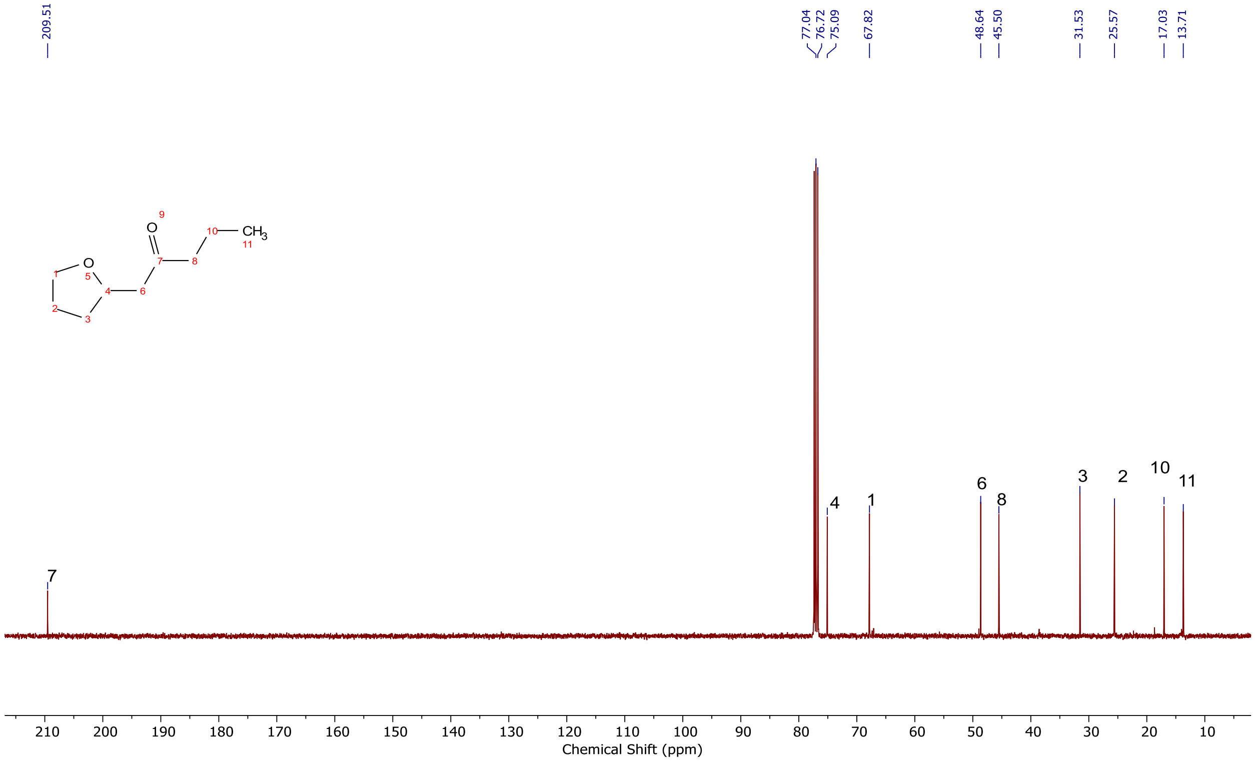

Figure S43. ^{13}C NMR spectra of **4a** in $CDCl_3$.

12 References

1. Wright, C. J.; Zhou, X. D. Computer-assisted area detector masking. *J. Synchrotron Radiat.* **2017**, *24*, 506-508.
2. Juhás, P.; Davis, T.; Farrow, C. L.; Billinge, S. J. L. PDFgetX3: a rapid and highly automatable program for processing powder diffraction data into total scattering pair distribution functions. *J. Appl. Crystallogr.* **2013**, *46*, 560-566.
3. Juhas, P.; Farrow, C. L.; Yang, X.; Knox K. R.; Billinge, S. J. Complex modeling: a strategy and software program for combining multiple information sources to solve ill posed structure and nanostructure inverse problems. *Acta Crystallogr. A: Found. Adv.* **2015**, *71*, 562-568.
4. Huang, B.; Kobayashi, H.; Yamamoto, T.; Toriyama, T.; Matsumura, S.; Nishida, Y.; Sato, K.; Nagaoka, K.; Haneda, M.; Xie, W.; Nanba, Y.; Koyama, M.; Wang, F.; Kawaguchi, S.; Kubota Y.; Kitagawa, H. A CO adsorption site change induced by copper substitution in a ruthenium catalyst for enhanced CO oxidation activity. *Angew. Chem. Int. Ed.* **2019**, *58*, 2230-2235.
5. Welter, E.; Chernikov, R.; Herrmann M.; Nemausat, R. A beamline for bulk sample x-ray absorption spectroscopy at the high brilliance storage ring PETRA III. presented in part at the AIP Conf. Proc. 2054, **2019**.
6. Dent, A. J.; Cibin, G.; Ramos, S.; Smith, A. D.; Scott, S. M.; Varandas, L.; Pearson, M. R.; Krumpa, N. A.; Jones C. P.; Robbins, P. E. B18: A core XAS spectroscopy beamline for Diamond. *J. Phys. Conf. Ser.* **2009**, *190*, 012039.
7. Dent, A. J.; Cibin, G.; Ramos, S.; Parry, S. A.; Gianolio, D.; Smith, A. D.; Scott, S. M.; Varandas, L.; Patel, S.; Pearson, M. R.; Hudson, L.; Krumpa, N. A.; Marsch A. S.; Robbins, P. E. *J. Phys. Conf. Ser.* **2013**, *430*, 012023.
8. Ravel, B.; Newville, M. Performance of B18, the core EXAFS bending magnet beamline at diamond. *J. Synchrotron Radiat.* **2005**, *12*, 537-541.
9. Giannozzi, P.; Baroni, S.; Bonini, N.; Calandra, M.; Car, R.; Cavazzoni, C.; Ceresoli, D.; Chiarotti, G. L.; Cococcioni, M.; Dabo, I.; Dal Corso, A.; de Gironcoli, S.; Fabris, S.; Fratesi, G.; Gebauer, R.; Gerstmann, U.; Gougoussis, C.; Kokalj, A.; Lazzeri, M.; Martin-Samos, L.; Marzari, N.; Mauri, F.; Mazzarello, R.; Paolini, S.; Pasquarello, A.; Paulatto, L.; Sbraccia, C.; Scandolo, S.; Sclauzero, G.; Seitsonen, A. P.; Smogunov, A.; Umari P.; Wentzcovitch, R. M. QUANTUM ESPRESSO: a modular and open-source software project for quantum simulations of materials. *J. Condens. Matter Phys.* **2009**, *21*, 395502.
10. Giannozzi, P.; Andreussi, O.; Brumme, T.; Bunau, O.; Buongiorno Nardelli, M.; Calandra, M.; Car, R.; Cavazzoni, C.; Ceresoli, D.; Cococcioni, M.; Colonna, N.; Carnimeo, I.; Dal Corso, A.; de Gironcoli, S.; Delugas, P.; DiStasio, R. A.; Ferretti, J. A.; Floris, A.; Fratesi, G.; Fugallo, G.; Gebauer, R.; Gerstmann, U.; Giustino, F.; Gorni, T.; Jia, J.; Kawamura, M.; Ko, H. Y.; Kokalj, A.; Kucukbenli, E.; Lazzeri, M.; Marsili, M.; Marzari, N.; Mauri, F.; Nguyen, N. L.; Nguyen, H. V.; Otero-de-la-Roza, A.; Paulatto, L.; Ponce, S.; Rocca, D.; Sabatini, R.; Santra, B.; Schlipf, M.; Seitsonen, A. P.; Smogunov, A.; Timrov, I.; Thonhauser, T.; Umari, P.; Vast, N.; Wu X.; Baroni, S. Advanced capabilities for materials modelling with Quantum ESPRESSO. *J. Condens. Matter Phys.* **2017**, *29*, 465901.
11. Manathunga, M.; Jin, C.; Cruzeiro, V. W. D.; Miao, Y.; Mu, D.; Arumugam, K.; Keipert, K.; Aktulga, H. M.; Merz, K. M.; Gotz, A. W. Harnessing the power of multi-

GPU acceleration into the quantum interaction computational kernel program. *J. Chem. Theory Comput.* **2021**, *17*, 3955-3966.
12. Perdew, J. P.; Burke K.; Ernzerhof, M. Generalized gradient approximation made simple. *Phys. Rev. Lett.* **1996**, *77,* 3865-3868.
13. Dal Corso, A. Pseudopotentials periodic table: From H to Pu. *Comput. Mater. Sci.* **2014**, *95*, 337-350.
14. Marzari, N.; Vanderbilt, D.; De Vita A.; Payne, M. C. Thermal contraction and disordering of the Al (110) surface. *Phys. Rev. Lett.* **1999**, *82*, 3296-3299.
15. Klimes, J.; Bowler D. R.; Michaelides, A. A critical assessment of theoretical methods for finding reaction pathways and transition states of surface processes. *J. Condens. Matter Phys.* **2010**, *22*, 074203.
16. Grimme, S.; Antony, J.; Ehrlich S.; Krieg, H. A consistent and accurate ab initio parametrization of density functional dispersion correction (DFT-D) for the 94 elements H-Pu. *J. Chem. Phys.* **2010**, *132*, 154104.
17. Zhao, P.; He, Y.; Cao, D. B.; Wen, X.; Xiang, H.; Li, Y. W.; Wang J.; Jiao, H. High coverage adsorption and co-adsorption of CO and H 2 on Ru (0001) from DFT and thermodynamics. *Phys. Chem. Chem. Phys.* **2015**, *17*, 19446-19456.
18. Lu, X.; Wang, W.; Deng, Z.; Zhu, H.; Wei, S.; Ng, S.-P.; Guo W.; Wu, C.-M. L. ethanol oxidation on Ru(0001) for direct methanol fuel cells: analysis of the competitive reaction mechanism. *RSC Adv.* **2016**, *6*, 1729-1737.
19. Louis Anandaraj, S. J.; Kang, L.; DeBeer, S.; Bordet, A.; Leitner, W. Catalytic Hydrogenation of CO2 to Formate Using Ruthenium Nanoparticles Immobilized on Supported Ionic Liquid Phases. *Small* **2023**, *19*, e2206806.
20. Chen, J.; Wang, Y.; Ding, X.; Huang, Y.; Xu, K. Magnetic solid-phase extraction of proteins based on hydroxy functional ionic liquid-modified magnetic nanoparticles. *Anal. Methods* **2014**, 6, 8358.
21. Bordet, A.; Moos, G.; Welsh, C.; License, P.; Luska, K. L.; Leitner, W. Molecular control of the catalytic properties of rhodium nanoparticles in supported ionic liquid phase (SILP) systems. *ACS Catal.* **2020**, 10, 13904−13912.
22. Kacem, S.; Emondts, M.; Bordet A.; Leitner, W. Selective hydrogenation of fluorinated arenes using rhodium nanoparticles on molecularly modified silica. *Catal. Sci. Technol.* **2020**, *10*, 8120-8126.
23. Kacem, S.; Qiao, Y.; Wirtz, C.; Theyssen, N.; Bordet A.; Leitner, W. Supercritical carbon dioxide as reaction medium for selective hydrogenation of fluorinated arenes. *Green Chem.* **2022**, *24*, 8671-8676.
24. Sisodiya-Amrute, S.; Van Stappen, C.; Rengshausen, S.; Han, C.; Sodreau, A.; Weidenthaler, C.; Tricard, S.; DeBeer, S.; Chaudret, B.; Bordet A.; Leitner, W. Bimetallic MxRu100−x nanoparticles (M = Fe, Co) on supported ionic liquid phases (MxRu100−x@SILP) as hydrogenation catalysts: Influence of M and M:Ru ratio on activity and selectivity. *J. Catal.* **2022**, *407*, 141-148.
25. Scanlon, J. T.; Willis, D. E. Calculation of flame ionization detector relative response factors using the effective carbon number concept. *J. Chromatogr. Sci*. **1985**, 23, 333–340.
26. Strohmann, M.; Bordet, A.; Vorholt A. J.; Leitner, W. Tailor-made biofuel 2-butyltetrahydrofuran from the continuous flow hydrogenation and deoxygenation of furfuralacetone. *Green Chem*. **2019**, *21*, 6299.

27. Morton, C. M.; Zhu, Q.; Ripberger, H.; Troian-Gautier, L.; Toa, Z. S. D.; Knowles, R. R.; Alexanian. E. J. C–H alkylation via multisite-proton-coupled electron transfer of an aliphatic C–H bond. *J. Am. Chem. Soc.* **2019**, *141*, 13253−13260.
28. Garavelas, A.; Mavropoulos, I.; Perlmutter P.; Westman, G. Stereoselective synthesis of tetrahydrofurans using intramolecular oxymercurations. *Tetrahedron Lett.* **1995**, *36*, 463-466.
29. Opalka, S. M.; Steinbacher, J. L.; Lambiris, B. A.; McQuade, D. T. Thiourea/proline derivative-catalyzed synthesis of tetrahydrofuran derivatives: a mechanistic view. *J. Org. Chem.* **2011**, *76*, 6503–6517.
30. Bordet, A.; El Sayed, S.; Sanger, M.; Boniface, K. J.; Kalsi, D.; Luska, K. L.; Jessop, P. G.; Leitner, W. Selectivity control in hydrogenation through adaptive catalysis using ruthenium nanoparticles on a CO2-responsive support. *Nat. Chem.* **2021**, *13*, 916-922.
31. Rauber, D; Philippi, F.; Morgenstern, B.; Zapp, J.; Kuttich, B.; Kraus, T.; Welton, T.; Hempelmann, R.; Kay, C. W. M. Dynamics, cation conformation and rotamers in guanidinium ionic liquids with ether groups, *Journal of Ionic Liquids*, **2023**, *3*, 100060.
